# Choline Acetate-, L-Carnitine- and L-Proline-Based Deep Eutectic Solvents: A Comparison of Their Physicochemical and Thermal Properties in Relation to the Nature and Molar Ratios of HBAs and HBDs

**DOI:** 10.3390/ijms26178625

**Published:** 2025-09-04

**Authors:** Luca Guglielmero, Angelica Mero, Spyridon Koutsoumpos, Sotiria Kripotou, Konstantinos Moutzouris, Lorenzo Guazzelli, Andrea Mezzetta

**Affiliations:** 1 Dipartimento di Farmacia, Università di Pisa, Via Bonanno 33, 56126 Pisa, Italy; luca.guglielmero@gmail.com (L.G.); angelica.mero@virgilio.it (A.M.); lorenzo.guazzelli@unipi.it (L.G.); 2 Scuola Normale Superiore, Piazza dei Cavalieri 7, 56126 Pisa, Italy; 3 Laboratory of Electronic Devices and Materials, Department of Electrical and Electronic Engineering, University of West Attica, 12244 Egaleo, Greece; skoutsoumpos@uniwa.gr (S.K.); skrypotou@uniwa.gr (S.K.); moutzouris@uniwa.gr (K.M.)

**Keywords:** deep eutectic solvents, rheological properties, optical properties, thermal properties, effect of molar ratio

## Abstract

The search for more sustainable alternatives to traditional organic solvents, in the frame of the green chemistry approach, is leading to an increasing interest toward the exploration of deep eutectic solvents (DESs), especially natural-based ones (NADESs). The great ferment in the use of DESs as innovative media for many applications and in the research of novel types of DESs is not matched by an equal rigor in their characterization and in the study of their physico-chemical characteristics. Nevertheless, it is evident how comparative studies encompassing the investigation of a wide range of properties in relationship with the DESs structures would be beneficial for a rational development of the field. In this work a panel of DESs featuring choline acetate, L-carnitine and L-proline as hydrogen bond acceptor constituents (HBAs) and ethylene glycol, glycerol and levulinic acid as hydrogen bond donor constituents (HBDs) in 1:2 and 1:3 molar ratios have been prepared and characterized. Their density, viscosity and optical properties have been thoroughly investigated at various temperatures, analyzing the influence of their composition in terms of type of HBA, type of HBD and molar ratio on their properties. All the proposed DESs have also been thermally characterized by TGA and DSC, providing a description of their thermal behavior in a wide range of temperature and determining their thermal stability and thermal degradation profile.

## 1. Introduction

The shift toward a more sustainable development model requires radical changes in the approach to chemistry, both within academic research and industrial applications. In recent years, the emergence of green chemistry guidelines has contributed to directing the scientific community toward a critical review of traditional chemical processes, stimulating the research of more sustainable and less environmentally impacting alternatives. Among the central challenges in this transition, the substitution of traditional organic solvents with safer alternatives represents one of the most actively pursued targets. Ionic liquids (ILs) and deep eutectic solvents (DESs) represent the most pursued alternative media to traditional solvents, beneficiating of lower volatility, flammability and toxicity characteristics. Nevertheless, it is necessary to keep in mind that the environmentally benign properties of this class of compounds should not be taken uncritically, since they are strongly related to their chemical nature [1,2,3,4]. DESs are defined as eutectic mixtures formed by combining two or more components—either ionic or molecular, solid or liquid—that function, respectively, as hydrogen bond donors (HBDs) and acceptors (HBAs). The term “deep” refers to the significantly lower experimentally observed eutectic point when compared to the predicted ideal behavior of the eutectic mixture [5]. Compared to ILs, DESs display easier preparation procedures, usually requiring no additional solvents, no purification steps and no byproducts resulting from their preparation, while still retaining interesting solvent properties and relatively low flammability and volatility [6]. Furthermore, the possibility of preparing DESs by only using naturally occurring or bio-derived constituents (natural DESs or NADESs) offers a particularly appealing opportunity from an environmental point of view [7]. Polyols like glycerol (Gly) and sugars represent the most employed natural-derived HBDs, together with natural or natural derived carboxylic acids. Among the latter ones, levulinic acid (LevA) is a diffused option, readily obtained from cellulose and widely available at low cost [8,9,10].

Finally, by selecting different HBA-HBD pairs and varying their molar ratios, it is theoretically possible to design DESs featuring specific properties in terms of density, viscosity, polarity, thermal stability, surface tension, ionic conductivity, phase behavior and refractive index [6,7,11,12]. Due to this versatility, DESs have found applications across a wide spectrum of scientific fields, ranging from their extensive use as solvents or catalysts in organic synthesis and as extraction media [13,14] to pharmaceutical and electrochemical applications [6,15,16].

Choline chloride certainly represents the most employed and studied HBA in the DESs panorama, and the properties of many systems based on this salt have been sufficiently investigated and reported in the literature [17,18,19]. Nevertheless, the research of task specific DESs with physico-chemical characteristics tailored for the selected application is steadily expanding the types of constituents taken into account. Choline acetate and L-carnitine represent interesting HBAs for the study of novel NADESs, both from an applicative and a structure–properties relationship study, since they are both structurally closely related to the much more diffused choline chloride and betaine HBAs, yet presenting meaningful differences in their properties and functionalities. Choline acetate-based DESs have been described as effective media for the dissolution of cellulose and, more generally, for the treatment of lignocellulosic biomasses [20,21,22]. However, interesting results concerning their use as solvents for the preparation of biodiesel [23] or for tribological applications are also reported in the literature [24]. On the other hand, L-carnitine and L-proline have been reported to represent effective media not only for the dissolution of biomasses and the extraction of polyphenols and flavonoids [25,26,27,28], but also for the absorption of SO_2_ and for the desulfurization of diesel [29,30]. Despite the growing applicative interest in DESs, there is still a general gap in the literature regarding thorough and systematic investigations into their properties. Gaining a more comprehensive understanding of how HBAs, HBDs, and their molar ratios influence DESs behavior is crucial for a conscious selection of the optimal DES for a specific application [31]. Furthermore, compiling a consistent and reliable dataset across a range of DESs compositions is essential for the improvement of predictive theoretical models, which can provide a powerful instrument for a deeper understanding of these systems and for a better comprehension of their structure–physicochemical properties relationship, thus accelerating their adoption in sustainable chemical practices [17].

Valuable efforts in this direction, both from an empirical and a theoretical point of view, have been made recently. Crespo et al. and Al-Dawsari et al. [32,33] studied the viscosity relationship with temperature for a wide variety of DESs, while Zhu et al. [32,34] expanded the investigation to other physico-chemical properties considering their relationship with the electronic properties of the adopted HBDs. On the other hand, the adoption of mixed experimental and computational methods has proven greatly beneficial for the comprehension of DES microscopic organization of DES systems and the prediction of their physico-chemical properties [35,36]. Naseem et al. and Migliorati et al. reported a theoretical study of the hydrogen-bonded supramolecular structure of choline-based DESs [37,38]. Spittle et al. presented a further deepened theoretical investigation of choline-based DESs, with a comprehensive discussion of the dynamics, anisotropies and time energy related properties of their hydrogen bond network [36]. Xin et al. presented a valuable example of the predictive possibilities given by a good understanding of DESs structures, calculating the activity coefficients of hydrophobic DES constituents through the UNIFAC group contribution method and managing to successfully estimate the density and the viscosity values of the outcoming DESs, in relationship with the temperature [39].

In this study, a series of DESs based on ChOAc, L-carn and L-pro, as HBAs, combined with EG, Gly and LevA, as HBDs, was prepared in different molar ratios (1:2 and 1:3) and their physico-chemical and thermal properties were systematically investigated. Density, viscosity, and optical characteristics were measured across a range of temperatures. Subsequently, thermal analysis was performed to evaluate their stability and their thermal behavior.

## 2. Results and Discussion

### 2.1. DES Preparation

A series of deep eutectic solvents (DESs) was prepared using choline acetate (ChOAc), L-carnitine (L-carn) and L-proline (L-pro) hydrogen bond acceptors (HBAs) and using ethylene glycol (EG), glycerol (Gly) and levulinic acid (LevA) as hydrogen bond donors (HBDs) (Table 1). These investigated solvent mixtures are undoubtedly less notorious compared to the more traditional choline chloride or betaine-based DESs. However, all the considered solvents (Table 1) have already been reported in various studies as DESs [18,23,29,40,41,42,43,44,45,46,47,48,49].

In 2023, Omar et al. [18] report an extensive database of DESs and their physical properties. Among the various mixtures reported, L-Carn:EG, Pro:LevA, Pro:Gly, ChOAc:EG, and ChOAc:Gly are reported as DESs. A mixture of ChOAc and LevA was reported as DES for the dissolution of hemicellulose present in Kraft pulp [8]. In addition, the DES L-Carn:LevA was used as an additive to increase the solubility of curcumin. DES based on levulinic acid produced the best results, outperforming lactic and pyruvic acid [42]. Similarly, DES L-Carn:EG and L-Carn:Gly have been used effectively to extract gingerols from ginger powder using ultrasonication [46].

The non-ideality of the eutectic mixture is not strictly related to the HBA:HBD ratio but it is intrinsic of the HBA-HBD couple (the exact melting point is strictly related to the ratio, while the phase diagram has to be non-ideal in throughout the range). Having this in mind, it can be stated that the herein proposed mixtures are eligible DES mixtures, even though they are not always present in compositions reported in the literature.

The DESs were prepared by mixing the selected hydrogen bond acceptors (HBAs) and hydrogen bond donors (HBDs) both in 1:2 and in 1:3 ratios (Table 1). The preparation was carried out at room temperature for ChOAc-based DESs and the L-Pro:LevA 1:3 DES, and at 40 °C or 60 °C for carnitine-based DESs and L-Pro:gly DES until homogeneous liquids were obtained. Given the significant impact of the water content on the physico-chemical characteristics of the final mixtures, ChOAc, EG, Gly, and LevA were thoroughly dried under vacuum prior to use. L-carn and L-pro were acquired in an anhydrous form, and no further drying was required. After the preparation, the resulting DESs were subjected to additional drying under vacuum, and their H_2_O content was determined using Karl Fischer titration prior to all measurements. The water content was found to be below 400 ppm for DESs containing LevA and between 400 and 600 ppm for those containing EG and Gly. The purity and correct molar ratios of the DESs were confirmed via ^1^H-NMR spectroscopy (Figures S1–S8, Supporting Information). Notably, the precipitation of white crystals was found to occur in the L-Pro:LevA 1:2 system after a few days from its preparation, even after extended stirring and heating up to 80 °C; thus, this system was excluded from further investigation.

### 2.2. Density

The study of DESs densities is particularly interesting due to its connection to other physical and optical properties [19]. Yet, differently from more commonly diffused DESs, as the ones based on choline chloride or betaine HBAs, density data concerning L-carnitine-, choline acetate- and L-proline-based systems are much more scarce in the literature and, to the best of our knowledge, density values are not available in the literature for any of the proposed DESs [18,50]. While the preparation of the DESs L-carn:EG 1:3, ChAcO:EG 1:2, ChAcO:Gly 1:2 and 1:3, and of L-pro:LevA and L-Pro:Gly DESs (respectively, in 1:2 and 1:2.5) has been reported [23,28,29,47,51], no density measurements were performed in the published studies.

**Figure 1:**
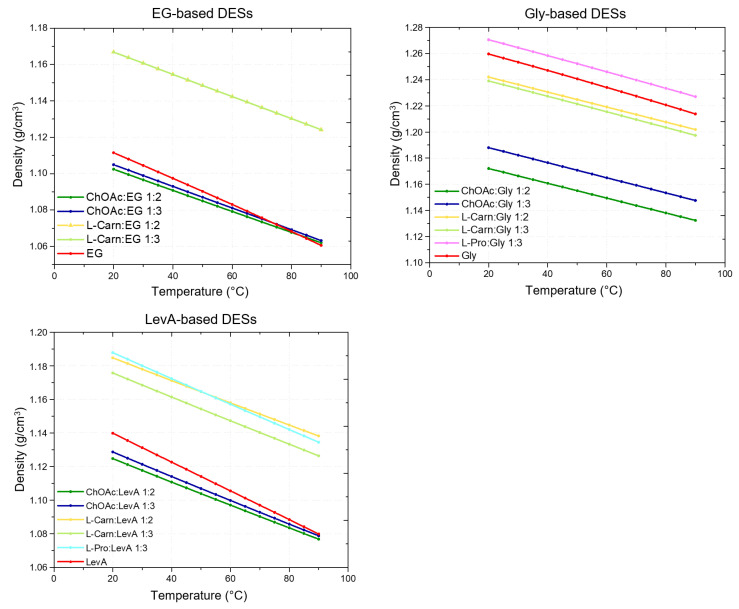
Densities of the considered DESs recorded in the 20 –90 °C range at intervals of 5 °C. The density plots have been grouped based on the HBD. From left to right: DESs containing EG, Gly and LevA as HBD.

For each investigated DES, its density was measured at 5 °C intervals over the 20–90 °C temperature range (Figure 1). The results are reported in Tables S1–S3 (Supporting Information). In all cases, the obtained densities exceeded that of water and exhibited a linear decrease with the growth of the temperature, as illustrated in Figure 1. The temperature dependence was accurately described by linear regression, yielding R^2^ values consistently greater than 0.9999 across the entire range (Figures S9–S15, Supporting Information). The corresponding slopes and intercepts of the linear fits for each DES and molar ratio are summarized in Table 2. Additionally, the densities of the pure HBDs were measured and compared to those of the corresponding DESs.

**Figure 2:**
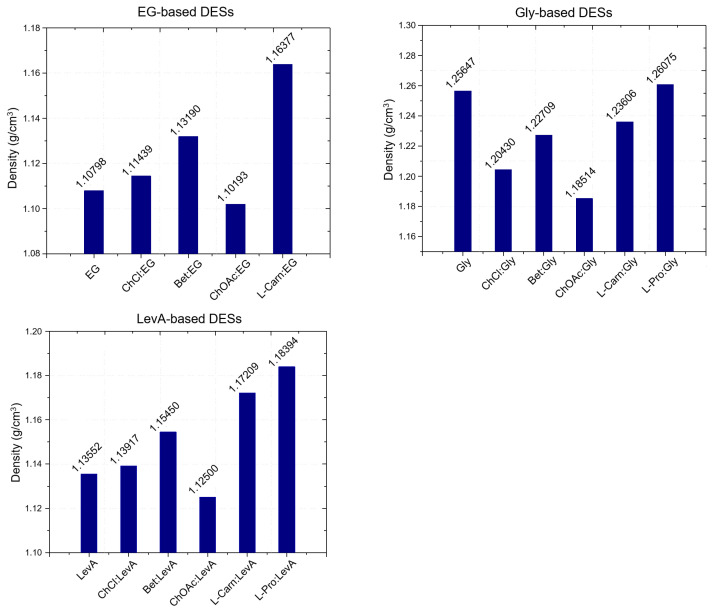
Densities of the DESs considered in this study (HBA:HBD 1:3) and recorded at 25 °C. The density plots have been grouped based on the HBD. From left to right: DESs containing ethylene glycol as HBD, DESs containing glycerol as HBD, DESs containing levulinic acid as HBD. Literature density values of choline chloride- and betaine-based DESs (HBA:HBD 1:3) have been reported for comparison.

From the obtained results it clearly emerges that both the HBA and the HBD, as well as their ratio, are important for defining the density of the resulting DES. Concerning the effect of the HBA, ChOAc was found to yield the less dense systems in comparison with the other tested HBAs, for each HBD. Interestingly, ChOAc:EG and ChOAc:LevA DESs were the only ones exhibiting lower density compared to the pure HBD. Only at higher temperatures (85 °C and 90 °C) was it possible to observe an inversion of this trend for ChOAc:EG systems, with the pure EG resulting slightly less dense (Figure 1). L-pro-based DESs, on the other hand, were found to be the densest ones for each employed HBD (Figure 1). An exception to this general behavior could be found at higher temperatures, with L-carn:LevA 1:2 becoming denser than Pro:LevA 1:3 at temperatures above 50 °C. L-pro:Gly 1:3 was also found to be the only Gly-based DES displaying higher density than pure glycerol. Finally, L-carn-based DESs manifested an intermediate behavior, with density values comprised between the extremes represented by ChOAc and L-pro systems for each HBD (with the only exception, above mentioned, represented by the temperature driven density inversion between L-carn:LevA 1:2 and L-pro:LevA 1:3). By comparing the density values at room temperature with literature data for similar DESs (Figure 2), it is possible to observe that the L-carn HBA yields denser DESs than the closely related betaine, for all the considered HBD (EG, Gly and LevA), while ChOAc consistently yield less dense DESs compared to those obtained with choline chloride (ChCl). The HBA density order can be resumed, including literature data, as ChOAc < ChCl < Bet < L-Carn < L-Pro.

Concerning the effect of the HBD, the reported data point out an evident direct relationship between the density of the HBD and the density of the resulting DES. For the same HBA, the DESs based on EG, the less dense HBD, have been found to display lower densities compared to DESs based on Gly and LevA, with the resulting density following the order EG < LevA < Gly (Figure 1 and Figure 2). Regarding the role of the HBA:HBD ratio, on the other hand, the obtained data do not point out any clear relationship with the density, and its effect appears strictly depending on the nature of both the HBA and the HBD. In the tested EG-based systems, the density differences between 1:2 and 1:3 HBA:HBD ratios were found to be minimal, with a slightly lower density observed for ChOAc:EG 1:2 compared to its 1:3 counterpart, while no significant differences were observable for the L-carn systems. When using Gly as HBD, on the other hand, ChOAc:Gly 1:2 displayed a significantly lower density compared to the 1:3 system, while a minimal increase in density was observed passing from the 1:2 to the 1:3 ratio for the L-carn:Gly system (Figure 1). LevA-based DESs displayed a similar behavior, but in this case, the density differences were more evident for the L-carn:LevA DESs and minimal for ChOAc:LevA DESs (Figure 1).

The increase in density observed passing from EG-based DESs to Gly-based ones, can be ascribed to the increase in the number of –OH functional groups in the hydrogen bond donor (HBD). As it is reported in the literature, this enhances the possibilities for hydrogen bond formation, thereby leading to higher system densities, underlining the critical role of intermolecular interactions in determining density [12,52,53]. This trend has been extensively documented by Basaiahgari et al., who observed that DESs composed of benzyltrialkylammonium chloride as the hydrogen bond acceptor (HBA) and EG as the HBD exhibited lower densities compared to systems using diethylene glycol, triethylene glycol, or Gly as HBD [54]. This rationalization is hardly applicable to the Lev-A systems, considering the different functional groups featured by levulinic acid, where instead of the hydroxyl groups, a carboxylic acid group and a carbonyl group are present. Neglecting other possible contributions to the density, it may be concluded that the hydrogen bonding property of Lev-A lies somewhere in between the ones of EG and Gly. Finally, the effect of the –OH groups on density can also be appreciated observing the density differences recorded between Bet and L-carn-based DESs, with L-carn-based systems, featuring a further –OH group on the HBA, manifesting a higher viscosity comapred to the related Bet DESs. Finally, the number of hydrogen bonds is affected by the HBA:HBD ratio, with higher numbers of hydrogen bonds reducing the free spaces available, and consequently increases the density of DESs [52,53]. According to the hole theory, the density and other physical properties of DESs are strongly influenced by the size, shape, and spatial arrangement of their molecular constituents. The molecular structure of DESs may contain voids or “holes”, the size of which directly affects the overall density of the system [55]. This concept may further explain the observed differences based on the HBAs: the smaller zwitterionic or neutral nature of betaine, L-carnitine and proline can result in more compact molecular arrangements than those formed with the bulkier salt, ChOAc and ChCl salt [19].

Finally, some observations can be made on the variation in density as a function of temperature. As already mentioned above, the obtained data are perfectly fitted by the linear model: *ρ* = *AT* + *B*, where *A* represents the slope of the linear dependence between the density and the temperature, and *B* the density at 0 °C (the obtained parameters are reported in Table 2, and the fittings are reported in Supporting Information, Figures S9–S15). A quantity often considered in the analysis of the temperature dependence of density is the thermal expansion coefficient *β*, defined as

(1) $$\beta =\frac{1}{V}{\left(\frac{\partial V}{\partial T}\right)}_{P}=-\frac{1}{\rho }{\left(\frac{\partial \rho }{\partial T}\right)}_{P}$$

which expresses the ease with which a liquid expands when heated under constant pressure. Considering the linear relationship found between density and temperature for all the considered systems, Equation (1) can be simply rewritten, in terms of A and B parameters, as

(2) $$\beta ={\left(\frac{B}{\left|A\right|}-T\right)}^{-1}$$

Unlike what was observed for density, where the influence of the HBA:HBD ratio was not clearly rationalizable, the effect of the ratio on *β* appears much more evident. For all six considered couples of DESs with equal HBA and HBD but different molar ratio, *β* grows passing from the 1:2 to the 1:3 molar ratio, meaning that the density of the considered 1:3 DESs reduces faster than the density of the corresponding 1:2 DESs along with the increase in temperature (with LevA systems being the ones where this effect is more evident). This trend is confirmed by literature data previously reported by our research group, where it was found to be applicable to the 1:4. molar ratio as well. Concerning the effect of the DES constituents on *β*, the role of the HBD appears to be preeminent. From the HBD imparting the lowest dependence of density from temperature to the highest, the trend can be summarized as Gly < EG < LevA. A dependence of *β* from the HBA type was also observed, even if not as evident as for the HBD. L-proline-based DESs displayed the highest β for each tested HBD, while with the same HBA and molar ratio, ChOAc was found to yield DESs with slightly higher *β* than the one obtained with L-carn. By comparing the results obtained for ChOAc and L-carn-based DESs in the current work and the literature data reported by our research group about ChCl and Bet-based DESs, it can be observed that the DESs featuring ChOAc as HBA display a higher *β* compared to ChCl-based DESs (in the same molar ratio with the HBD), while the DESs featuring L-carn as HBA display a lower *β* compared to Bet-based DESs (in the same molar ratio with the HBD).

**Figure 3:**
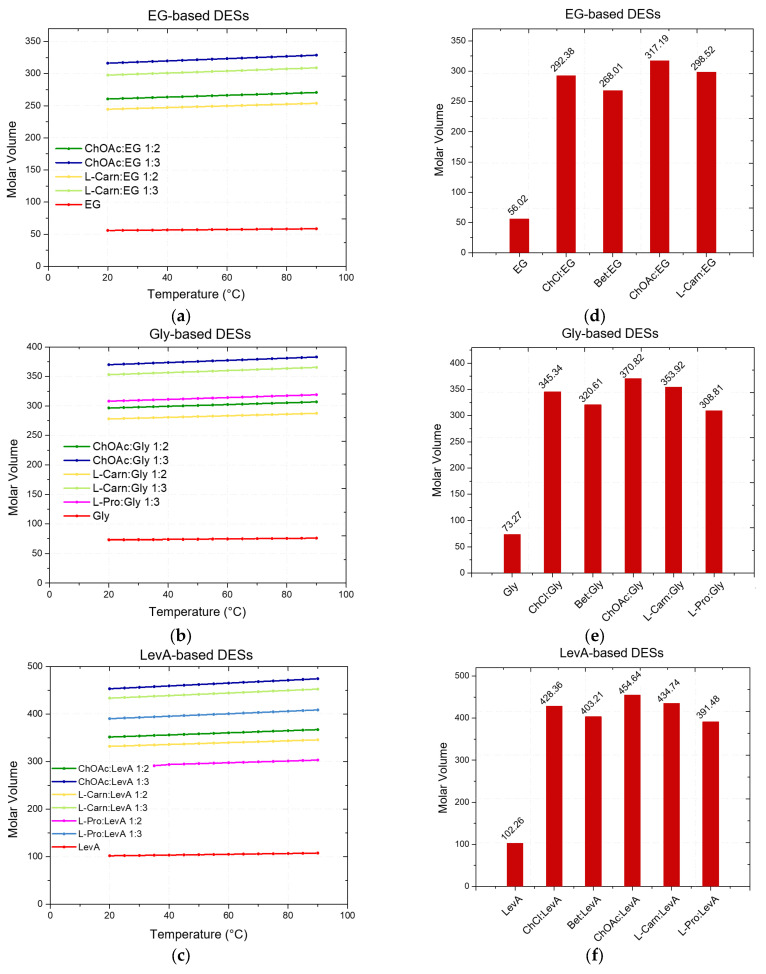
(**a**–**c**) Molar volumes (Vm) of the investigated DESs and HBDs in relationship with the temperature. (**d**–**f**) Molar volumes at 25 °C of the 1:3 HBA:HBD DESs considered in this work compared with literature data of ChCl and Bet-based DESs sharing the same HBDs and ratio.

Eventually, another quantity closely related to density worth mentioning is represented by the molar volume, described by the following equation

(3) $$V_{m}=\frac{M_{DES}}{\rho }$$

where and *ρ* and M_DES_ represent the density and the molar mass of the DES, respectively. M_DES_ was calculated as a sum of products between the molar mass of each DES component and its term of the molar ratio

(4) $$M_{DES}=n_{HBA}\cdot M_{HBA}+n_{HBD}\cdot M_{HBD}$$

for a DES with HBA:HBD ratio of n_HBA_:n_HBD_. The obtained results, displayed in Figure 3 (and summarized in Supporting Information, Tables S4–S6), show a strong dependence of the molar volume on the molar ratio of the DES (due to the term n_HBD_×M_HBD_), with the DESs 1:3 displaying a higher molar volume compared to the 1:2 DESs.

Besides the role of the molar ratio, the effects of both HBA and HBD on the definition of the molar volume of each DES can be easily summarized. Concerning the effect of the HBD, the trend follows the molar volume of the pure HBDs: EG < Gly < LevA, with DESs based on the same HBA but different HBD displaying increasing molar volumes according to this order. An equally clear trend can be found for HBAs. Including the data reported in the literature in a previous work, the trend L-Pro < Bet < ChCl < L-carn < ChOAc describes the observed trend for the molar volumes of DESs sharing the same HBD. Finally, following the decrease in density along with the temperature increase described above, the molar volume of each considered DES was found to increase when temperature increased.

### 2.3. Viscosity

Viscosity represents a fundamental parameter which contributes to the definition of the transport properties of a DES and, therefore, it is a key factor for the assessment of its applicability as a reaction or extraction medium, with a particular relevance from an industrial perspective. Similarly to what was previously mentioned about density, for viscosity, no data were available in the literature about the investigated DESs at the best of our knowledge [18]. The only exception is represented by the DES ChAcO:Gly 1:2, for which the value of viscosity at 50 °C has been reported [23].

**Figure 4:**
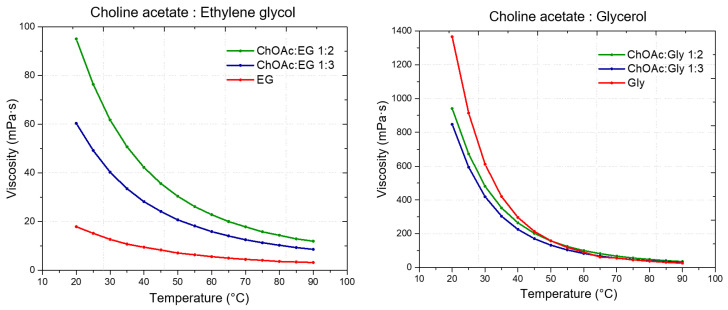

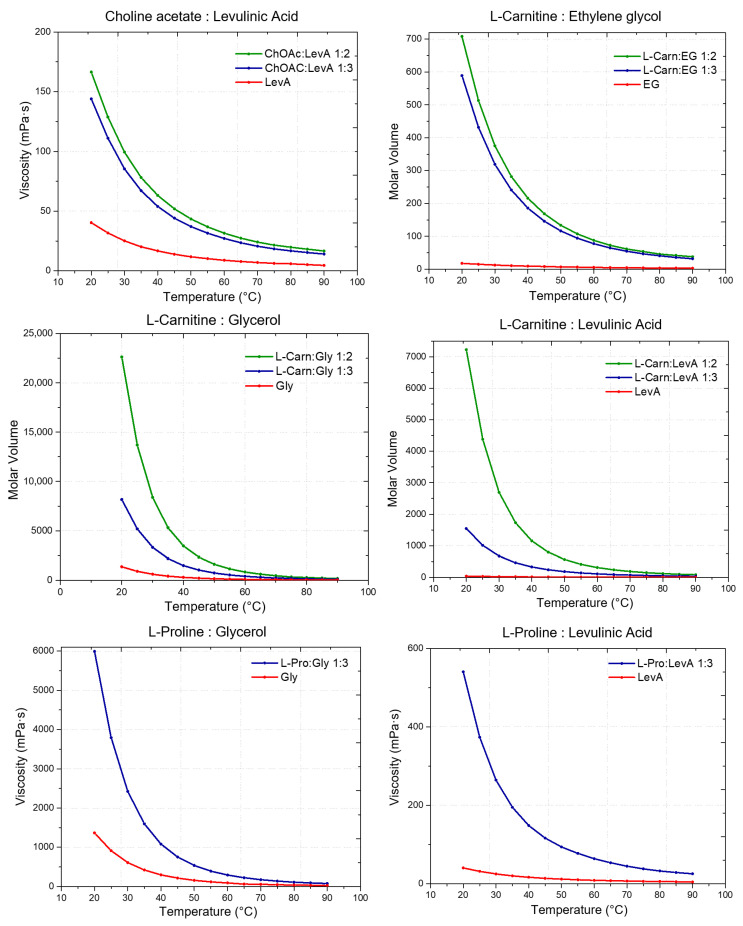
Viscosity vs. temperature plots of the indicated DES in the specified HBA:HBD molar ratio.

Initially, to assess the general flow behavior of the considered DESs, viscosity was measured in the shear rates range from 1 s^−1^ to 1000 s^−1^ (except of L-carn:Gly systems), at 20 °C. The high viscosity displayed by L-carn:Gly 1:2 and L-carn:Gly 1:3 DESs suggested the adoption of a narrower shear rate range (from 1 s^−1^ to 100 s^−1^), with both the DESs exhibiting Newtonian range in this interval. The flow curves of each considered DES showed a typical Newtonian behavior at lower rates, exhibiting constant viscosity values independent from the shear rate applied and from its duration. However, more viscous solvents such as glycol-containing DESs and L-Carn:LevA 1:2 did not display a Newtonian liquids behavior at higher shear rates, where a viscosity decrease could be observed, pointing out the rising of a shear-thinning behavior. This behavior is consistent with literature data [19,56] and it indicates that, at a microscopic level, DESs systems can be regarded as formed by liquid phase aggregates which become weakened or disrupted at high shear rates. For the test in function of temperature, the viscosity was analyzed at the constant shear rate of 50 s^−1^, where all samples exhibited Newtonian behavior. The temperature was increased in 5 °C incremental steps up to 90 °C, with data recorded at each step. The obtained values are illustrated in Figure 4 and reported in Tables S7–S9 (Supporting Information). Additionally, the viscosity of neat HBDs was measured and compared to that of the respective DESs.

**Figure 5:**
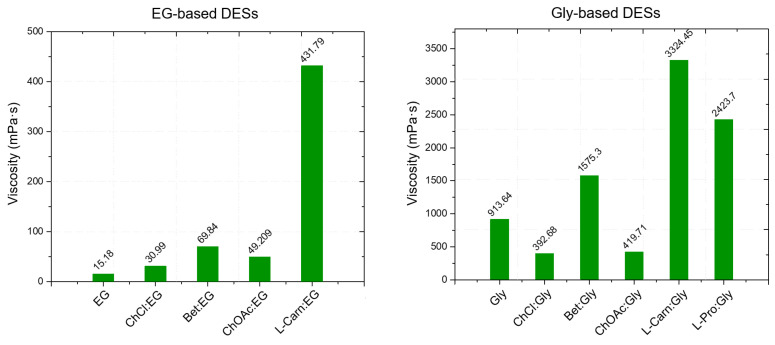

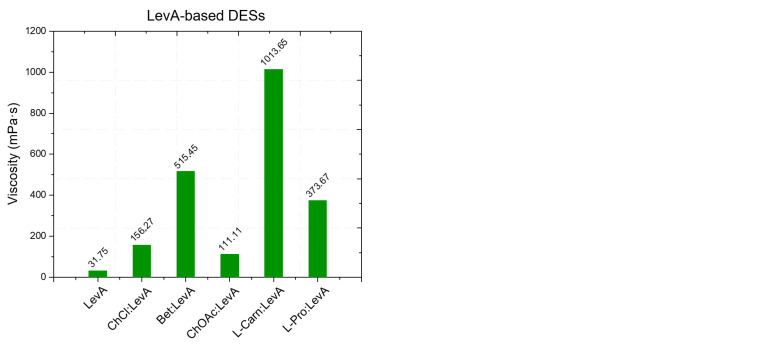
Viscosity at 25 °C of the DESs considered in this work compared with literature data of ChCl and Bet-based DESs sharing the same HBDs.

From the viscosity data obtained as a function of temperature, a strong dependence of the DES viscosity both from the HBA and the HBD, as well as from the molar ratio between the DES components is evident. Regarding the role of the HBD, within DESs with the same HBA, the viscosity was found to increase following the order EG < LevA < Gly, which is also the same order observed above about the relationship between HBD and density. Similarly to what observed for the density (where many DESs were found to display a lower value than pure glycerol) also during the viscosity study glycerol was the only tested HBD to not display a lower one compared to all the considered DESs, with ChOAc-based systems showing a lower viscosity. This behavior was reported in the literature also for ChCl systems (Figure 5). The role of the HBA in the definition of viscosity was found to be equally important, and the viscosity of the considered DESs was observed to increase according to the order ChOAc < L-pro < L-carn for each considered HBD. Finally, the effect of the molar ratio clearly emerged as a third fundamental element in the definition of the DES viscosity, with the observed trend being 1:3 < 1:2 for each considered HBA and HBD. The test performed at increasing temperature displayed spectacular drops of the viscosity values for Gly and LevA-based DESs, with the original severe viscosity differences becoming greatly dampened above 70 °C.

The viscosity of ChOAc:Gly 1:3 measured at 50 °C (157.91 mPa·s) was found to be higher than the value reported in the literature (80 mPa·s), which is reached at 65 °C. This discrepancy may be due to differences in the water content or, less likely, in thermostating inaccuracies [23]. Similarly to what was observed for density, the observed viscosity trends can be ascribed to the specific characteristics of the hydrogen bond acceptor (HBA) and donor (HBD) components of the DESs, particularly their molecular weight and size, which directly influence intermolecular interactions such as hydrogen bonding and van der Waals forces [12]. These factors significantly impact the overall mobility of the system. Again, the presence of further—OH groups on glycerol and L-carnitine with respect to ethylene glycol and betaine was found to lead to an increase in viscosity. Furthermore, the hole theory also provides a useful framework for the interpretation of these results. Abbott and colleagues applied this model to demonstrate how the presence of voids or “holes” within the liquid phase facilitates the movement of components within the hydrogen-bonded network [53]. They proposed that viscosity is more strongly influenced by volumetric factors, including steric effects, than by intermolecular interactions alone. According to this theory, the size and distribution of holes are closely linked to the structural characteristics of both HBA and HBD. The hole theory also accounts for the temperature dependence of viscosity. At lower temperatures, the average size of holes is smaller relatively to the molecular dimensions of the DES components, limiting their ability to move freely and thereby increasing viscosity [12]. In contrast, at elevated temperatures, the holes expand to sizes comparable to the DES components, promoting greater molecular mobility and consequently reducing viscosity [12].

The non-linear relation between viscosity and temperature is commonly modeled through the Arrhenius equation, which can be expressed in logarithmic form as

(5) $$\ln\left(\eta \right)=\ln\left(\eta_{\infty }\right)+\frac{E_{a}}{RT}$$

where η_∞_ represents the viscosity at infinite temperature, E_a_ is the activation energy for viscosity flow, R is the universal gas constant and T is the temperature in kelvin. *E*_a_ was calculated for each DES from Equation (5) using the fitting parameters, reported in Table 3, of the slope of the $\ln\left(\eta \right)$ vs. T^−1^ plots (Figures S16–S30, Supporting Information). The obtained data pointed out an evident correlation between the calculated values of E_a_ and the measured viscosity values. Less viscous DESs displayed lower *E_a_* values, with the lowest one for ChOAc:EG, while Gly-containing systems, which displayed higher viscosities compared to the other DESs considered in the study, showed higher E_a_ values, with the highest one observed for L-carn:Gly. LevA-based DESs, characterized by intermediate viscosities compared to EG and Gly-based systems, exhibited intermediate values of *E*_a_. Concerning the effect of the type of HBA, similarly to what was observed for the HBD, the same trend reported for viscosity was followed by *E*_a_. Despite the satisfactory data fitting provided by the Arrhenius model, a subtle yet visible deviation from linearity in the plotted data is still evident, as also confirmed by the R^2^ of the fittings, which calls for the use of a more optimized model.

The viscosity data were subsequently fitted using the Vogel–Fulcher–Tammann (VFT) model, which represents an alternative widely used approach to model the viscosity as a function of temperature

(6) $$\eta =\eta_{\infty }\cdot e^{\frac{B}{T-T_{0}}}$$

where *η*_∞_ is again the viscosity at infinite temperature, the parameter B substitutes the term *E*_a_/*R* and the parameter *T*_0_, which actually is the novel contribution of the VFT model compared to the Arrhenius one, virtually represents a temperature threshold below which any particle movement in the fluid is blocked, leading to infinite viscosity. The obtained fitting parameters are summarized in Table 4. By comparing the VFT fitting plots (Figures S16–S30, Supporting Information) and the related R^2^ values (for all DESs R^2^ > 0.999) with the corresponding Arrhenius model fittings, it clearly emerges that the VFT model provides a better description of the viscosity data.

### 2.4. Thermal Properties

**Figure 6:**
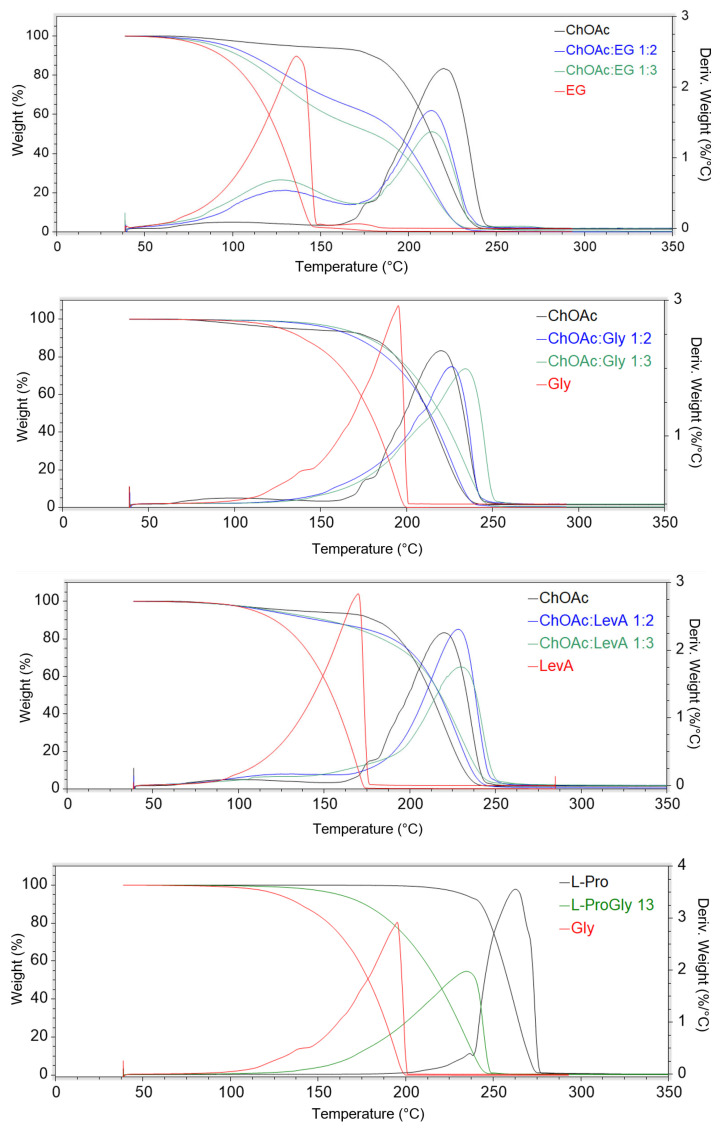

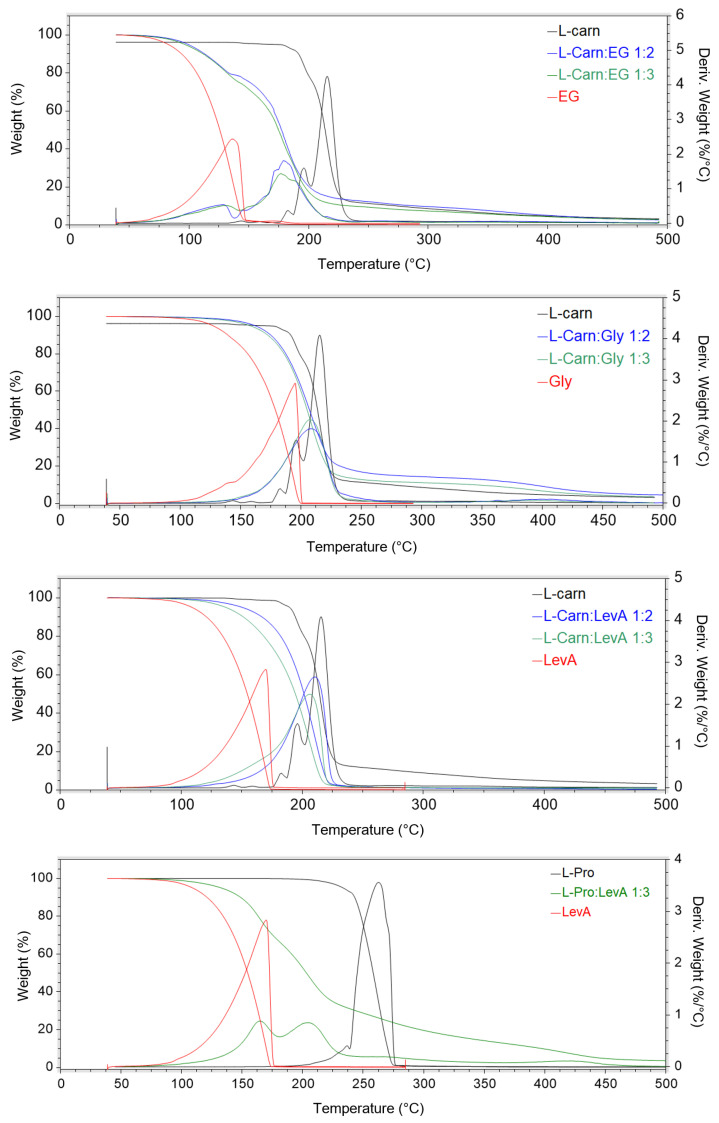
Thermograms of the DESs considered in this work compared with the pure HBA and HBD. Both the weight (%) vs. T and the *d*weight(%)/*d*T vs. T are reported.

The evaluation of the thermal properties of DESs represents a fundamental step, together with the study of the rheological properties, for the assessment of their applicability as solvents. Despite their importance, to the best of our knowledge, data on thermal stability and DSC behaviour have been reported in the literature only for the DESs ChAcO:Gly 1:2, ChAcO:Gly 1:3 and ChAcO:EG 1:2 [18,23]. The thermal stability of the considered DESs has been studied through thermal gravimetric analysis, performing a drying preliminary phase at 60 °C for 30 min, in order remove the water possibly absorbed from air during the sample preparation operations. The obtained thermograms are reported in Figure 6, and the results are summarized in Table 5 (the single thermograms are reported in Supporting Information, Figures S31–S45).

The obtained data indicate a preeminent role of the type of HBD on the thermal stability characteristics of the studied DES. This effect is particularly evident on the T_5%_ value, where the trend EG < LevA < Gly can be observed for each HBA. On the other hand, EG DESs are the only ones that manifest a clear HBD effect on T_onset_ and T_peak_, while Gly and LevA-based DESs sharing the same HBA displayed similar thermal stability. A strong effect of the HBD was observed in L-Pro-based DESs, where the LevA systems displayed thermal degradation parameter values 30–70 °C lower than the corresponding ones measured for the L-Pro:Gly DES. A schematization of the effect of the HBA nature on the thermal stability appears difficult due to the minor temperature variations generally observed for both T_5%_, T_onset_ and T_peak_ among the studied DESs sharing the same HBA but with different HBD. Furthermore, in many cases, T_5%_, T_onset_ and T_peak_ are affected in a different way by the change in HBA. L-carn DESs appear to exhibit T_onset_ and T_peak_ values about 10–20 °C lower than the corresponding ChOAc systems, but only with Gly and LevA HBDs, while no temperature differences can be observed for EG DESs. Conversely, only for the LevA HBD, L-carn DESs displayed a higher T_5%_ value (15–35 °C) when compared with the corresponding Gly-based DESs. L-Pro:Gly 1:3 displayed a thermal behavior exceptionally similar to the one observed for ChOAc:Gly 1:3, while the thermal stability of L-Pro:LevA 1:3, as commented above, was found to be peculiarly affected by LevA and cannot therefore be meaningfully compared with the other LevA-based DESs. The role of the HBA:HBD ratio, surprisingly, was found to play only a negligible role. If it is true that the differences in terms of T_onset_ and T_peak_ related to the HBA:HBD reported in the literature for similar DESs ratio are also minimal, the differences in terms of thermal degradation profiles observed in this work appears much less evident compared to literature data. The thermograms reported in the literature for ChCl- and Bet-based DESs featuring the same HBDs considered in the present work (EG, Gly and LevA) clearly displays multiple step degradation profiles, to the point that multiple onset and derivative peaks have been reported in the paper. Moreover, in the same study, perceivable differences in the height of each step could be appreciated in relation to the HBA:HBD ratio. Among the DESs investigated in the present work, only EG-based ones still displayed two recognizable degradation steps, even if they tend to appear much less separated (ChOAc DESs) or almost completely merged (L-carn) compared to what was reported in literature for ChCl and Bet. On the other hand, when Gly or LevA are used as HBDs, the degradation profile follows a single-step pathway (also visible in the *d*weight(%)/*d*T plot), where only a single peak is identifiable. The only exception to this behavior is represented by the DES L-Pro:LevA 1:3, which displayed two peaks in the derivative plot, even if close to each other and partially overlapped. The thermal behavior of ChOAc DESs was found to be particularly interesting, with T_peak_ values very close to the one of pure ChOAc (when EG was used as HBD) or even higher (when Gly and LevA were adopted). This is quite uncommon behavior since, typically, the T_peak_ of DES lies in between the T_peaks_ of the constituting HBA and HBD. The noteworthy temperature difference between the T_peak_ of the DES and the one of the constituting HBD observed for the Gly and the LevA systems (with the exception L-Pro: LevA 1:3), together with their single-step degradation profile and the lack of significant differences related to the HBA:HBD ratio, suggest the presence of much stronger hydrogen bonds in the DES investigated in this study compared to the more classic ChCl and Bet systems. These interactions were found to not only keep all the HBD molecules equally bound together in the DES system, but also to play a stabilizing influence on ChOAc. Unfortunately, the obtained data are not immediately comparable with the ones provided by Zhao et al. for ChAcO:Gly 1:2, ChAcO:Gly 1:3 and ChAcO:EG 1:2 since different thermal degradation parameters have been considered (T_5%_, T_onset_, and T_peak_ in the present work and T_10%_ by Zhao et al.). Nevertheless, the literature data appear consistent with the ones obtained in this work, confirming the much lower thermal stability observed for ChOAc:EG 1:2, and reporting similar thermal degradation data for the ChAcO:Gly-based DESs [18,23].

**Figure 7:**
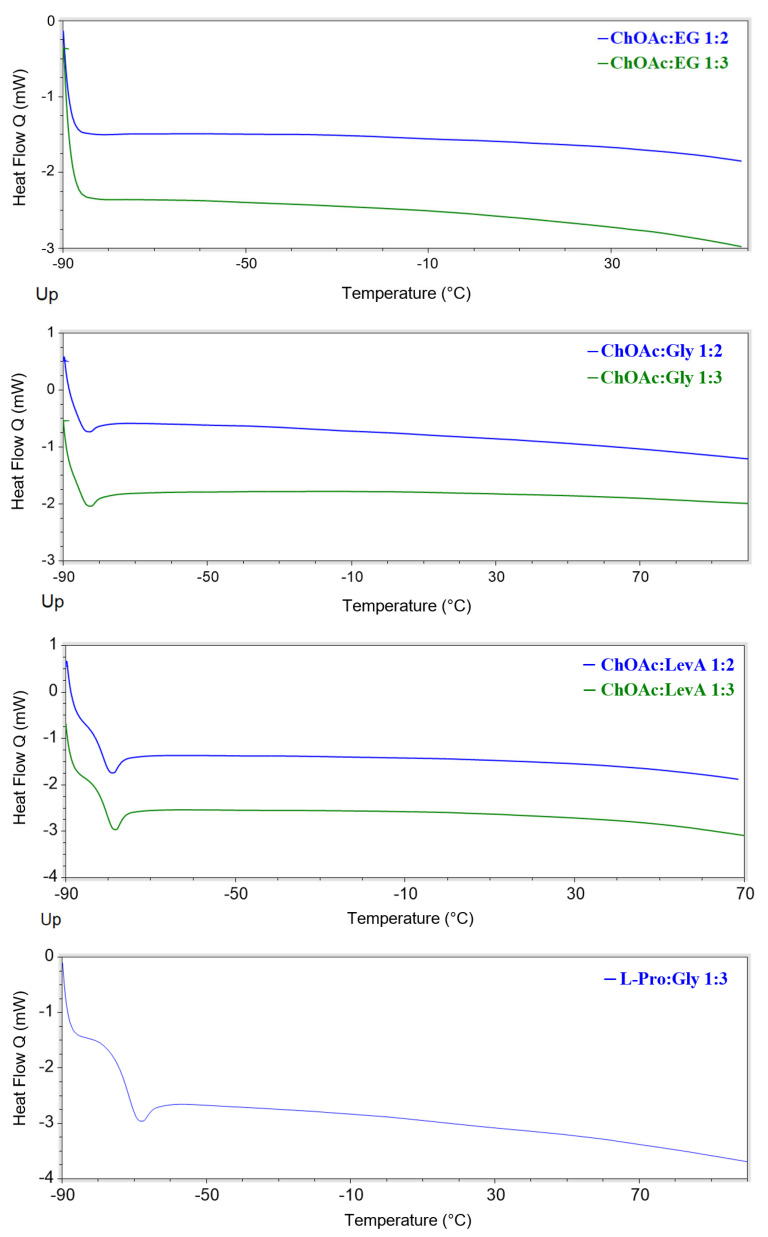

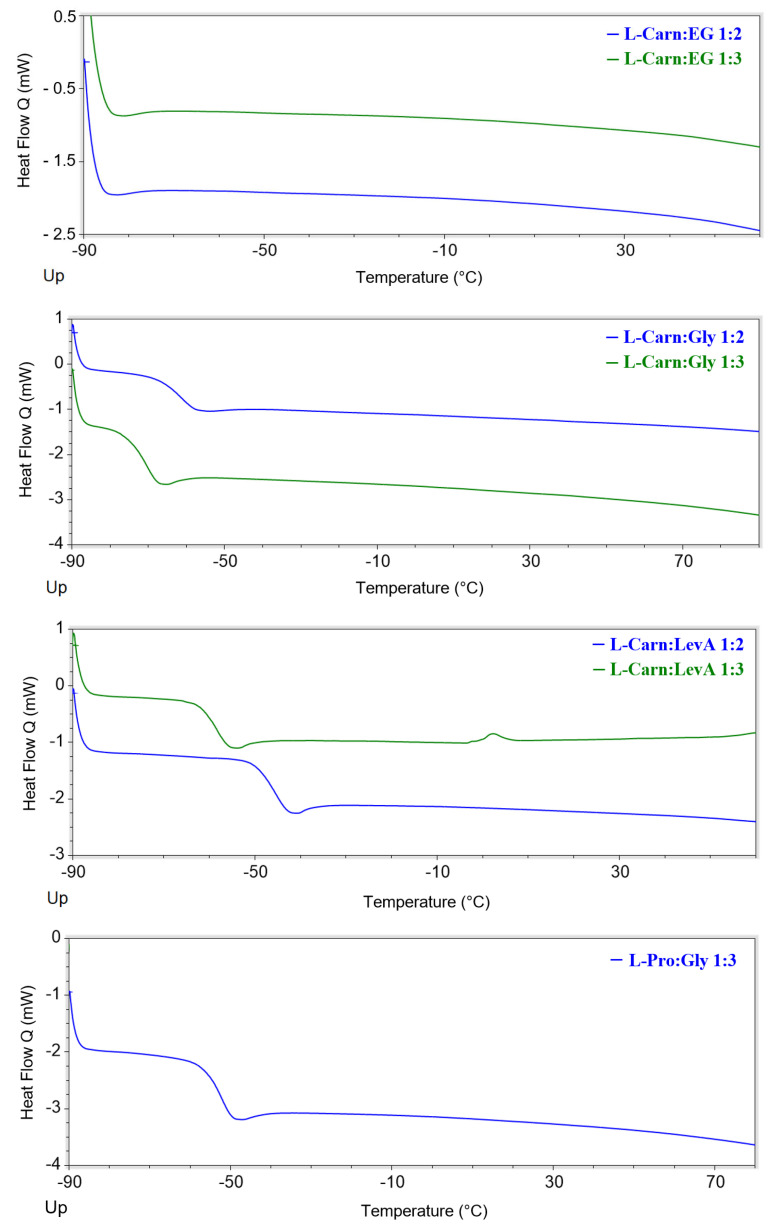
DSC thermograms of the investigated DESs.

All the investigated DESs displayed glass transitions as the only observable thermal phenomenon in the considered temperature range (Figure 7 and Figures S46–S59, Supporting Information). All the observed glass transitions took place at low temperature (in the range −45–−90 °C). The temperatures at which the transitions were detected were found to be related to both the HBA and the HBD, as well as the HBA:HBD ratio. Concerning the role of the HBD, it is immediately noticeable that EG-based DESs are the only ones, irrespectively of the associated HBA, to not display any thermal phenomenon. On the other hand, for each selected HBA, Gly-based DESs displayed lower T_g_ values. The observed T_g_ trend can be therefore summarized as EG << Gly < LevA. The effect of the HBA:HBD ratio appears to be depending on the type of HBA. L-Carn DESs manifested a lowering of the observed T_g_ passing from the 1:2 to the 1:3 system (of about 10 °C), while ChOAc DESs displayed an opposite behavior, even if in this second case the temperature differences were minimal. Finally, the influence of the HBA appears to be the most evident one, with L-carn systems displaying T_g_ values 10 °C to 35 °C higher than the corresponding ChOAc DESs. L-Pro DESs T_g_ values sit in between the extremes set by L-carn and ChOAc, but displaying values closer to the ones obtained for the L-carn DESs. Differently from what was reported by Zhao et al., during the current study, for all of the considered DESs, no melting points were detected during the DSC analysis. Conversely, no glass transitions are reported in their paper, due to these phenomena taking place out of their temperature range of investigation [23].

### 2.5. Refractive Index

The refractive index is the most fundamental optical constant, as it governs how light propagates through a material and underpins the design and optimization of optical components such as lenses, coatings, and sensors. In physical chemistry, it plays a key role in establishing correlations with properties like density, viscosity and surface tension through empirical or semi-empirical models, and serves as a practical tool for evaluating purity, water content, and overall composition in liquid mixtures. Despite its broad applicability, refractive index data for deep eutectic solvents remain limited [57]—particularly regarding their wavelength dependence [40].

In the present work, the refractive index of the DESs under investigation was measured at five different wavelengths: three in the visible spectral range (450 nm, 532 nm, 632.8 nm) and two in the near-infrared (940 nm and 1551 nm). Measurements were collected at each wavelength across four different temperatures ranging from 40 to 100 °C, in increments of 20 °C. The temperature step was not further reduced to prevent prolonged measurement times and to ensure that water absorption did not impact the results. This precaution was taken because the liquid holder cannot be sealed airtight, leaving the pre-dried sample partially exposed to environmental moisture. For the same reason, the measurement temperature was not lowered below 40 °C.

Experimental values of the refractive index (n), tabulated in Table S10 (Supporting Information), were fitted to a dispersion relation, which captures simultaneously the wavelength (λ) and temperature (T) dependence, according to

(7) $$n\left(\lambda ,T\right)={\left[1+\frac{s(T)\cdot \lambda^{2}}{\lambda^{2}-\lambda_{uv}^{2}}+d\cdot \lambda^{2}\right]}^{1/2}.$$

In the previous relationship, which has been used with other DESs [40] and ionic liquids [58] before, the coefficient $\lambda_{uv}$ signifies an anticipated absorption peak in the ultraviolet range, s(T) quantifies the resonance strength associated with this peak, while the third coefficient, d, assists in adjusting the refractive index values towards longer wavelengths. Note that the temperature dependence is incorporated into the coefficient s(T), which is given by

(8) $$s\left(T\right)=s_{1}+s_{2}\cdot T.$$

Computed values for all four fitting coefficients ($s_{1}$, $s_{2}$, $\lambda_{uv}$ and d) are shown in Table 6. The quality of fit is confirmed by R-squared (and adjusted R-squared) values that are greater than 0.9937 for all DESs under investigation. It is also confirmed by the fact that the average absolute residuals (AAD) between measured and predicted index values are in the order of 0.0001 to 0.0005, which is comparable to the accuracy of the refractometer.

**Figure 8:**
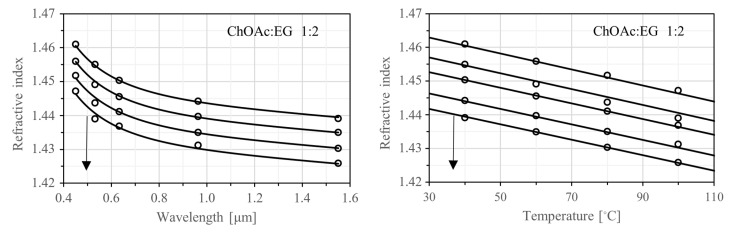
Refractive index as a function of wavelength (**left**) and temperature (**right**) for ChoAc:EG at a molar ratio 1:2. In the left plot, the arrow indicates the direction of increasing temperature (40 °C, 60 °C, 80 °C and 100 °C). In the right plot, the arrow indicates the direction of increasing wavelength (450 nm, 532 nm, 632.8 nm, 964 nm and 1551 nm). Open circles: Experimental data. Solid lines: Predicted values based on the proposed dispersion model.

Figure 8 shows two families of parametric curves for the DESs with the lowest fit quality—specifically, ChOAc:EG 1:2. The left plot illustrates how the refractive index varies with wavelength across the four measurement temperatures, while the right plot shows how it changes with temperature for the five measurement wavelengths. Similar plots for all DESs studied are provided in Figure S60 (Supporting Information).

In general, the refractive index decreases with increasing wavelength, a behavior known as normal dispersion. It also decreases with rising temperature, following an almost linear trend, which reflects the strong correlation between refractive index and density. The pronounced nonlinearity in the wavelength dependence indicates that Equation (7) should not be extrapolated beyond the spectral range of the measurements. In contrast, the near-linear temperature dependence suggests that the dispersion model may yield reasonable estimates even beyond the measured temperature range, including at room temperature. As a practical extension, estimated refractive index values at several standard spectroscopic wavelengths and a constant temperature of 25 °C are presented in Table 7.

Several observations can be made about the ordering of DESs based on their refractive index. First, there is a consistent trend showing that the refractive index increases when the molar ratio shifts from 1:3 to 1:2. Among the 1:3 molar ratio samples, the refractive index always increases in the following order: [ChOAc:EG] < [ChOAc:LevA] < [L-Pro:LevA] < [L-Carn:LevA] < [L-Carn:EG] < [ChOAc:Gly] < [L-Carn:Gly] < [L-Pro:Gly]. For the DESs with a 1:2 molar ratio, at lower temperatures (<80 °C) the order is: [ChOAc:EG] < [ChOAc:LevA] < [L-Carn:EG] < [ChOAc:Gly] < [L-Carn:LevA] < [L-Carn:Gly]. Notably, a reversal in the order of the first two components is observed at elevated temperatures.

From these observations, it can be concluded that across the entire temperature and wavelength range, DESs containing ChOAc as the HBA exhibit lower refractive indices than those with L-Pro or L-Carn, when mixed at the same molar ratio with the same HBD. Likewise, DESs containing Gly as the HBD consistently show higher refractive indices than those with LevA or EG, under identical mixing conditions with the same HBA. To conveniently summarize these trends, we introduce the notation $n_{x:y}^{\left(i:j\right)}$ to represent the refractive index of a DES composed of HBA x and HBD y at a molar ratio i:j. Using this notation, the observed relationships can be expressed as follows:

$$n_{x:y}^{\left(1:3\right)}<n_{x:y}^{\left(1:2\right)},$$

$$n_{ChOAc:y}^{\left(i:j\right)}<n_{EG:y}^{\left(i:j\right)}\;and\;n_{ChOAc:y}^{\left(i:j\right)}<n_{LevA:y}^{\left(i:j\right)},$$

$$n_{x:Gly}^{\left(i:j\right)}>n_{x:L-Pro}^{\left(i:j\right)}\;and\;n_{x:Gly}^{\left(i:j\right)}>n_{x:L-Carn}^{\left(i:j\right)}.$$

The ability to tune the refractive index of DESs by selecting appropriate HBA and HBD components, as well as adjusting their mixing ratios, makes them ideal candidates for applications requiring index matching. Such applications are common in fields such as microscopy, opto-fluidics, and tissue clearing, among others [59]. In this context, it is often more practical to use molar refractivity for design purposes, rather than the refractive index itself. This is because unlike the refractive index, molar refractivity (*R*_m_), defined below, is an additive material property.

(9) $$R_{m}=V_{m}\cdot \frac{n^{2}-1}{n^{2}+2}.$$

By substituting into Equation (9) the temperature-dependent molar volume $V_{m}$, as discussed in Section 3.2, along with the dispersion model for the refractive index $n\left(\lambda ,T\right)$, given in Equation (7), one can readily estimate the molar refractivity $R_{m}(\lambda ,T)$ as a function of both temperature and wavelength. Representative results from these calculations at two selected wavelengths and T=25 °C are presented in Table 8.

As expected, the ordering of the DESs based on their molar refractivity shows clearer and more consistent trends than those observed for the refractive index. Specifically, a systematic investigation reveals that, across all wavelengths and temperatures, molar refractivity increases when the molar ratio of a given HBA:HBD pair changes from 1:2 to 1:3. Additionally, molar refractivity increases in the order [L-Carn:EG] < [ChOAc:EG] < [L-Pro:Gly] < [L-Carn:Gly] < [ChOAc:Gly] < [L-Pro:LevA] < [L-Carn:LevA] < [ChOAc:LevA]. This trend holds for all eight available samples with a 1:3 molar ratio, as well as for the six samples with a 1:2 ratio. Introducing now the self-explanatory notation ${R_{m}}_{x:y}^{\left(i:j\right)}$, the following consistent relationships can be identified:

$${R_{m}}_{x:y}^{\left(1:3\right)}>{R_{m}}_{x:y}^{\left(1:2\right)},$$

$${R_{m}}_{ChOAc:y}^{\left(i:j\right)}>{R_{m}}_{L-Carn:y}^{\left(i:j\right)}>{R_{m}}_{L-Pro:y}^{\left(i:j\right)},$$

$${R_{m}}_{x:LevA}^{\left(i:j\right)}>{R_{m}}_{x:Gly}^{\left(i:j\right)}>{R_{m}}_{x:EG}^{\left(i:j\right)}.$$

Beyond molar refractivity, the dispersion model presented in Equation (7) can be used to calculate several other quantities that are highly relevant to both physical chemistry research and practical photonic applications. For instance, the derivative dn/dT, known as the thermo-optic coefficient, measures the refractive index response to temperature changes. Likewise, the derivatives dn/dλ and $d^{2}n/d\lambda^{2}$ capture first- and second-order chromatic dispersion effects, respectively. For example, the first derivative relates to the group velocity of spectrally broadband light pulses as they propagate through a dispersive medium, while the second derivative regulates the temporal broadening of those pulses. Representative calculations of dn/dT, dn/dλ and $d^{2}n/d\lambda^{2}$ at two selected wavelengths and T=25 °C are also presented in Table 8.

## 3. Materials and Methods

### 3.1. Material and Methods

Choline acetate 98% (ChOAc) was purchased from IOLITEC. L-Carnitine > 98% (L-carn) and L-Proline > 99% (L-Pro) were purchased from TCI. Ethylene glycol 99% (EG) and levulinic acid 98% (LevA) were purchased from Thermo Fisher, Waltham, MA, USA. Glycerol (Gly) 99% was obtained from Sigma-Aldrich (Merck, Darmstadt, Germany).

The ^1^H NMR analysis was performed at temperature of 25 °C with D_2_O as solvent using Bruker 400 MHz NMR instrument. (Bruker, Billerica, MA, USA) The chemical shifts (ppm) are referenced using D_2_O (δ_H_ 4.79) as internal standard. All samples were analyzed at the concentration of 25 mg/cm^3^.

A coulometer Karl Fischer (Titroline 75,000 KFtrace, SI Analytics GmbH, Mainz, Germany) was used for the determination of DESs water content.

The density data of DESs were collected using a U-shape densimeter (Anton Paar, Graz, Austria, DMA 4500 M) in the temperature range from 20 to 90 °C. For the calibration, the reference density values of water, obtained using the fundamental equation of state by Wagner and Pruss (uncertainty lower than ±0.003% in the full pressure and temperature ranges), were used.

DESs viscosities were measured using rheometer MCR 302, (Anton Paar, Graz, Austria), in the temperature range 20 to 90 °C using a plate–plate geometry (diameter of 5 cm). The flow curve was conducted at the shear rate spanning from 1 to 1000 s^−1^ at fixed temperature of 20 °C. For L-carn:Gly the measurements were conducted in the shear rate from 1 to 100 s^−1^. The effect of temperature was studied by measuring at a constant shear rate (50 s^−1^), considering that all mixtures behaved as Newtonian liquids. The temperature was regulated using Water-Cooled Peltier system (H-PTD200, Anton Paar).

The thermal stability of DESs and their constituents was investigated by thermal gravimetric analysis (TGA) conducted in a TA Instruments TGA550 (TA Instrument, New Castle, DE, USA) (weighing Precision ±0.01%, sensitivity 0.1 µg, base line dynamic drift < 50 µg). For the temperature calibration, Curie point of nickel standards was used. The mass calibration was performed using weight standards of 1000 mg, 500 mg, and 100 mg. The sample was subsequently heated from 40 °C to 500 °C at a rate of 10 °C/min in an inert nitrogen atmosphere (80 cm^3^/min), and then held at 500 °C for 2 min. The samples were analyzed in triplicate.

DESs thermal behavior was analyzed using differential TA DSC, Q250 (TA Instrument, New Castle, DE, USA), USA (enthalpy precision ± 0.08%, temperature precision ± 0.008 °C, temperature accuracy ± 0.05 °C). A mass of 2–5 mg of sample was charged in pin hole aluminum hermetic crucibles, and the temperature was varied with a scanning rate of 10 °C/min from −90 to 80/90 °C under nitrogen atmosphere (flow rate of 50 cm^3^/min). The temperature and enthalpy calibrations were performed using indium as reference material (melting point 156.6 °C and melting enthalpy Δ*H*_m_ = 28.71 J/g). DSC experiments were carried out in duplicate.

The refractive index was determined using a commercial prism-coupling refractometer (Metricon, Melbourne, Australia, model 2010/M), with a standard error of ±0.0002. The instrument features a Gadolinium Gallium Garnet reference prism of known refractive index, mounted on a computer-controlled rotary table. The sample is brought into contact with the base of the prism using a custom liquid holder, while a thermal resistor provides direct temperature control at the prism–sample interface. Illumination is provided by one of five monochromatic laser sources (450 nm, 532 nm, 632.8 nm, 964 nm, and 1551 nm). By measuring specular reflectance across varying incidence angles, the onset of total internal reflection (TIR) is identified, from which the refractive index is calculated using the standard TIR condition.

### 3.2. DES Preparation

The hydrogen bond acceptor (ChOAc, L-Carn or L-Pro) and the proper hydrogen bond donor (EG, Gly or LevA) were mixed under magnetic stirring in different molar ratios (1:2 or 1:3) from room temperature to 60 °C (the specific temperature adopted for each DES is reported in Table 1) until a homogenous transparent liquid was formed. No further purification was needed after the DESs preparation, while an additional drying step was performed at 40 °C under vacuum, in order to remove the moisture absorbed during the DESs formation. The DESs composition and purity was assessed by ^1^H-NMR in D_2_O, and the spectra were reported in Supplementary File (Figures S1–S8).

## 4. Conclusions

The physico-chemical properties of a set of DESs based on choline acetate, L-carnitine and L-proline as HBA and ethylene glycol, glycerol and levulinic acid as HBD, in different molar ratios, have been experimentally studied and compared at various temperatures.

The density values, as well as the thermal expansion coefficient (*β*) of the considered DESs was found to be heavily influenced by the nature of both HBA and HBD. ChOAc was found to be the HBA yielding the lowest density DESs, while L-carn-based DESs exhibited the lowest thermal expansion coefficients, and L-Pro-based DESs were found to show both the highest densities and the highest *β* values. On the other hand, concerning the HBDs, EG-based systems displayed the lowest density in the study, while Gly-based ones displayed the highest. No clear density trend was observable regarding the HBA:HBD ratio, with its effect on the density appearing strictly depending on the nature of both the HBA and the HBD. Conversely, the effect of the ratio on the *β* appeared much more evident, with the values of *β* growing passing from the 1:2 to the 1:3 molar ratio.

Similarly, HBA, HBD and molar ratio were found to be crucial parameters also for the definition of the viscosity of the considered DESs. Parallelly to what observed about density, EG-based DESs displayed the lowest viscosities while Gly-based were found to be the most viscous ones. Concerning the effect of the HBA, ChOAc systems displayed the lowest viscosity, while L-carn-based ones exhibited the highest viscosity values. Finally, DESs with 1:3 HBA:HBD molar ratios manifested lower viscosities compared to their 1:2 ratio counterparts. The decrease in viscosity as a function of temperature was found to be best fitted by the VFT model. The number of functional groups capable of effectively participating in hydrogen bonding, directly acting on the strength of the hydrogen bonds occurring between the DESs components, was found to play a preeminent role both in the definition of the density and of the viscosity characteristics of the investigated DESs.

The thermal stability properties of the investigated DESs highlighted a decisive role of the HBD, with EG-based systems displaying the lowest stabilities and Gly-based systems displaying the highest ones. On the other hand, the role of the HBA was found to be difficultly quantifiable. Surprisingly, the HBA:HBD molar ratio was found to play an almost negligible role on the thermal stability of the studied DESs, differently from what was previously observed by our research group about a different set of DESs based on ChCl and Bet, where this parameter appeared to be decisive in the definition of the degradation profile [40]. Noteworthy differences were generally appreciable between the degradation temperatures of Gly- and LevA-based DESs and their constituting HBDs which, together with their single-step degradation profile, suggest the presence of uncommonly strong interactions among the constituents of the investigated DESs. Finally, all the considered samples displayed glass transitions as the only observable thermal phenomenon in the analyzed temperature range, with the exception of EG-based systems which showed none. Gly-based DESs displayed lower *T*_g_ values than their LevA counterparts, while L-carn-based DESs manifested higher *T*_g_ values than the corresponding ChOAc-based DESs. Regarding the effect of the DES composition on the refractive index, an increase in this quantity was observed passing from 1:3 to 1:2 systems, with the DESs containing ChOAc as the HBA exhibiting lower refractive indices than those with L-Pro or L-Carn, and the DESs containing Gly as the HBD consistently showing higher refractive indices than those with LevA or EG. In general, the refractive index was found to decrease with increasing wavelength, following a general behavior known as normal dispersion, and to almost linearly decrease with rising temperature, reflecting the strong correlation between refractive index and density. On the other hand, molar refractivity was observed to increase passing from 1:2 to 1:3 molar ratio. Concerning the effects of HBA and HBDs, ChOAc-based and LevA-based DESs were found to exhibit the highest molar refractivity, while L-Pro-based and EG-based DESs were found to display the lowest molar refractivities.

A full chemical and physical characterization of these DES enables us to evaluate the impact of altering the HBA, HBD and their ratio. This is a primary consideration for identifying the field of application of these DES classes. Choline acetate-based DES can be used in any application requiring a basic DES. Conversely, carnitine- and pro-line-based DES could be used in applications where chirality plays a primary role.

## Figures and Tables

**Table 1:** Summary of the DESs studied in this work.

DES	HBA	HBD	Molar Ratio	Temperature Preparation
ChOAc:EG	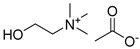	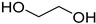	1:2	r.t.
			1:3	r.t.
ChOAc:Gly		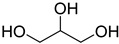	1:2	r.t.
			1:3	r.t.
ChOAc:LevA		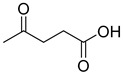	1:2	r.t
			1:3	r.t.
L-Carn:EG	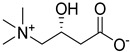	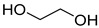	1:2	40
			1:3	40
L-Carn:Gly		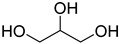	1:2	60
			1:3	60
L-Carn:LevA		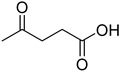	1:2	40
			1:3	40
L-Pro:Gly	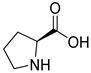	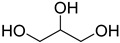	1:3	40
L-Pro:LevA		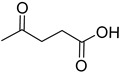	1:3	r.t.

**Table 2:** Optimal parameters of the linear model *ρ* = *A*T + *B* (*A*: slope of the linear dependence between density and temperature, *B*: the density at 0 °C) for the temperature dependence of density from 20 °C to 90 °C, for the indicated DESs and molar ratios. *β* is thermal expansion coefficient which expresses the ease with which a liquid expands when heated under constant pressure.

DES	Molar Ratio	R^2^	*A* [10^−4^ g·cm^−3^·°C^−1^]	*B* [g/cm^3^]	*β* [10^−3^ °C^−1^] at 25 °C
ChOAc:EG	1:2	0.99999	5.77286	1.11389	0.52506
ChOAc:EG	1:3	1.00000	5.95329	1.11681	0.54026
L-Carn:EG	1:2	0.99999	6.09371	1.17896	0.52364
L-Carn:EG	1:3	0.99998	6.10286	1.17814	0.52480
ChOAc:Gly	1:2	1.00000	5.65886	1.18341	0.48397
ChOAc:Gly	1:3	1.00000	5.76371	1.19956	0.48633
L-Carn:Gly	1:2	1.00000	5.72607	1.2534	0.46212
L-Carn:Gly	1:3	0.99997	5.9364	1.25097	0.48024
L-Pro:Gly	1:3	0.99993	6.21064	1.28311	0.48996
ChOAc:LevA	1:2	0.99995	6.84621	1.13821	0.61067
ChOAc:LevA	1:3	0.99992	7.12086	1.14266	0.63305
L-Carn:LevA	1:2	0.99995	6.64493	1.19784	0.56254
L-Carn:LevA	1:3	0.99996	7.03964	1.18959	0.60066
L-Pro:LevA	1:3	0.99997	7.61121	1.20287	0.64292

**Table 3:** Arrhenius model’s optimized fitting parameters for the logarithm of viscosity vs. 1 by temperature plots in the 20–90 °C range, for the indicated DES and molar ratio.

DES	Molar Ratio	R^2^	ln η_∞_	η_∞_	*E*_a_ [KJ/mol]
ChOAc:EG	1:2	0.99438	−6.29257	0.00185	26.20305
ChOAc:EG	1:3	0.99468	−6.0576	0.00234	24.54061
ChOAc:Gly	1:2	0.99477	−10.1875	3.7638 × 10^−5^	41.17333
ChOAc:Gly	1:3	0.9937	−10.7526	2.13903 × 10^−5^	42.24729
ChOAc:LevA	1:2	0.98649	−6.97395	9.35944 × 10^−4^	29.09286
ChOAc:LevA	1:3	0.9869	−7.21686	7.34107 × 10^−4^	29.32486
L-Carn:EG	1:2	0.99015	−8.89192	1.37496 × 10^−4^	37.27208
L-Carn:EG	1:3	0.99326	−8.88613	1.38294 × 10^−4^	36.85980
L-Carn:Gly	1:2	0.99524	−15.279	2.31437 × 10^−7^	61.18449
L-Carn:Gly	1:3	0.99475	−13.9439	8.79538 × 10^−7^	55.48258
L-Carn:LevA	1:2	0.99003	−14.2988	6.16775 × 10^−7^	55.84304
L-Carn:LevA	1:3	0.98915	−11.945	6.49152 × 10^−6^	46.43079
L-Pro:Gly	1:3	0.99386	−14.1323	7.28515 × 10^−7^	55.15447
L-Pro:LevA	1:3	0.99001	−9.43662	7.97495 × 10^−5^	37.81611

**Table 4:** VFT model’s optimized parameters obtained from the fitting of the measured viscosities in the 20–90 °C range, according to Equation (6), for the indicated DES and molar ratio.

DES	Molar Ratio	R^2^	η_∞_ [mPa×s]	*B* [K]	*T*_0_ [K]
ChOAc:EG	1:2	0.99994	0.29394	707.30503	170.65838
ChOAc:EG	1:3	0.99994	0.27966	652.45637	171.66885
ChOAc:Gly	1:2	0.99991	0.05685	1301.69054	159.04581
ChOAc:Gly	1:3	0.99993	0.05791	1206.76267	167.20874
ChOAc:LevA	1:2	0.99969	0.56689	554.82436	195.47049
ChOAc:LevA	1:3	0.99969	0.49431	546.042	196.87856
L-Carn:EG	1:2	0.99988	0.20562	956.315	175.6274
L-Carn:EG	1:3	0.99993	0.15470	1017.7194	169.6058
L-Carn:Gly	1:2	0.99988	0.04243	1607.13449	171.16122
L-Carn:Gly	1:3	0.99989	0.02827	1618.65234	164.30309
L-Carn:LevA	1:2	0.99993	0.03778	1381.88331	179.39328
L-Carn:LevA	1:3	0.99990	0.10953	1003.98127	187.93651
L-Pro:Gly	1:3	0.99992	0.01410	1716.50159	160.57514
L-Pro:LevA	1:3	0.99994	0.70274	553.98967	209.63984

**Table 5:** TGA of the investigated DESs. *T*_5%_ indicates the temperature at which a 5% loss of mass recorded; *Tonset* indicates the onset of the Weight% vs. Temperature plot; *T*_peak_ indicates the main degradation peak observed in the *d*Weight%/*d*Temperature vs. Temperature plot. *T*_g_ was obtained by taking the midpoint of the heat capacity change on heating from a glass to a liquid.

	TGA			DSC
DES	*T*_5%_ (°C)	*T*_onset_ (°C)	*T*_peak_ (°C)	*T*_g_ (°C)
ChOAc:EG 1:2	96.85	94.99	130.45–213.02	-
ChOAc:EG 1:3	90.17	93.86	127.85–213.10	-
ChOAc:Gly 1:2	155.88	192.81	225.79	−86.78
ChOAc:Gly 1:3	161.23	197.80	233.94	−86.40
ChOAc:Lev 1:2	116.62	197.57	131.78–228.09	−82.14
ChOAc:LevA 1:3	119.55	194.48	229.58	−81.55
L-Carn:EG 1:2	99.32	96.64	127.02–181.11	-
L-Carn:EG 1:3	96.08	98.45	130.50–176.85	-
L-Carn:Gly 1:2	161.96	180.26	208.26–398.94	−62.80
L-Carn:Gly 1:3	157.65	181.85	207.56–392.56	−72.03
L-Carn:LevA 1:2	150.70	183.15	210.49	−46.88
L-Carn:LevA 1:3	136.82	173.66	206.25	−59.50
L-Pro:Gly 1:3	161.62	194.20	234.62	−73.33
L-Pro:LevA 1:3	132.50	141.32	164.65–204.71–422.07	−66.82

**Table 6:** Fitting coefficients of the dispersion model of Equation (7) for the investigated DESs.

DES	Molar Ratio	s_1_	s_2_ (C^−1^)	d (μm^−2^)	λ_uv_ (μm)	R^2^/AdjR^2^	AAD
ChOAc:EG	1:2	1.102967487	−0.000653575	−0.003844485	0.100948921	>0.9937	0.0005
ChOAc:EG	1:3	1.091076617	−0.000684437	−0.003792241	0.100701826	>0.9966	0.0004
ChOAc:Gly	1:2	1.153146963	−0.000660690	−0.004829819	0.100917707	>0.9995	0.0001
ChOAc:Gly	1:3	1.152632669	−0.000675674	−0.004962575	0.100591623	>0.9994	0.0002
ChOAc:LevA	1:2	1.113103252	−0.000840514	−0.003150331	0.105039717	>0.9983	0.0003
ChOAc:LevA	1:3	1.107694963	−0.000872402	−0.002882189	0.105927355	>0.9988	0.0003
L-Carn:EG	1:2	1.149387691	−0.000705678	−0.003954361	0.103370805	>0.9995	0.0002
L-Carn:EG	1:3	1.149188941	−0.000700173	−0.004912343	0.100767349	>0.9991	0.0002
L-Carn:Gly	1:2	1.21641889	−0.000686390	−0.005962162	0.100130031	>0.9977	0.0004
L-Carn:Gly	1:3	1.193374374	−0.000671018	−0.004493721	0.099551282	>0.9954	0.0005
L-Carn:LevA	1:2	1.163412686	−0.000775182	−0.003683183	0.105227090	>0.9994	0.0002
L-Carn:LevA	1:3	1.141971229	−0.000821296	−0.003604593	0.105216084	>0.9991	0.0002
L-Pro:Gly	1:3	1.206243260	−0.000719176	−0.005814156	0.100979329	>0.9990	0.0002
L-Pro:LevA	1:3	1.139327897	−0.000916420	−0.003884250	0.106096480	>0.9994	0.0002

**Table 7:** Predicted refractive index values at a temperature of 25°C and different wavelengths, corresponding to standard spectroscopic lines.

DES	Molar Ratio	Wavelength (nm)							
		404.7	480.0	546.1	587.6	589.3	656.3	852.1	1014.0
ChOAc:EG	1:2	1.4690	1.4615	1.4574	1.4555	1.4554	1.4530	1.4489	1.4469
ChOAc:EG	1:3	1.4643	1.4569	1.4528	1.4509	1.4508	1.4485	1.4444	1.4425
ChOAc:Gly	1:2	1.4870	1.4792	1.4749	1.4729	1.4729	1.4704	1.4660	1.4639
ChOAc:Gly	1:3	1.4865	1.4788	1.4745	1.4725	1.4724	1.4700	1.4657	1.4635
ChOAc:LevA	1:2	1.4732	1.4650	1.4605	1.4584	1.4584	1.4558	1.4514	1.4494
ChOAc:LevA	1:3	1.4715	1.4632	1.4586	1.4565	1.4564	1.4539	1.4494	1.4474
L-Carn:EG	1:2	1.4866	1.4785	1.4740	1.4719	1.4718	1.4693	1.4648	1.4627
L-Carn:EG	1:3	1.4852	1.4774	1.4731	1.4711	1.4711	1.4686	1.4643	1.4622
L-Carn:Gly	1:2	1.5088	1.5008	1.4964	1.4943	1.4943	1.4917	1.4872	1.4849
L-Carn:Gly	1:3	1.5005	1.4928	1.4885	1.4865	1.4865	1.484	1.4797	1.4776
L-Carn:LevA	1:2	1.4921	1.4836	1.4789	1.4767	1.4766	1.4740	1.4694	1.4672
L-Carn:LevA	1:3	1.4840	1.4755	1.4709	1.4688	1.4687	1.4661	1.4616	1.4594
L-Pro:Gly	1:3	1.5054	1.4973	1.4928	1.4908	1.4907	1.4881	1.4836	1.4813
L-Pro:LevA	1:3	1.4826	1.4741	1.4694	1.4672	1.4671	1.4645	1.4599	1.4577

**Table 8:** Predicted values of molar refractivity, thermos-optic coefficient, first and second wavelength dispersion derivatives at a temperature of 25 °C and two different wavelengths, on in the visible and one in the near-infrared.

DES	Molar Ratio	Wavelength (nm)							
		*R*_m_ (cm^3^/mol)		dn/dT·10^4^ (C^−1^)		dn/dλ (μm^−1^)		d^2^n/dλ^2^ (μm^−2^)	
		0.5 μm	1 μm	0.5 μm	1 μm	0.5 μm	1 μm	0.5 μm	1 μm
ChOAc:EG	1:2	71.6	69.8	−2.3	−2.3	−0.0673	−0.0105	0.4123	0.0210
ChOAc:EG	1:3	86.1	84.0	−2.5	−2.4	−0.0663	−0.0103	0.4065	0.0207
ChOAc:Gly	1:2	84.1	82.0	−2.3	−2.3	−0.0697	−0.0114	0.4252	0.0212
ChOAc:Gly	1:3	104.8	102.3	−2.4	−2.3	−0.0693	−0.0114	0.4218	0.0209
ChOAc:LevA	1:2	97.2	94.7	−3.0	−2.9	−0.0732	−0.0107	0.4534	0.0236
ChOAc:LevA	1:3	124.9	121.6	−3.1	−3.0	−0.0741	−0.0106	0.4604	0.0242
L-Carn:EG	1:2	69.3	67.5	−2.5	−2.4	−0.0728	−0.0111	0.4482	0.0229
L-Carn:EG	1:3	84.2	82.1	−2.5	−2.4	−0.0693	−0.0114	0.4224	0.0209
L-Carn:Gly	1:2	81.9	79.9	−2.4	−2.3	−0.0716	−0.0123	0.4337	0.021
L-Carn:Gly	1:3	102.6	100.1	−2.3	−2.3	−0.0693	−0.0111	0.4231	0.0213
L-Carn:LevA	1:2	94.9	92.5	−2.7	−2.7	−0.0761	−0.0113	0.4706	0.0243
L-Carn:LevA	1:3	122.2	119.0	−2.9	−2.8	−0.0750	−0.0112	0.4637	0.0239
L-Pro:Gly	1:3	90.2	88.0	−2.5	−2.5	−0.0724	−0.0123	0.4392	0.0214
L-Pro:LevA	1:3	109.7	106.8	−3.3	−3.2	−0.0762	−0.0115	0.4709	0.0241

## Data Availability

Data are contained within the article or the Supplementary Materials.

## Supplementary Materials

The following supporting information can be downloaded at https://www.mdpi.com/article/10.3390/ijms26178625/s1.
