# Supplementary material for: Choline Acetate-, L-Carnitine- and L-Proline-Based Deep Eutectic Solvents: A Comparison of Their Physicochemical and Thermal Properties in Relation to the Nature and Molar Ratios of HBAs and HBDs
Source: Int J Mol Sci. 2025 Sep 4;26(17):8625. doi: 10.3390/ijms26178625 (PMC12429102; doi:10.3390/ijms26178625)
Supplement: Supplementary file 1 [file ijms-26-08625-s001.zip › ijms-3825219-supplementary.pdf]

Choline acetate, L-carnitine and L-Proline-based deep eutectic solvents: a comparison of their physicochemical and thermal properties in relationship with the nature and molar ratios of HBAs and HBDs

Luca Guglielmero [a,b,#], Angelica Mero [a,#], Spyridon Koutsoumpos[c], Sotiria Kripotou [c], Konstantinos Moutzouris [c], Lorenzo Guazzelli [a], Andrea Mezzetta [a,*]

[a] Università di Pisa, Dipartimento di Farmacia, via Bonanno 33, 56126 Pisa, Italy.

[b] Scuola Normale Superiore, Classe di Scienze, Piazza dei Cavalieri 7, 56126 Pisa, Italy.

[c] Laboratory of Electronic Devices and Materials, Department of Electrical and Electronic Engineering, University of West Attica, Egaleo 12244, Greece

*corresponding author: andrea.mezzetta@unipi.it

[#]equal contributions

Supporting Information

Table of contents

^{1}H NMR spectra of choline acetate, L-carnitine and L-proline based-DESs 2

Tables of density values of choline acetate, L-carnitine and L-proline based-DESs 6

Fitting of density data of choline acetate, L-carnitine and L-proline based-DESs 9

Tables of molar volume values of choline acetate, L-carnitine and L-proline based-DESs 13

Tables of viscosity values of choline acetate, L-carnitine and L-proline based-DESs 16

Fitting of viscosity data of choline acetate, L-carnitine and L-proline based-DESs 19

TG analysis of choline acetate, L-carnitine and L-proline based-DESs 24

DSC of choline acetate, L-carnitine and L-proline based-DESs 29

Experimental and predicted refractive index values for all DESs studied 34

Fitting of refractive index data for all DESs studied 35

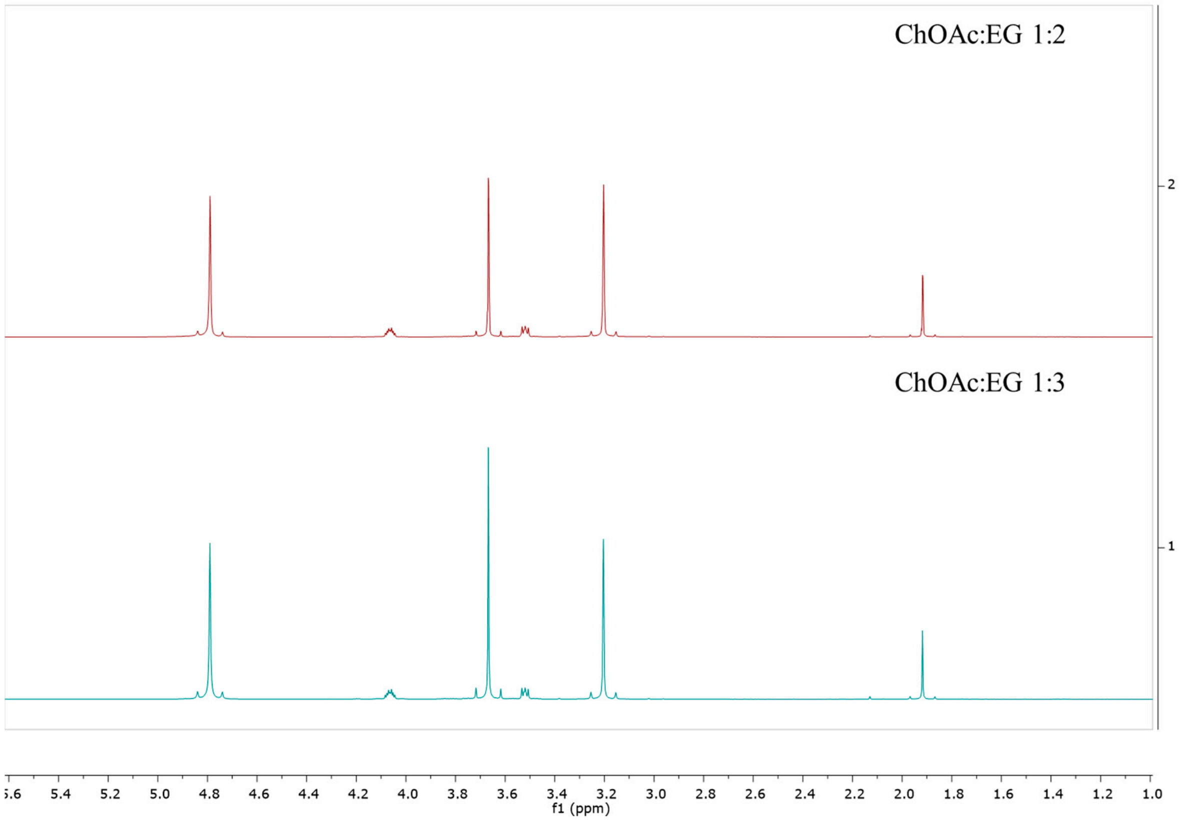

Figure S1. ^{1}H-NMR of DESs based on choline acetate (ChOAc) and ethylen glycole (EG).

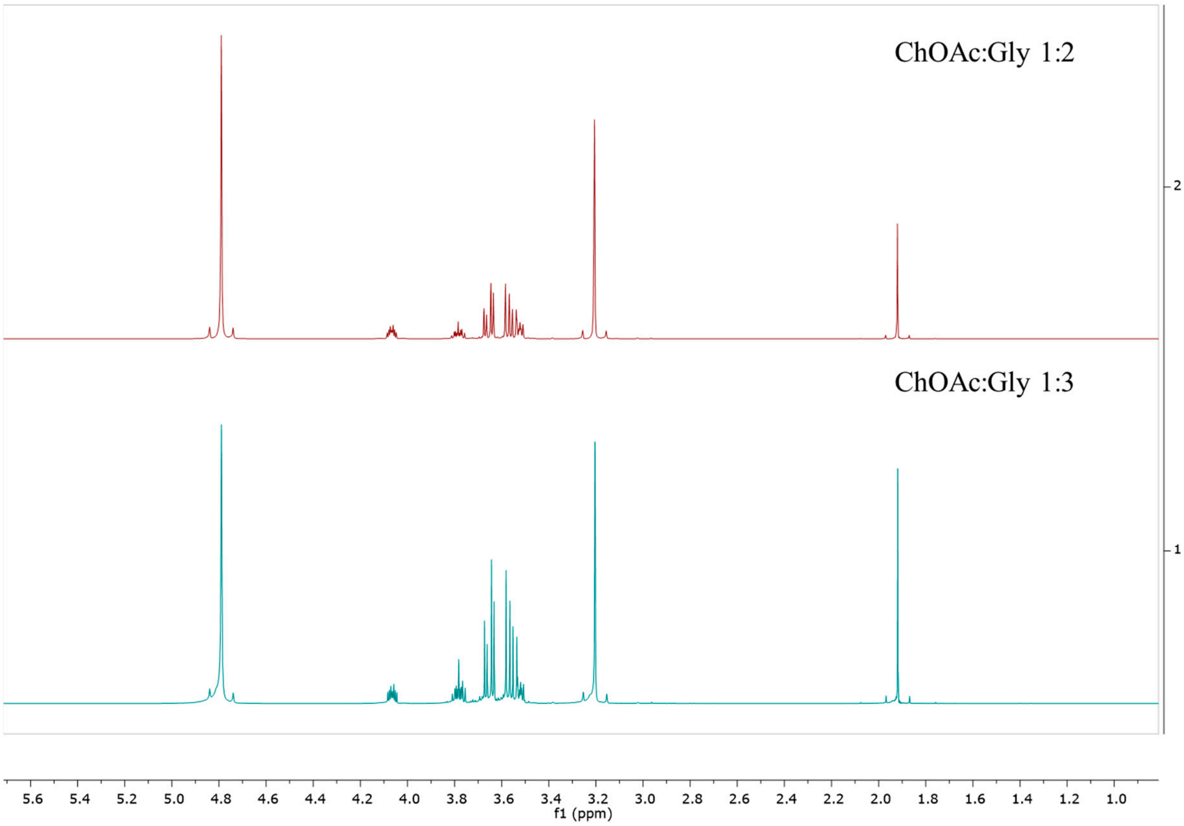

Figure S2. ^{1}H-NMR of DESs based on choline acetate (ChOAc) and glycerol (Gly).

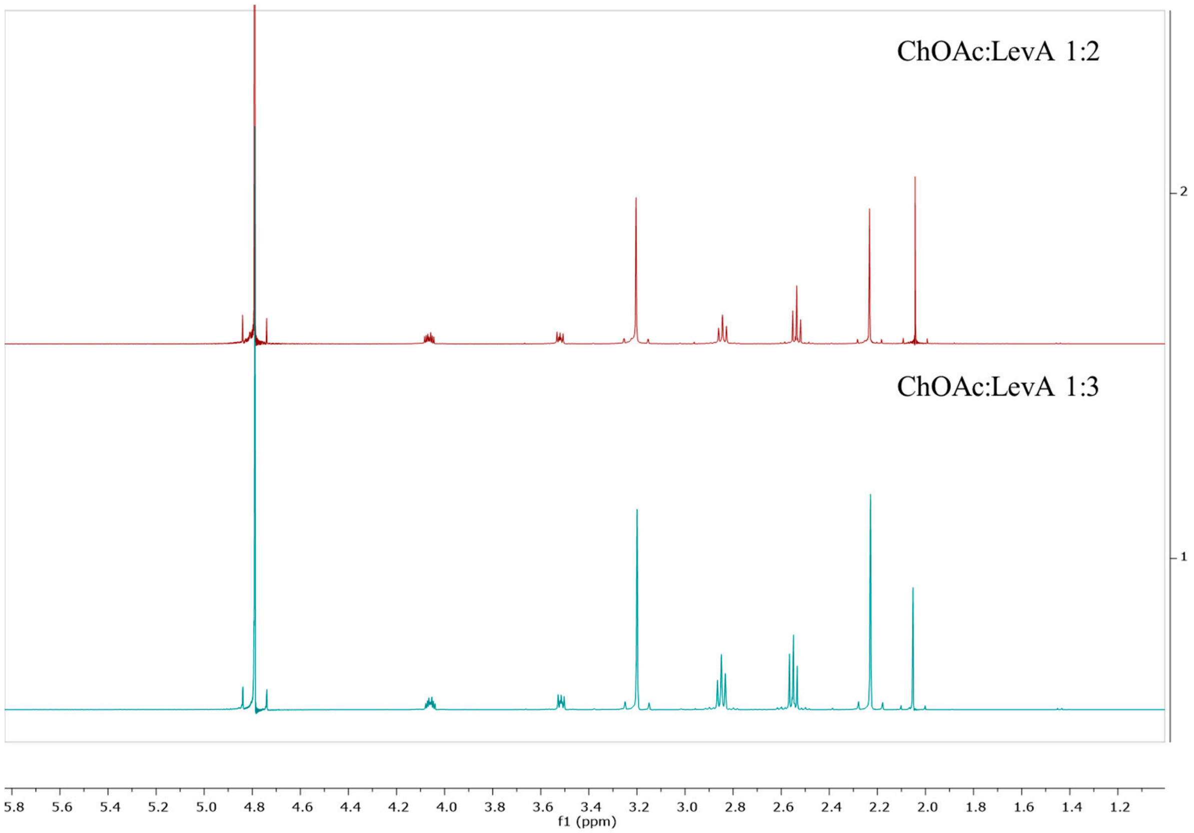

Figure S3. ^{1}H-NMR of DESs based on choline acetate (ChOAc) and levulinic acid (LevA).

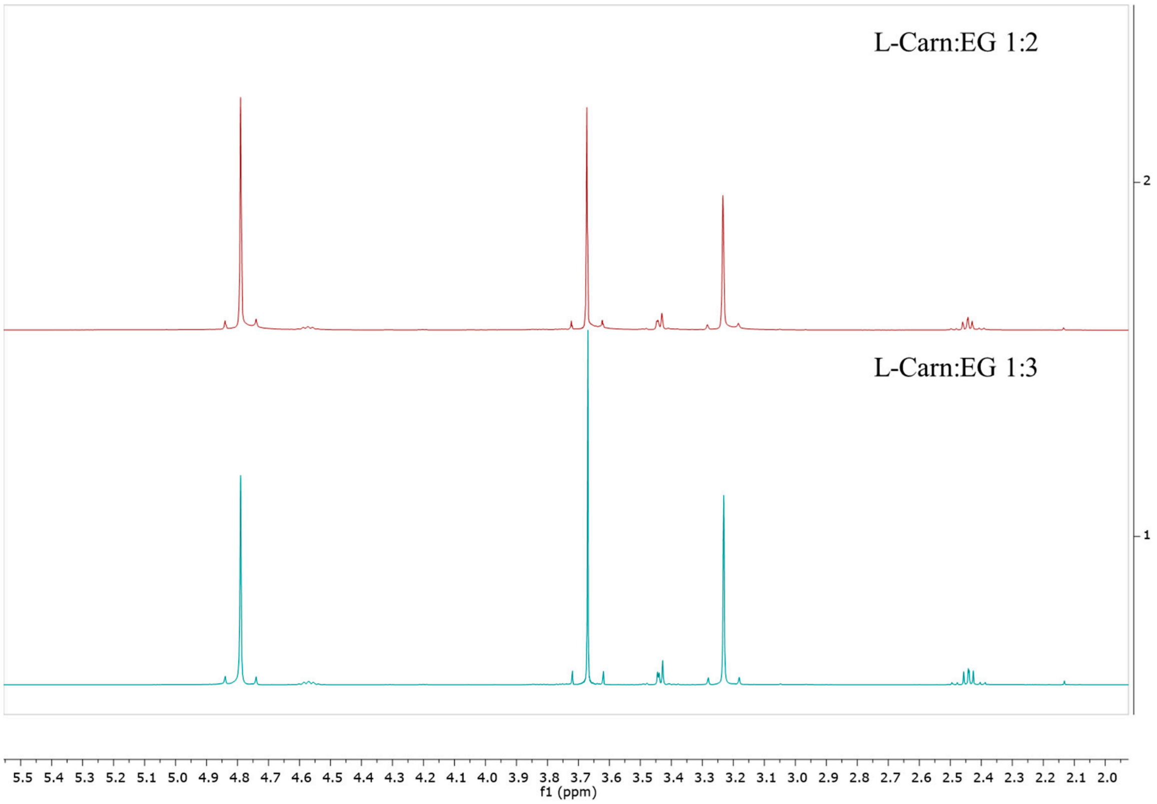

Figure S4. ^{1}H-NMR of DESs based on L-carnitine (L-carn) and ethylen glycol (EG).

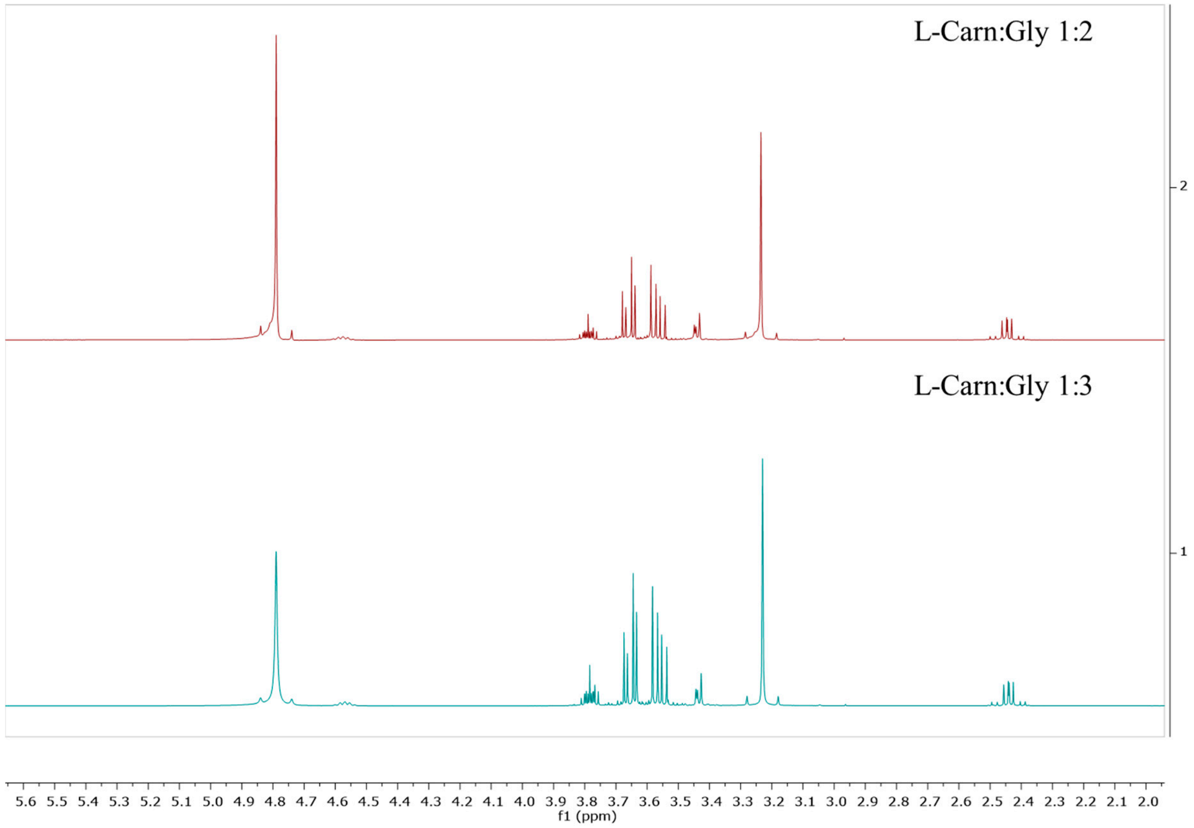

Figure S5. ^{1}H-NMR of DESs based on L-carnitine (L-carn) and glycerol (Gly).

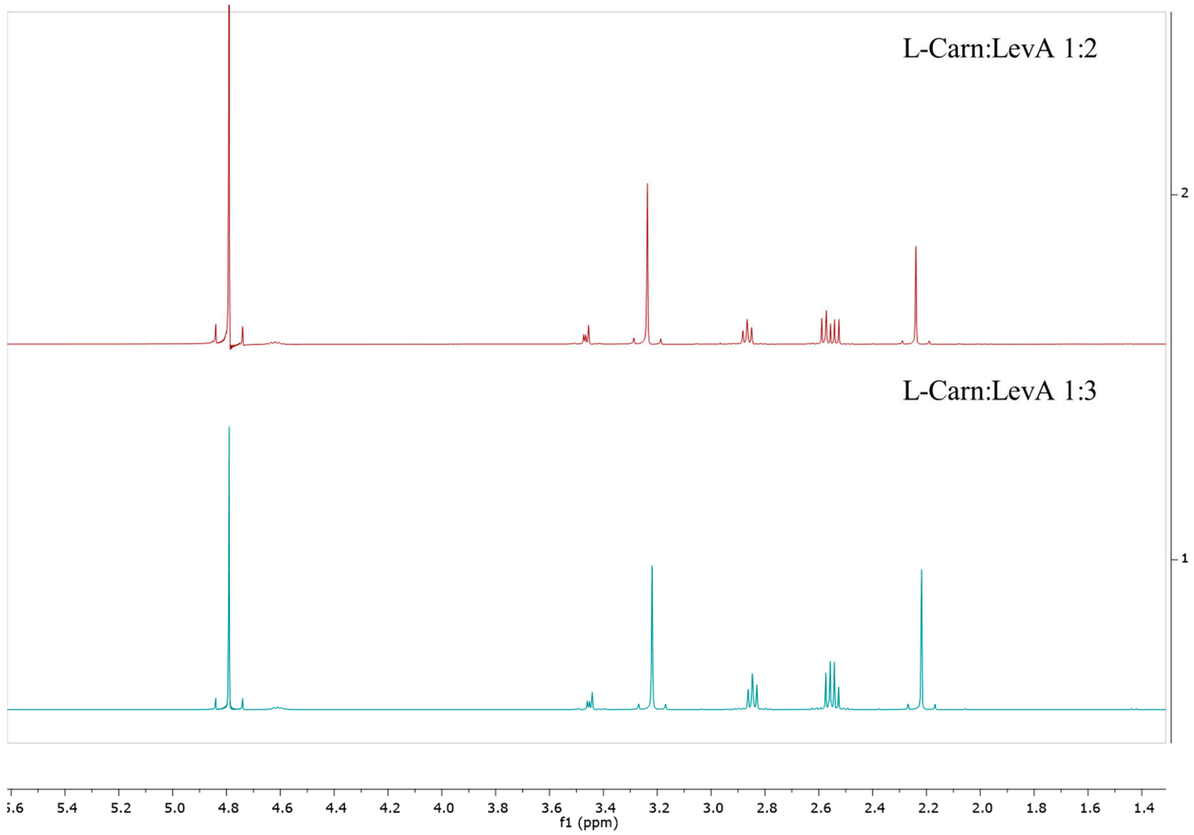

Figure S6. ^{1}H-NMR of DESs based on L-carnitine (L-carn) and levulinic acid (LevA).

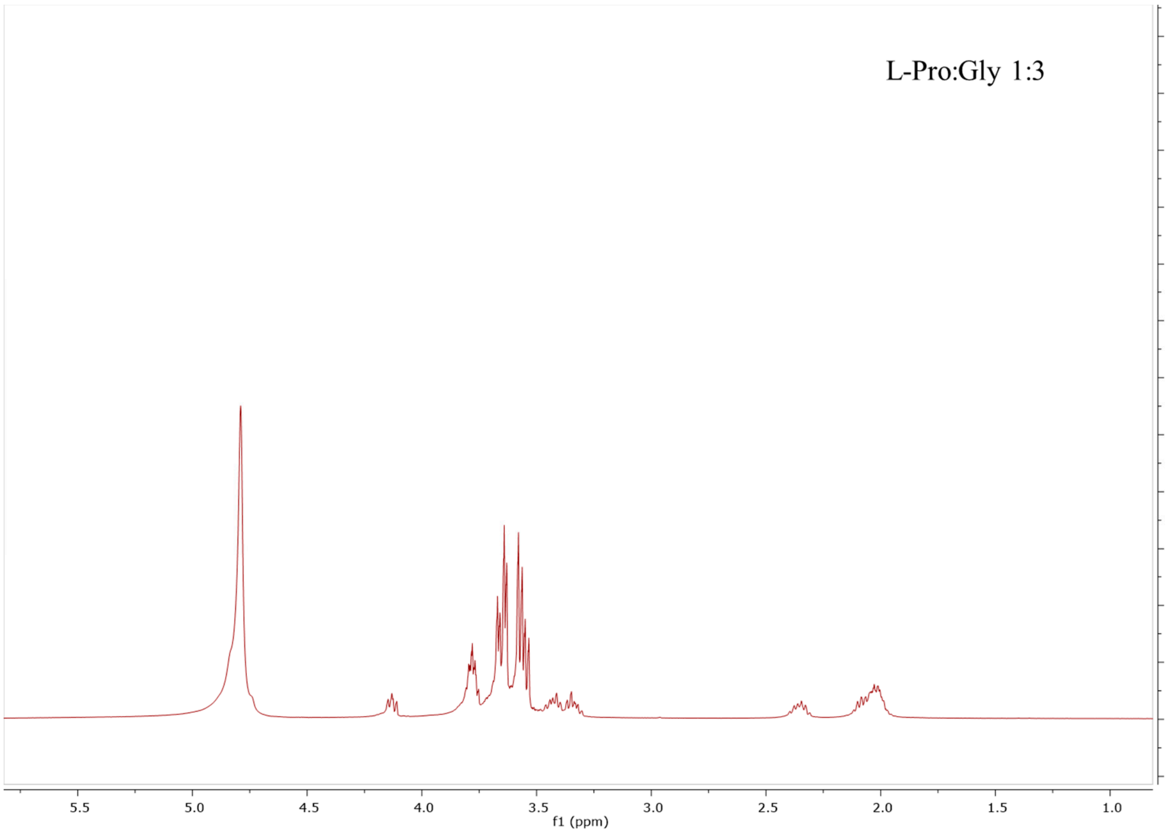

Figure S7. ^{1}H-NMR of DESs based on L-proline (L-Pro) and glycerol (Gly).

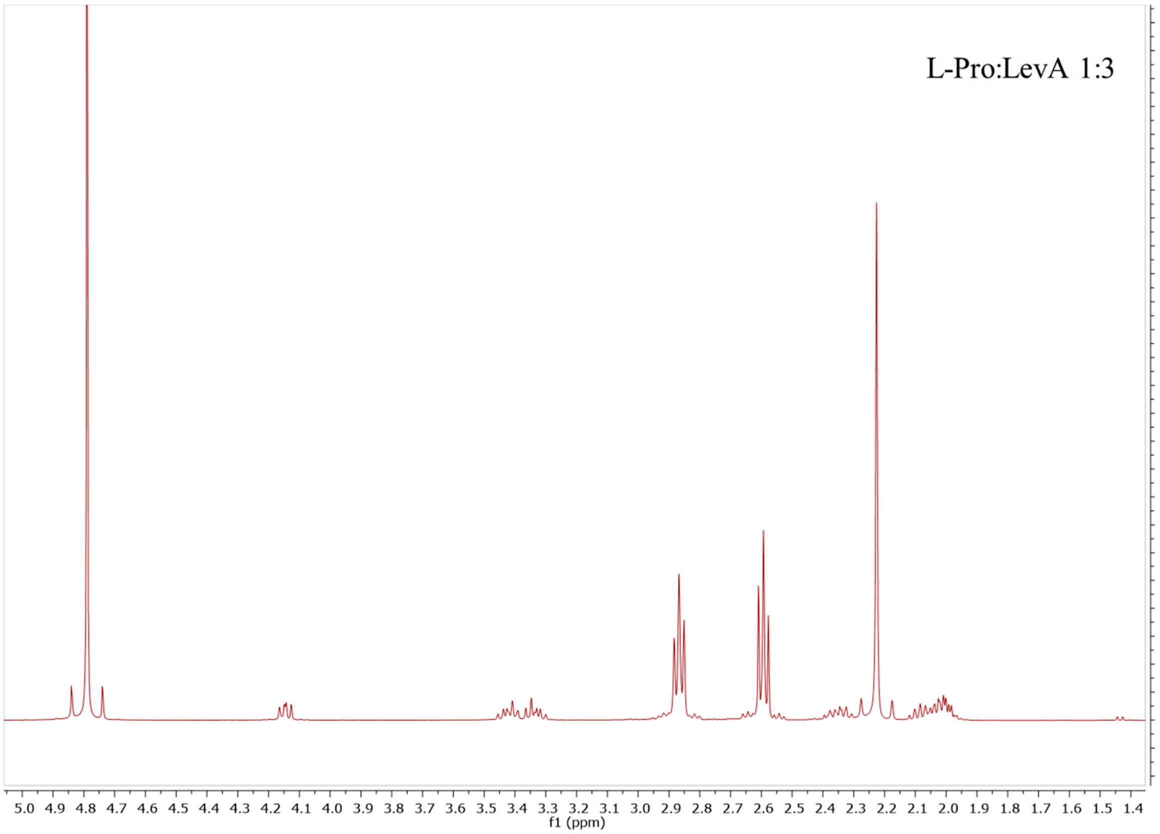

Figure S8. ^{1}H-NMR of DESs based on L-proline (L-Pro) and levulinic acid (LevA).

Table S1. Density data of ethylene glycol-based DESs.

	Density ρ (g/cm^3)				
**T (°C)**	**EG**	**ChOAc:EG 1:2**	**ChOAc:EG 1:3**	**L-Carn:EG 1:2**	**L-Carn:EG 1:3**
**20**	1.11147	1.10244	1.10494	1.16685	1.16677
**25**	1.10798	1.0995	1.10193	1.16377	1.16377
**30**	1.10448	1.09658	1.09894	1.1607	1.1607
**35**	1.10096	1.09367	1.09596	1.15762	1.15762
**40**	1.09743	1.09077	1.09298	1.15455	1.15455
**45**	1.09387	1.08788	1.09001	1.15149	1.15149
**50**	1.09029	1.08499	1.08704	1.14845	1.14845
**55**	1.08668	1.0821	1.08407	1.14541	1.14541
**60**	1.08305	1.07922	1.0811	1.14237	1.14237
**65**	1.07939	1.07634	1.07813	1.13934	1.13934
**70**	1.0757	1.07347	1.07515	1.13631	1.13631
**75**	1.07197	1.0706	1.07217	1.13327	1.13327
**80**	1.06821	1.06773	1.06919	1.13024	1.13024
**85**	1.06441	1.06486	1.06621	1.1272	1.1272
**90**	1.06058	1.062	1.06322	1.12415	1.12415

Table S2. Density data of glycerol-based DESs.

	Density ρ (g/cm³)					
**T (°C)**	**Gly**	**ChOAc:Gly 1:2**	**ChOAc:Gly 1:3**	**L-Carn:Gly 1:2**	**L-Carn:Gly 1:3**	**L-Pro:Gly 1:3**
**20**	1.25956	1.17207	1.188	1.24195	1.23897	1.27048
**25**	1.25647	1.16925	1.18514	1.23909	1.23606	1.26746
**30**	1.25335	1.16643	1.18227	1.23622	1.23314	1.26443
**35**	1.25019	1.16361	1.17939	1.23335	1.23021	1.26139
**40**	1.24702	1.16078	1.17652	1.23049	1.22728	1.25833
**45**	1.24382	1.15796	1.17364	1.22763	1.22433	1.25526
**50**	1.24058	1.15512	1.17076	1.22477	1.22137	1.25218
**55**	1.23731	1.15229	1.16787	1.22191	1.2184	1.24908
**60**	1.23403	1.14946	1.16499	1.21905	1.21542	1.24596
**65**	1.23073	1.14663	1.16212	1.21618	1.21243	1.24283
**70**	1.22739	1.1438	1.15924	1.21332	1.20944	1.23964
**75**	1.22403	1.14097	1.15635	1.21045	1.20645	1.23654
**80**	1.22064	1.13814	1.15346	1.20758	1.20346	1.23339
**85**	1.21722	1.1353	1.15055	1.20473	1.20047	1.23023
**90**	1.21376	1.13246	1.14764	1.20187	1.19742	1.227

Table S3. Density data of levulinic acid (LevA)-based DESs.

				Density ρ (g/cm^3)			
**T (°C)**	**LevA**	**ChOAc:LevA 1:2**	**ChOAc:LevA 1:3**	**L-Carn:LevA 1:2**	**L-Carn:LevA 1:3**	**L-Pro:LevA 1:2**	**L-Pro:LevA 1:3**
**20**	1.13983	1.12475	1.1287	1.18474	1.17568	--	1.18783
**25**	1.13552	1.12121	1.125	1.18133	1.17209	--	1.18394
**30**	1.13122	1.1177	1.12132	1.17793	1.16851	--	1.18006
**35**	1.12693	1.11421	1.11768	1.17456	1.16494	1.19332	1.1762
**40**	1.12264	1.11074	1.11406	1.1712	1.16138	1.18239	1.17236
**45**	1.11836	1.10729	1.11048	1.16785	1.15783	1.17818	1.16852
**50**	1.11408	1.10386	1.10691	1.16451	1.1543	1.17447	1.1647
**55**	1.10979	1.10045	1.10336	1.16118	1.15077	1.17081	1.1609
**60**	1.10551	1.09704	1.09981	1.15787	1.14726	1.16721	1.15711
**65**	1.10124	1.09364	1.09628	1.15457	1.14375	1.16368	1.15333
**70**	1.09696	1.09024	1.09275	1.15128	1.14027	1.16019	1.14956
**75**	1.09270	1.08686	1.08923	1.14799	1.13679	1.15671	1.14578
**80**	1.08843	1.08349	1.08573	1.14472	1.13332	1.15321	1.14202
**85**	1.08415	1.08012	1.08226	1.14146	1.12985	1.14963	1.13826
**90**	1.07987	1.07677	1.0788	1.1382	1.12639	1.14601	1.13451

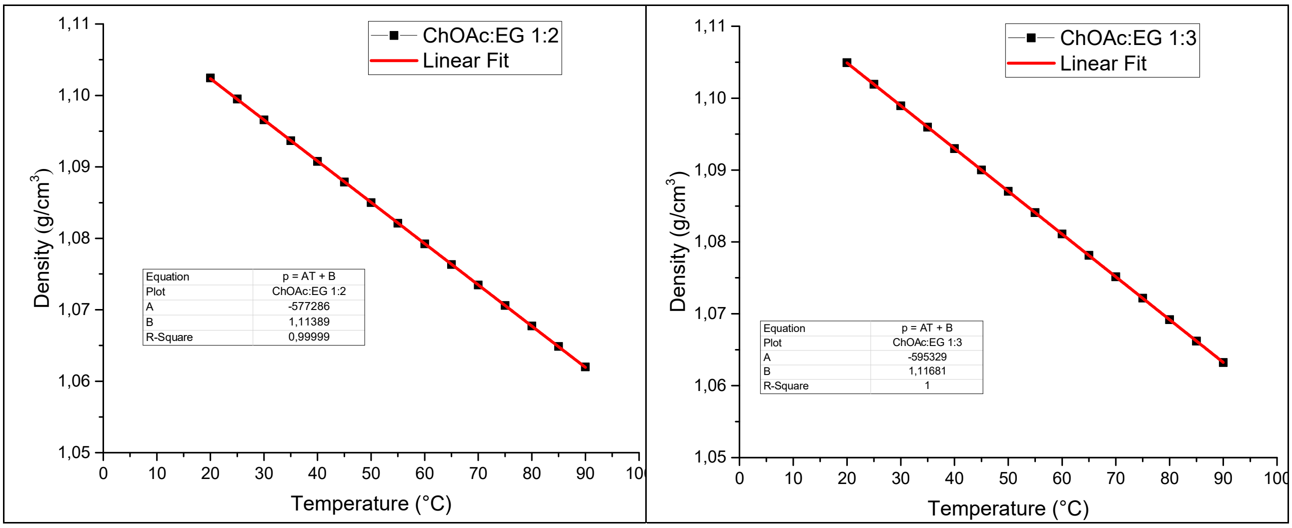

Figure S9. Fitting of density data of ChOAc:EG-based DESs.

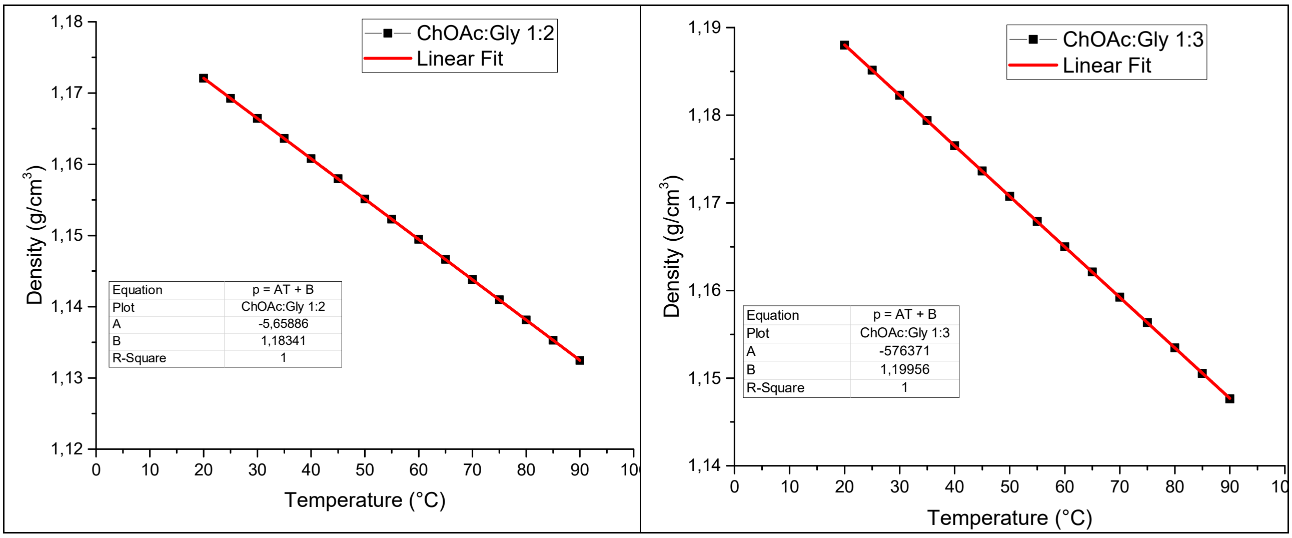

Figure S10. Fitting of density data of ChOAc:Gly-based DESs.

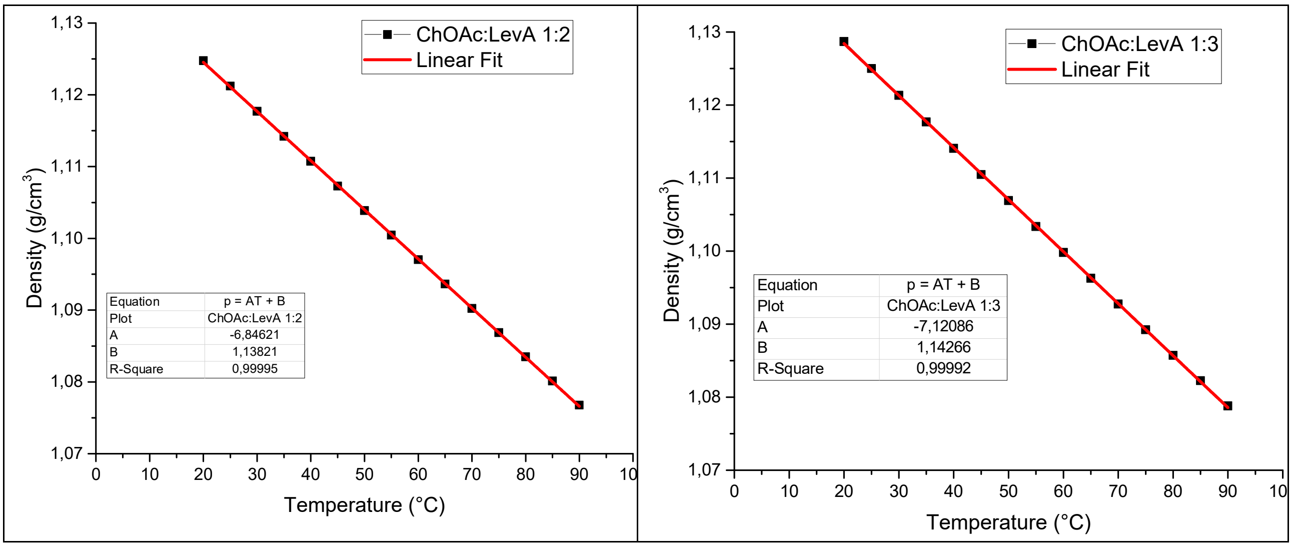

Figure S11. Fitting of density data of ChOAc:LevA-based DESs.

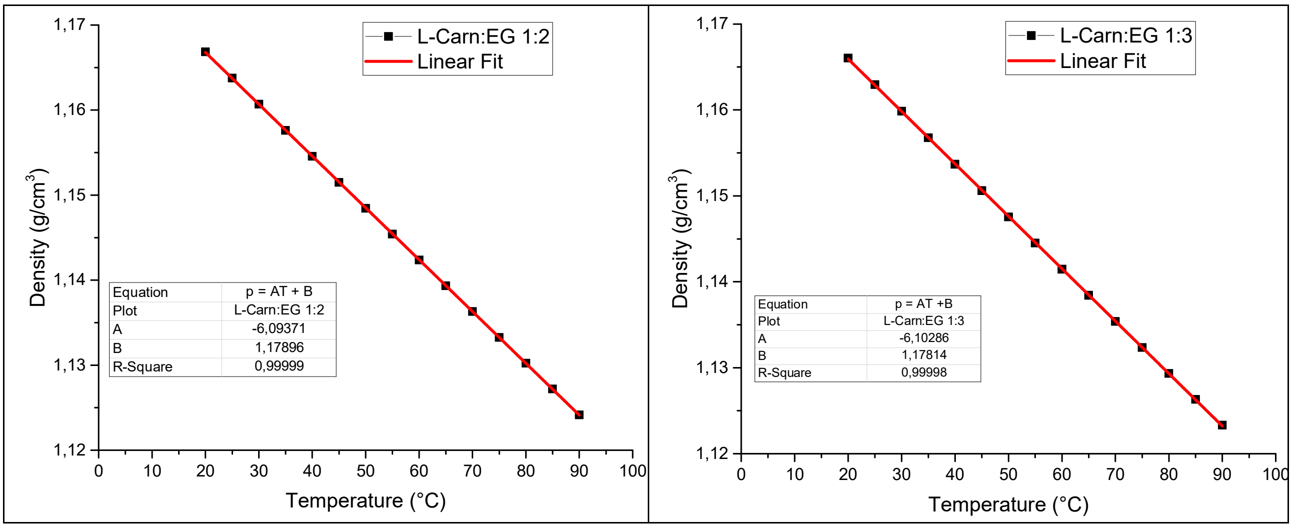

Figure S12. Fitting of density data of L-carn:EG-based DESs.

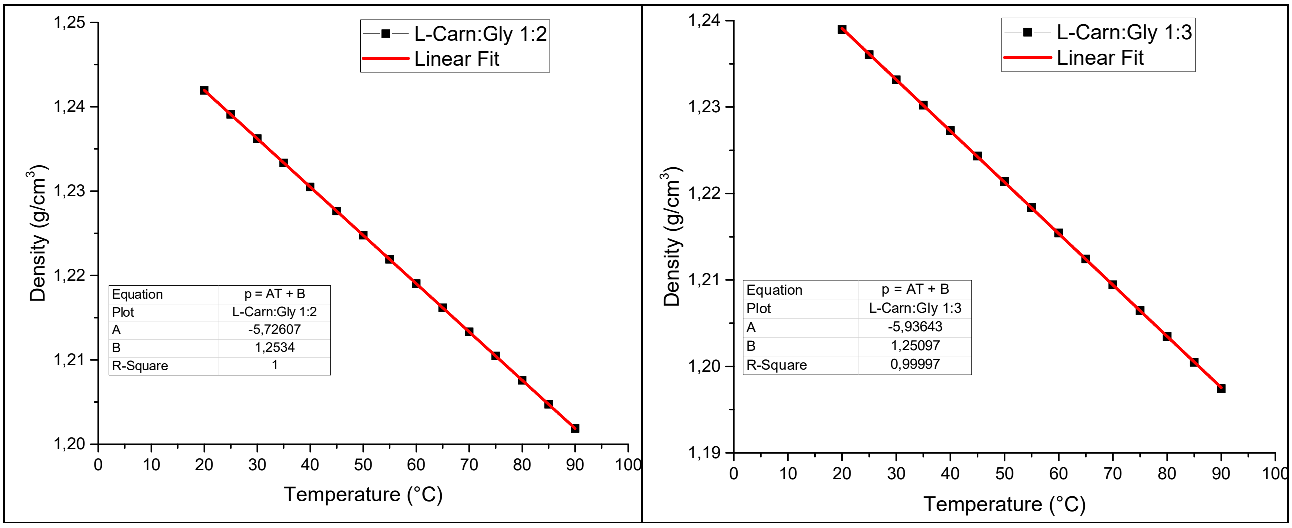

Figure S13. Fitting of density data of L-carn:Gly-based DESs

.

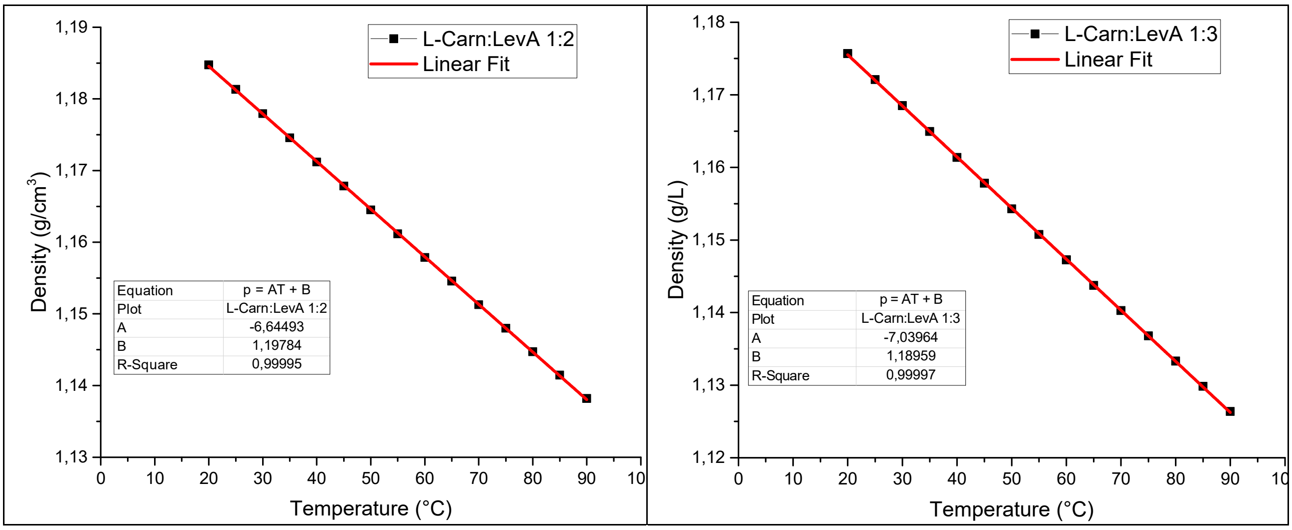

Figure S14. Fitting of density data of L-carn:LevA-based DESs.

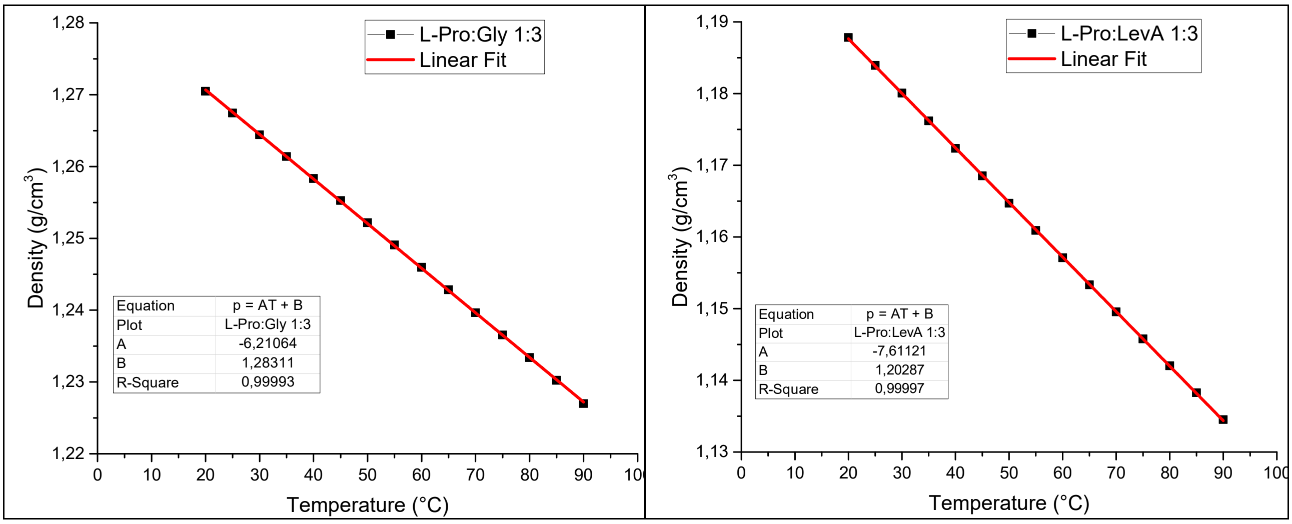

Figure S15. Fitting of density data of L-Pro-based DESs.

Table S4. Molar Volume data of ethylene glycol-based DESs.

	Molar Volume V_m (cm^3/mol)				
**T (°C)**	**EG**	**ChOAc:EG 1:2**	**ChOAc:EG 1:3**	**L-Carn:EG 1:2**	**L-Carn:EG 1:3**
**20**	55.84	260.65	316.32	244.54	297.75
**25**	56.02	261.35	317.19	245.19	298.52
**30**	56.20	262.04	318.05	245.83	299.31
**35**	56.38	262.74	318.92	246.49	300.11
**40**	56.56	263.44	319.79	247.14	300.90
**45**	56.74	264.14	320.66	247.80	301.70
**50**	56.93	264.84	321.53	248.46	302.50
**55**	57.12	265.55	322.41	249.12	303.31
**60**	57.31	266.26	323.30	249.78	304.11
**65**	57.50	266.97	324.19	250.44	304.92
**70**	57.70	267.68	325.09	251.11	305.73
**75**	57.90	268.40	325.99	251.78	306.55
**80**	58.10	269.12	326.90	252.46	307.38
**85**	58.31	269.85	327.81	253.14	308.21
**90**	58.52	270.57	328.74	253.83	309.04

Table S5. Molar Volume data of glycerol-based DESs.

	Molar Volume V_m (cm^3/mol)					
**T (°C)**	**Gly**	**ChOAc:Gly 1:2**	**ChOAc:Gly 1:3**	**L-Carn:Gly 1:2**	**L-Carn:Gly 1:3**	**L-Pro:Gly 1:3**
**20**	73.09	296.47	369.93	278.09	353.09	308.07
**25**	73.27	297.19	370.82	278.74	353.92	308.81
**30**	73.45	297.91	371.72	279.38	354.76	309.55
**35**	73.64	298.63	372.63	280.03	355.61	310.29
**40**	73.82	299.36	373.54	280.68	356.45	311.05
**45**	74.01	300.09	374.46	281.34	357.31	311.81
**50**	74.21	300.83	375.38	282.0	358.18	312.58
**55**	74.40	301.56	376.31	282.66	359.05	313.35
**60**	74.60	302.31	377.24	283.32	359.93	314.13
**65**	74.80	303.05	378.17	283.99	360.82	314.93
**70**	75.00	303.80	379.11	284.66	361.71	315.74
**75**	75.21	304.56	380.06	285.33	362.61	316.53
**80**	75.42	305.31	381.01	286.01	363.51	317.34
**85**	75.63	306.08	381.97	286.69	364.41	318.15
**90**	75.85	306.84	382.94	287.37	365.34	318.99

Table S6. Molar Volume data of levulinic acid-based DESs.

Molar Volume V_m (cm^3/mol)						
**T (°C)**	**LevA**	**ChOAc:LevA 1:2**	**ChOAc:LevA 1:3**	**L-Carn:LevA 1:2**	**L-Carn:LevA 1:3**	**L-Pro:LevA 1:3**
**20**	101.87	351.59	453.15	332.09	433.42	390.20
**25**	102.26	352.70	454.64	333.05	434.74	391.48
**30**	102.65	353.81	456.13	334.01	436.08	392.77
**35**	103.04	354.91	457.62	334.97	437.41	394.06
**40**	103.43	356.02	459.10	335.93	438.75	395.35
**45**	103.83	357.13	460.58	336.89	440.10	396.65
**50**	104.23	358.24	462.07	337.86	441.44	397.95
**55**	104.63	359.35	463.56	338.83	442.80	399.25
**60**	105.04	360.47	465.05	339.80	444.15	400.56
**65**	105.45	361.59	466.55	340.77	445.52	401.87
**70**	105.86	362.72	468.06	341.74	446.88	403.19
**75**	106.27	363.85	469.57	342.72	448.24	404.52
**80**	106.68	364.98	471.08	343.70	449.62	405.85
**85**	107.11	366.12	472.59	344.68	451.0	407.19
**90**	107.53	367.25	474.11	345.67	452.38	408.54

Table S7. Viscosity data of ethylene glycol-based DESs.

	Viscosity η (mPa·s)				
**T (°C)**	**EG**	**ChOAc:EG 1:2**	**ChOAc:EG 1:3**	**L-Carn:EG 1:2**	**L-Carn:EG 1:3**
**20**	17.91	95.016	60.364	708.297	589.007
**25**	15.18	76.364	49.209	513.47	431.79
**30**	12.69	61.713	40.287	375.47	319.23
**35**	10.77	50.708	33.491	281.865	241.03
**40**	9.46	42.241	28.169	216.16	186.02
**45**	8.33	35.607	24.166	168.575	146
**50**	7.08	30.379	20.703	133.735	116.64
**55**	6.35	26.145	18.185	107.72	94.493
**60**	5.61	22.833	15.887	88.03	77.817
**65**	4.98	20.003	14.094	73	64.823
**70**	4.49	17.824	12.517	61.52	54.8125
**75**	4.09	15.809	11.337	53.965	46.919
**80**	3.62	14.369	10.289	45.957	40.794
**85**	3.41	12.917	9.331	42.188	35.9035
**90**	3.21	11.949	8.606	37.974	31.888

Table S8. Viscosity data of glycerol-based DESs.

	Viscosity η (mPa·s)					
**T (°C)**	**Gly**	**ChOAc:Gly 1:2**	**ChOAc:Gly 1:3**	**L-Carn:Gly 1:2**	**L-Carn:Gly 1:3**	**L-Pro:Gly 1:3**
**20**	1365.25	940.9	846.97	22622.25	8161.45	5989.9
**25**	913.64	672.08	593.6	13699	5190.1	3795
**30**	611.07	481.17	419.71	8380.7	3324.45	2423.7
**35**	420.25	353.08	304.27	5311.9	2194.9	1595.7
**40**	296.45	264.74	225.74	3473.6	1489.1	1081.1
**45**	214.18	202.55	171.23	2340.75	1038.455	752.67
**50**	158.35	157.91	132.5	1617.65	740.21	537.29
**55**	119.37	125.17	104.37	1159.2	539.14	392.59
**60**	91.542	100.61	83.572	835.915	400.595	293.53
**65**	62.472	82.392	68.068	617.795	303.83	224.11
**70**	56.935	68.11	56.21	464.115	234.39	174.63
**75**	45.674	57.038	47.053	336.25	191.425	138.55
**80**	37.861	48.4	39.805	278.48	151.585	111.67
**85**	31.433	41.441	34.166	220.6	121.925	91.34
**90**	26.203	35.923	29.584	177.57	99.814	75.87

Table S9. Viscosity data of levulinic acid-based DESs.

Viscosity η (mPa·s)						
**T (°C)**	**LevA**	**ChOAc:LevA 1:2**	**ChOAc:LevA 1:3**	**L-Carn:LevA 1:2**	**L-Carn:LevA 1:3**	**L-Pro:LevA 1:3**
**20**	40.33	175.945	143.98	7228.4	1543.15	540.08
**25**	31.75	102.75	111.11	4381.65	1013.65	373.67
**30**	25.07	80.657	85.3155	2696.15	673.795	264.285
**35**	20.11	64.972	67.1205	1731.05	464.81	194.86
**40**	16.75	53.365	53.874	1154.9	331.27	148.35
**45**	13.87	44.457	44.181	797.435	242.86	116.44
**50**	11.8	38.101	37.031	567.205	182.905	93.883
**55**	10.2	32.719	31.531	414.75	141.075	77.365
**60**	8.86	28.618	27.118	311.34	111.16	63.955
**65**	7.8	25.553	23.464	238.79	89.338	53.628
**70**	6.97	25.553	20.596	187.2	73.263	44.751
**75**	6.26	22.819	18.428	149.6	61.029	38.027
**80**	5.94	20.5575	16.7195	121.55	51.4545	32.688
**85**	5.21	18.6875	15.343	100.45	43.887	28.6375
**90**	4.6	16.937	14.057	84.317	37.889	25.451

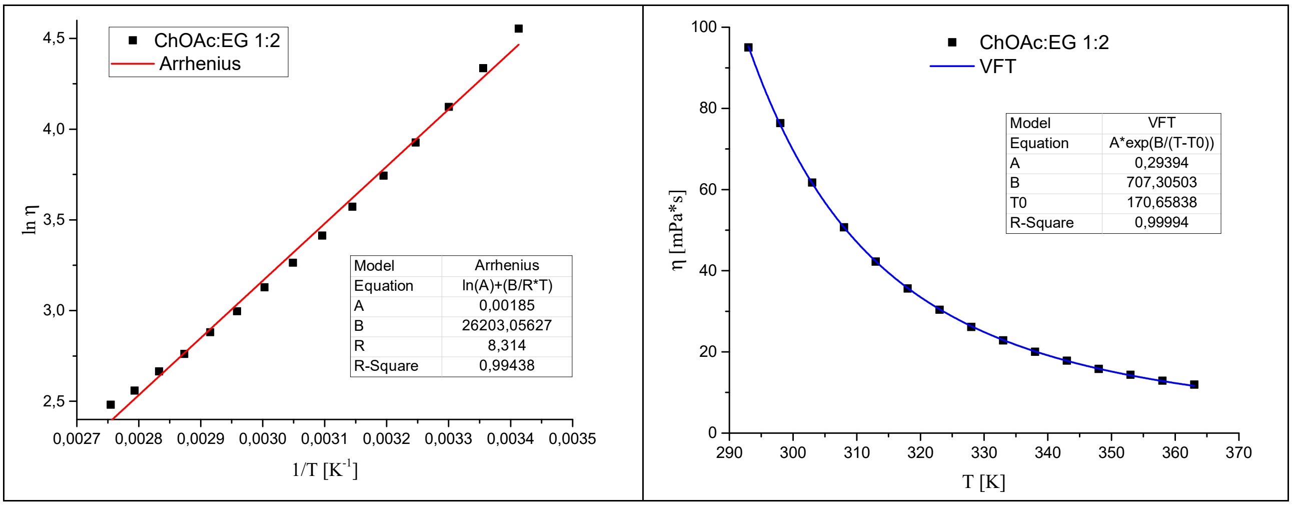

Figure S16. Fitting of viscosity data of ChOAc:EG 1:2 according Arrhenius (left) and VFT model (right).

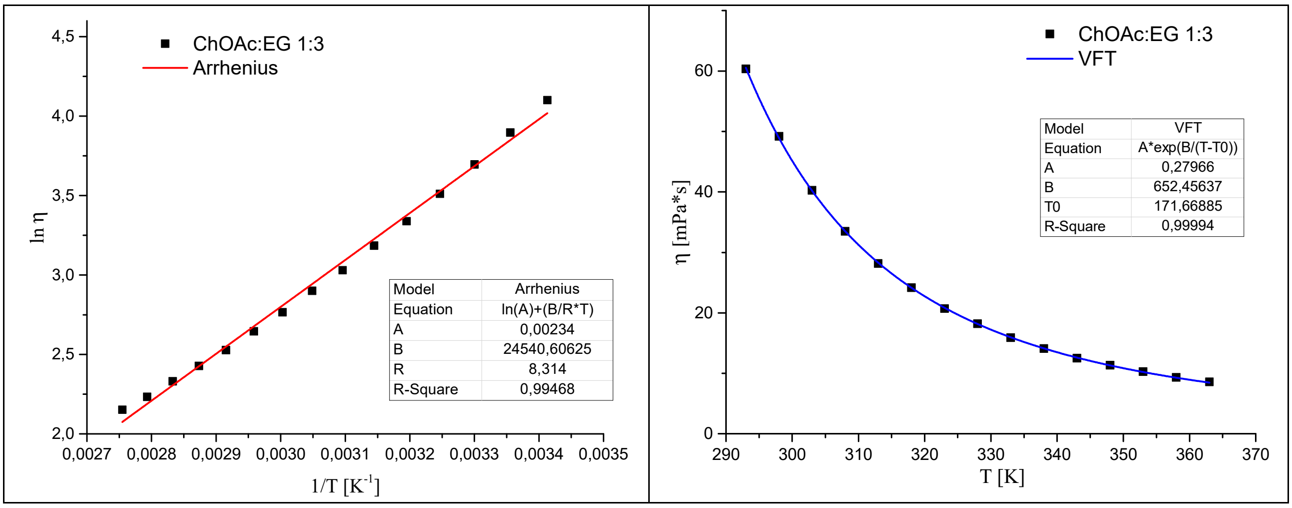

Figure S17. Fitting of viscosity data of ChOAc:EG 1:3 according Arrhenius (left) and VFT model (right).

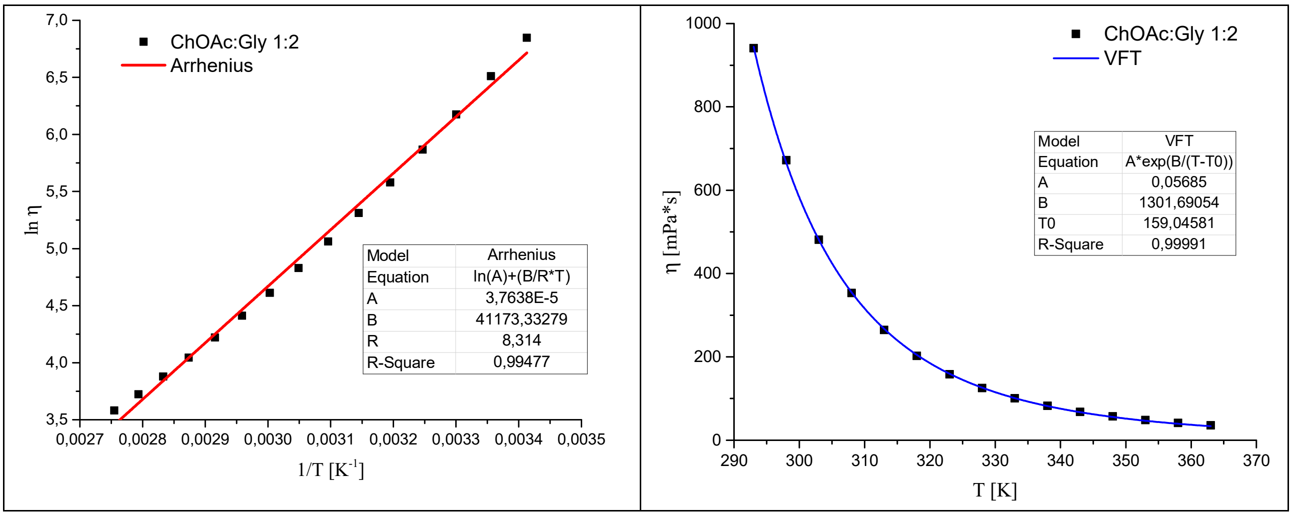

Figure S18. Fitting of viscosity data of ChOAc:Gly 1:2 according Arrhenius (left) and VFT model (right).

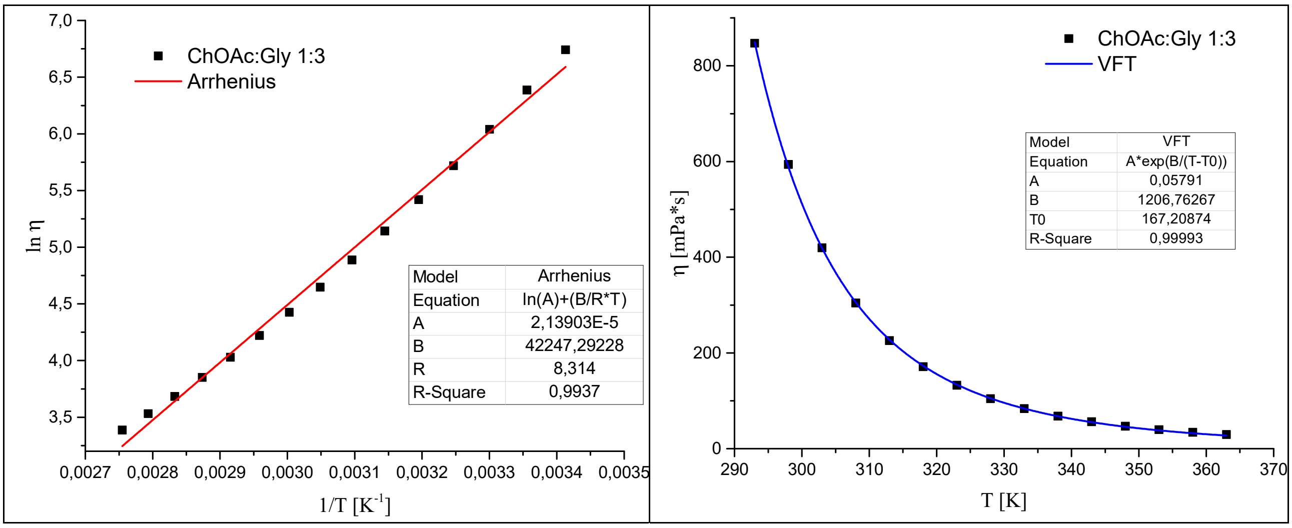

Figure S19. Fitting of viscosity data of ChOAc:Gly 1:3 according Arrhenius (left) and VFT model (right).

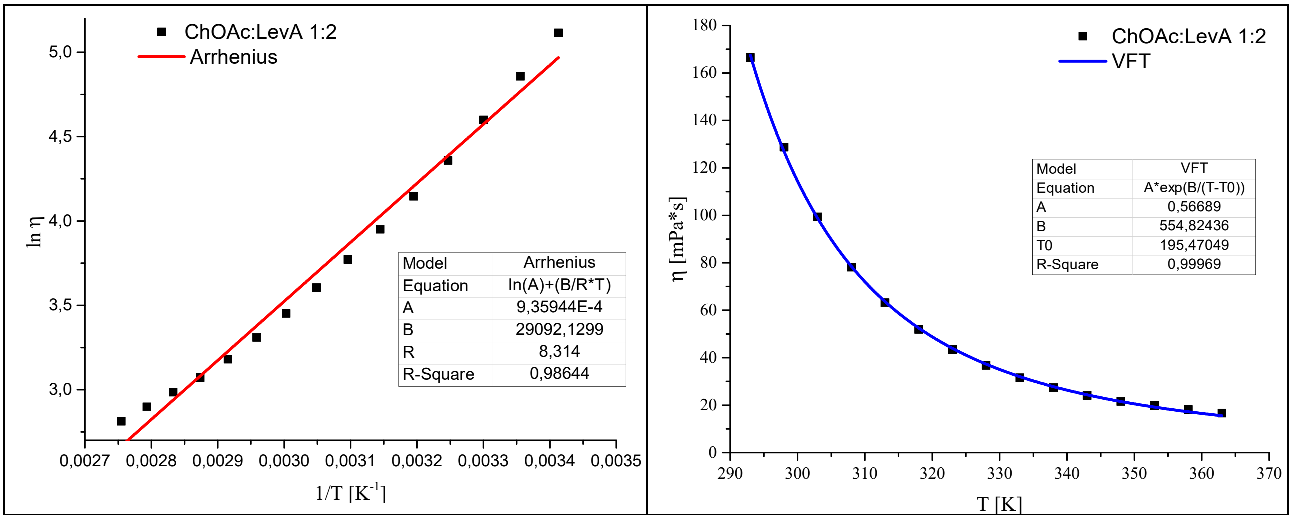

Figure S20. Fitting of viscosity data of ChOAc:LevA 1:2 according Arrhenius (left) and VFT model (right).

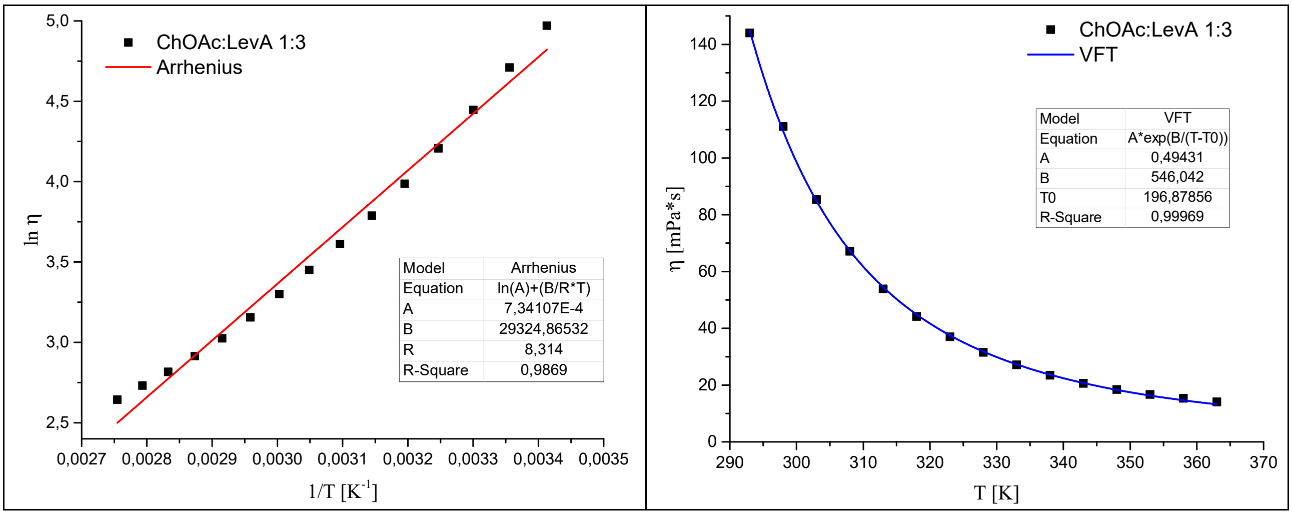

Figure S21. Fitting of viscosity data of ChOAc:LevA 1:3 according Arrhenius (left) and VFT model (right).

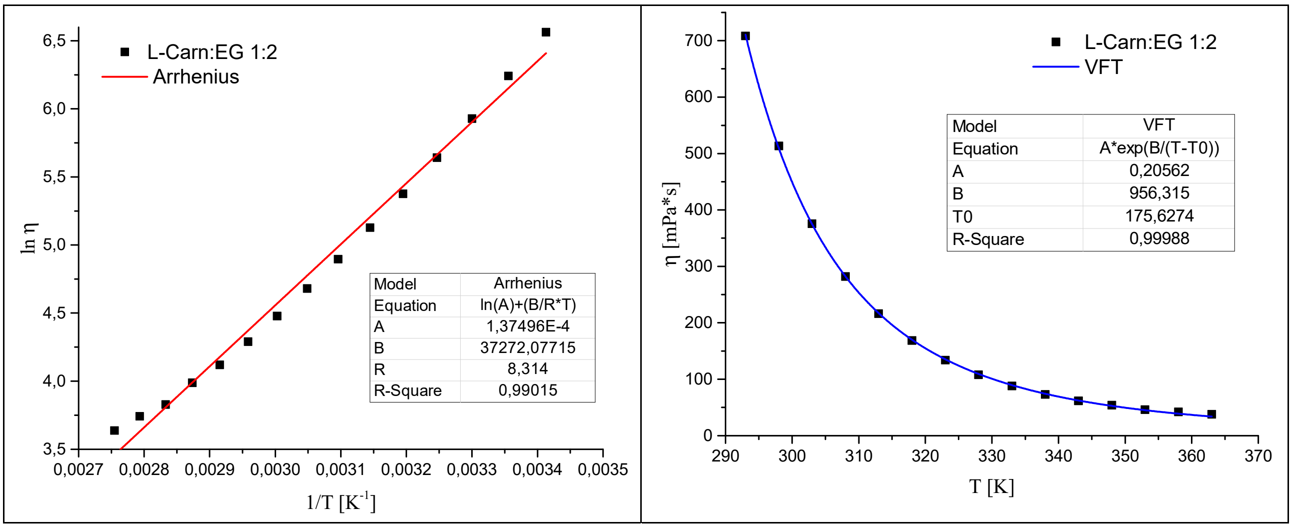

Figure S22. Fitting of viscosity data of L-Carn:EG 1:2 according Arrhenius (left) and VFT model (right).

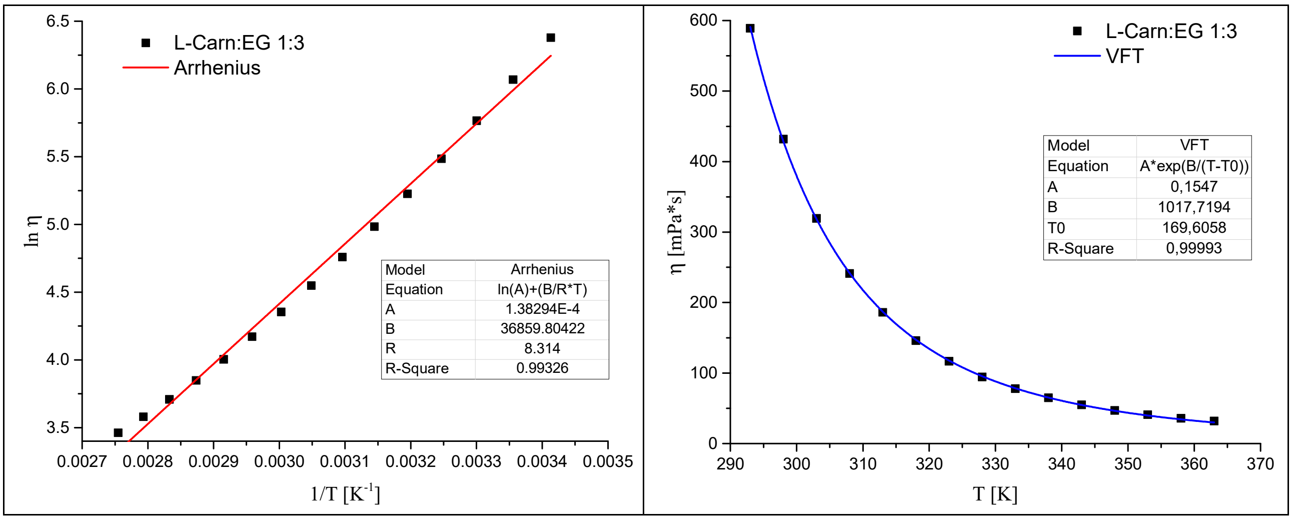

Figure S23. Fitting of viscosity data of L-Carn:EG 1:3 according Arrhenius (left) and VFT model (right).

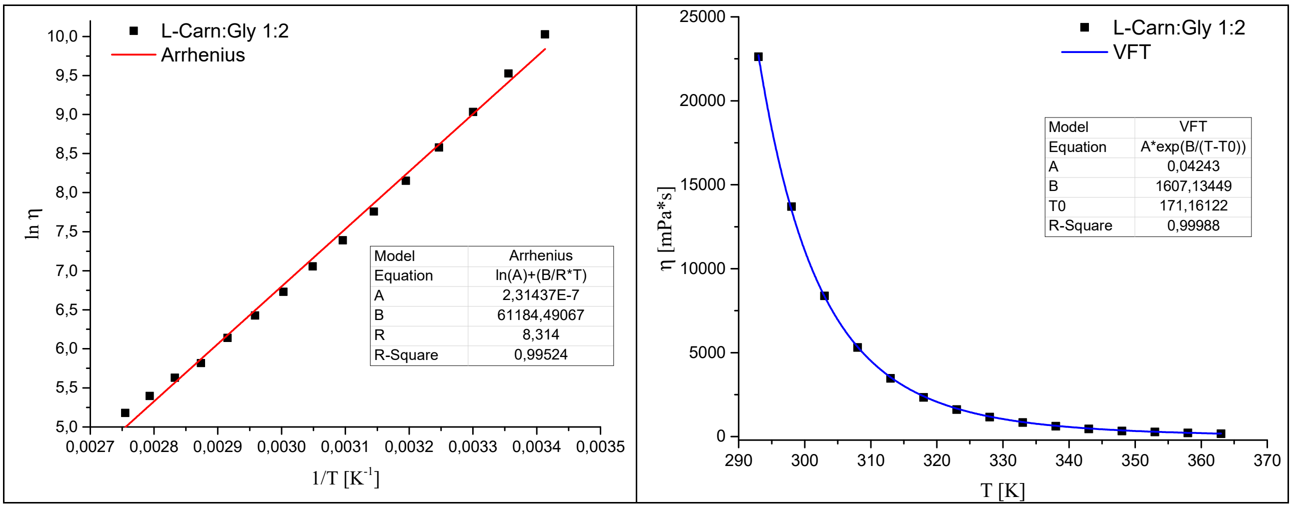

Figure S24. Fitting of viscosity data of L-Carn:Gly 1:2 according Arrhenius (left) and VFT model (right).

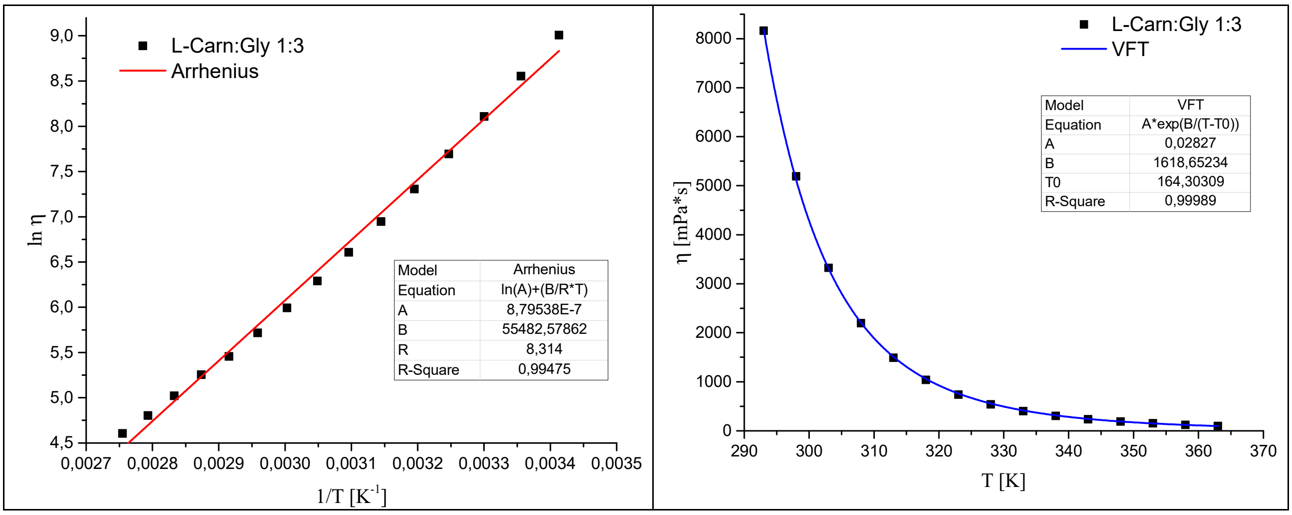

Figure S25. Fitting of viscosity data of L-Carn:Gly 1:3 according Arrhenius (left) and VFT model (right).

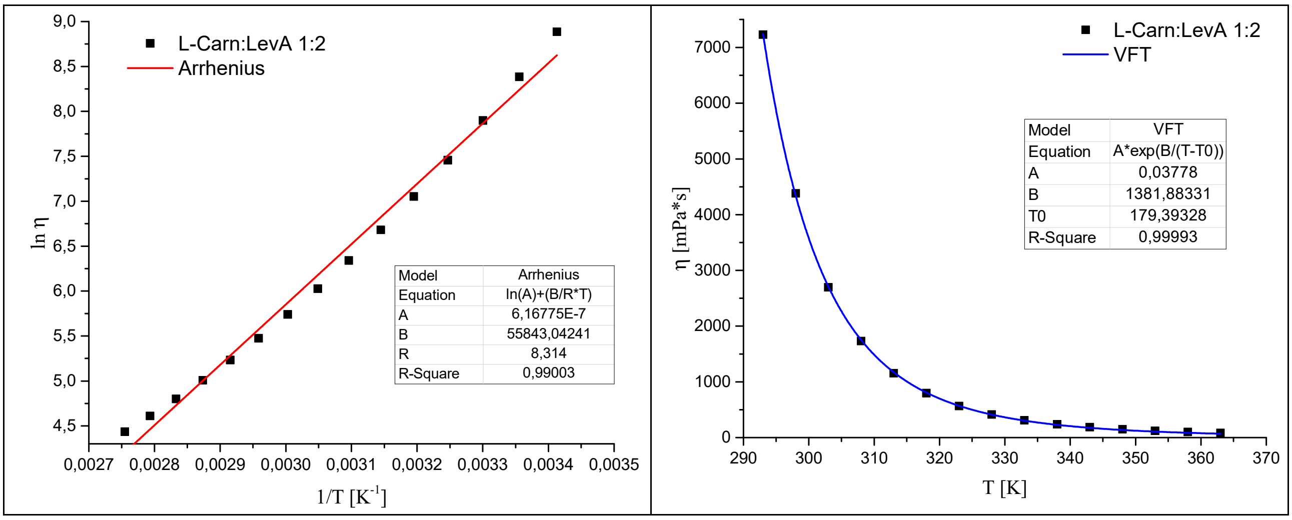

Figure S26. Fitting of viscosity data of L-Carn:LevA 1:2 according Arrhenius (left) and VFT model (right).

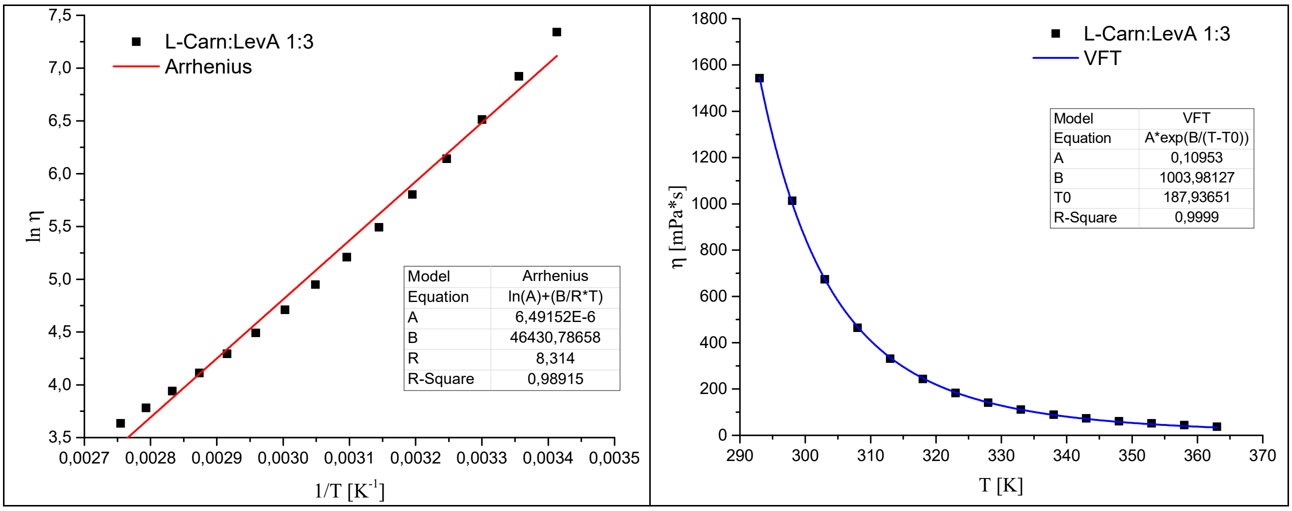

Figure S27. Fitting of viscosity data of L-Carn:LevA 1:3 according Arrhenius (left) and VFT model (right).

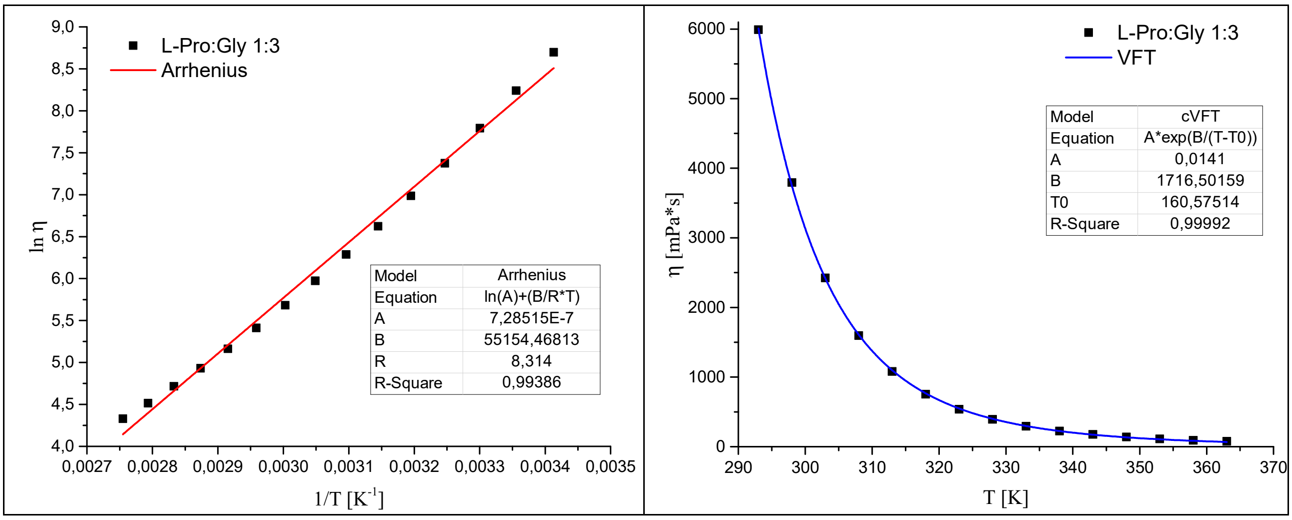

Figure S28. Fitting of viscosity data of L-Pro:Gly 1:3 according Arrhenius (left) and VFT model (right).

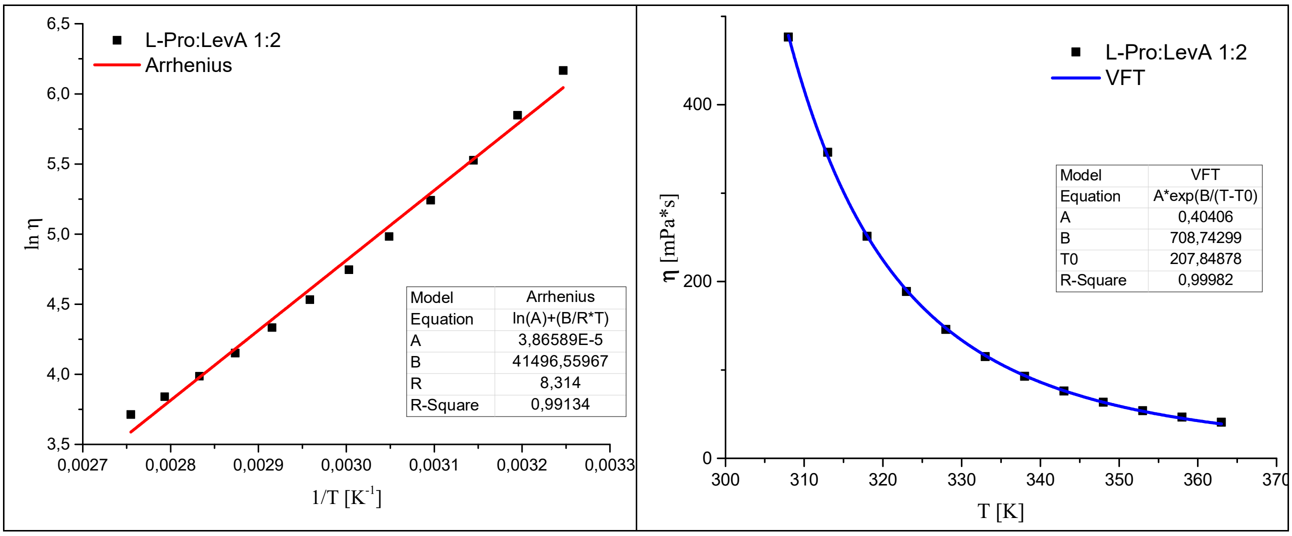

Figure S29. Fitting of viscosity data of L-Pro:LevA 1:2 according Arrhenius (left) and VFT model (right).

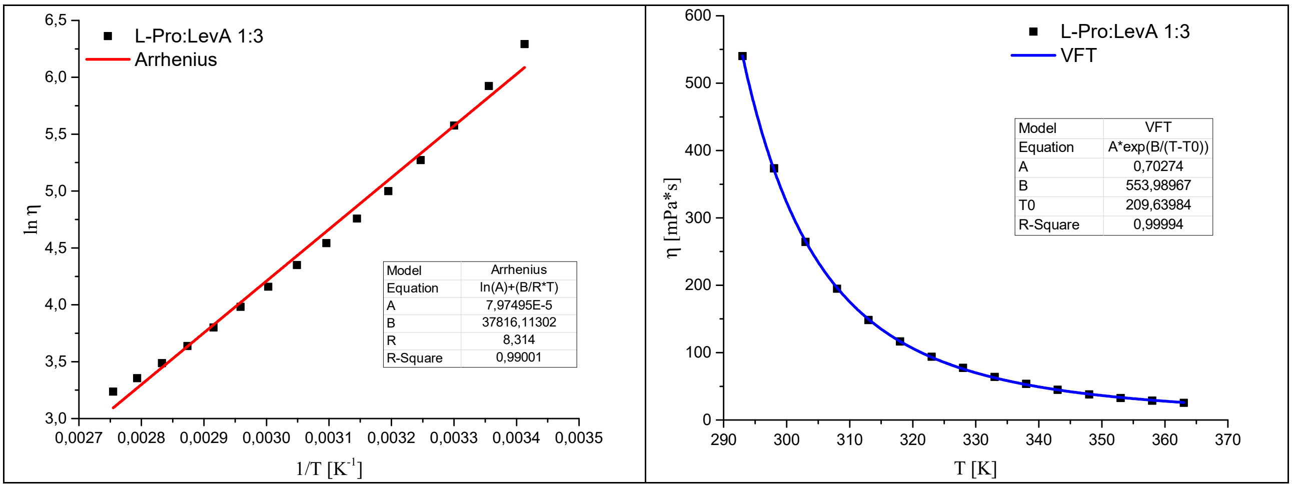

Figure S30. Fitting of viscosity data of L-Pro:LevA 1:3 according Arrhenius (left) and VFT model (right).

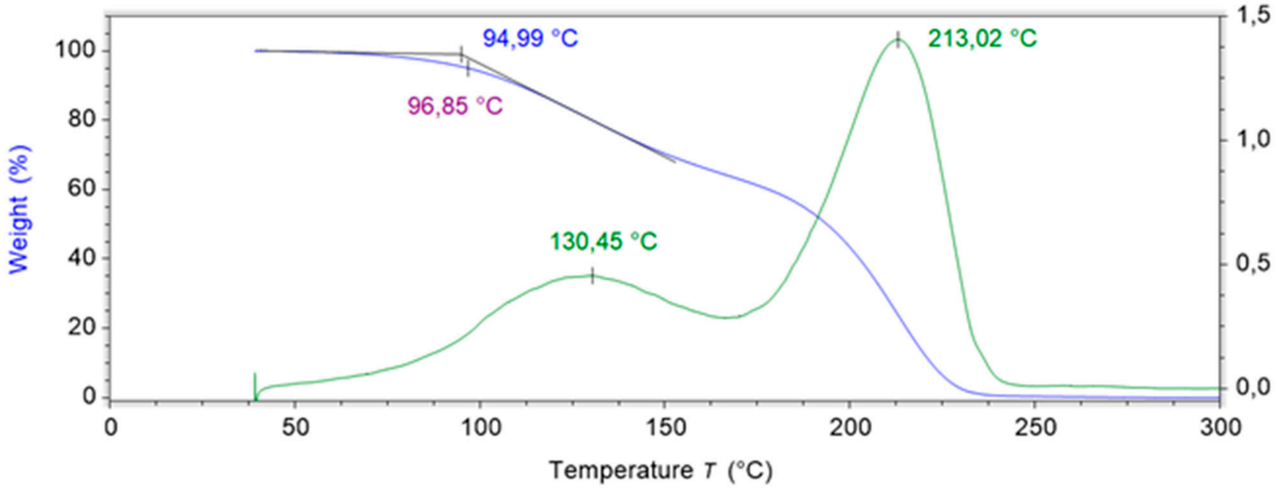

Figure S31. TGA and *d*weight% vs Temperature plots of ChOAc:EG 1:2 DES.

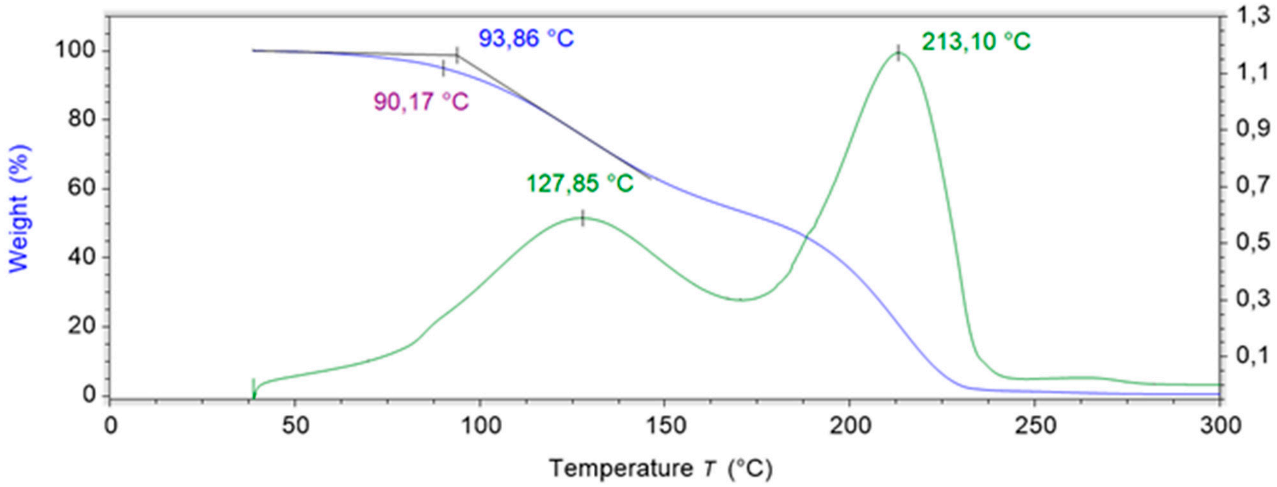

Figure S32. TGA and *d*weight% vs Temperature plots of ChOAc:EG 1:3 DES.

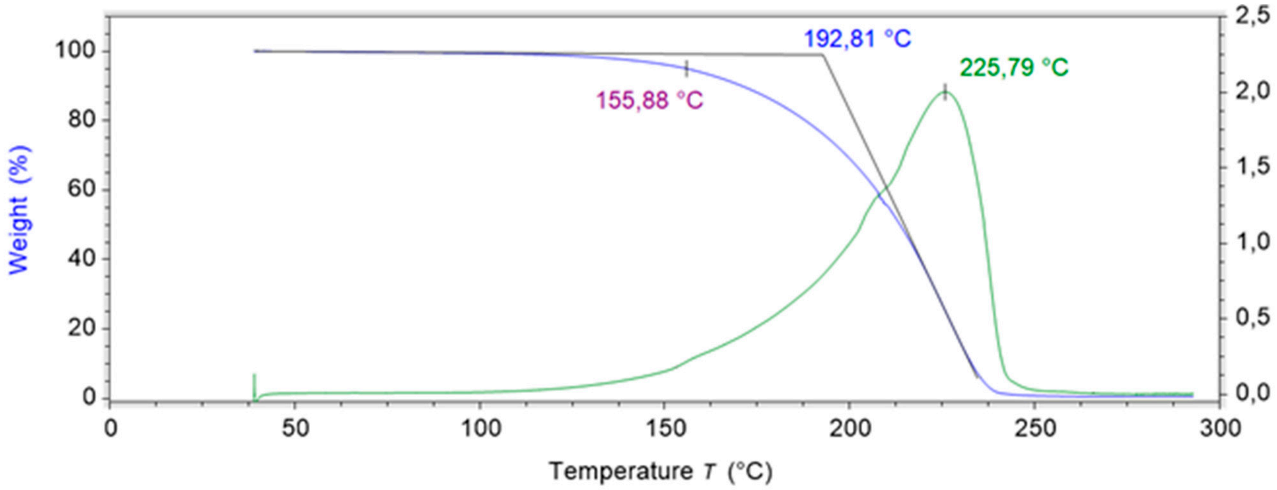

Figure S33. TGA and *d*weight% vs Temperature plots of ChOAc:Gly 1:2 DES.

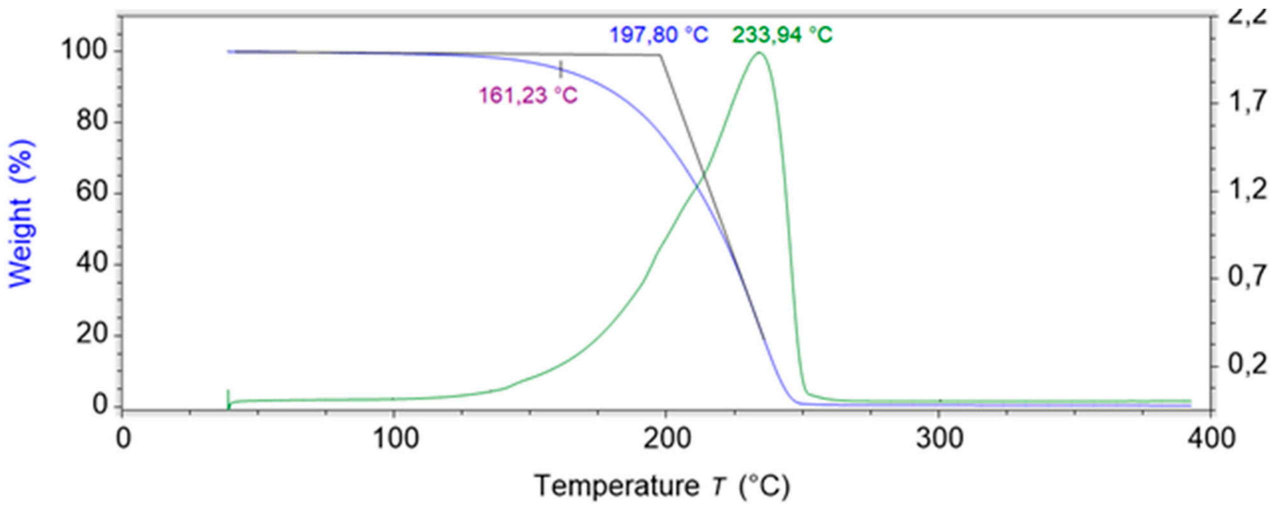

Figure S34. TGA and *d*weight% vs Temperature plots of ChOAc:Gly 1:3 DES.

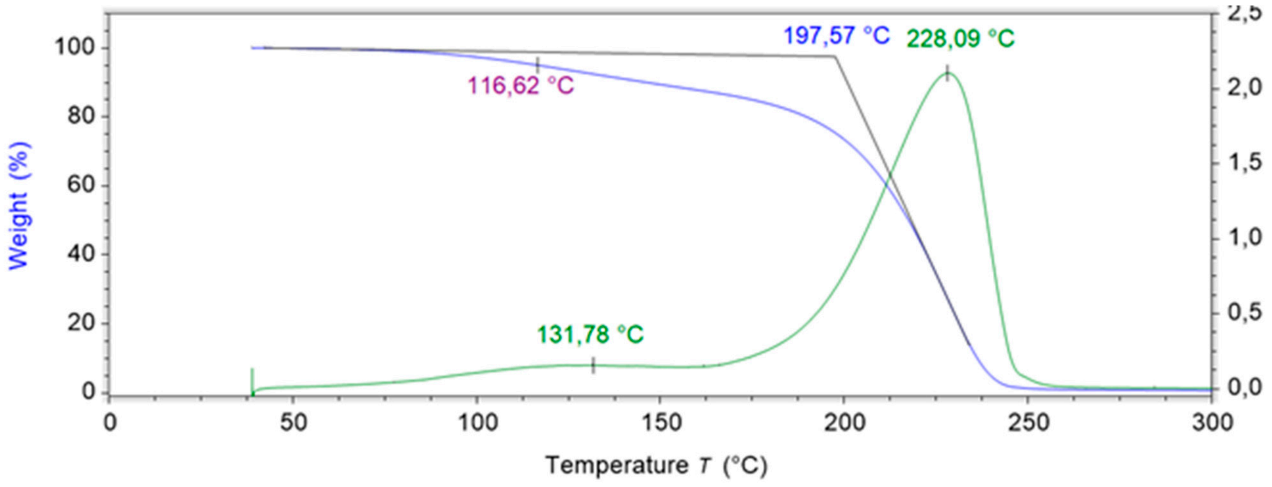

Figure S35. TGA and *d*weight% vs Temperature plots of ChOAc:LevA 1:2 DES.

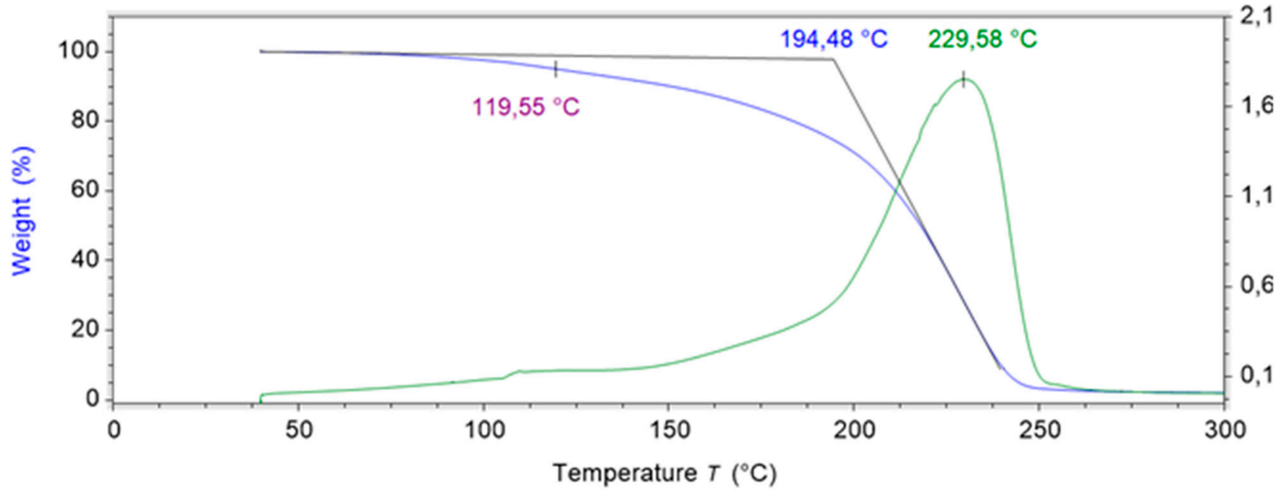

Figure S36. TGA and *d*weight% vs Temperature plots of ChOAc:LevA 1:3 DES.

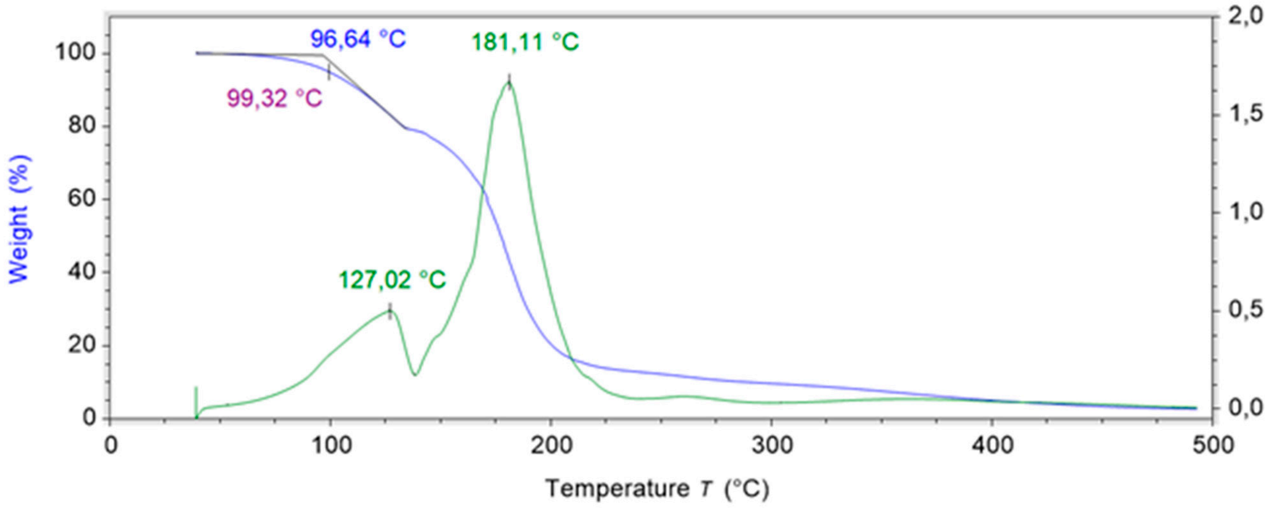

Figure S37. TGA and *d*weight% vs Temperature plots of L-Carn:EG 1:2 DES.

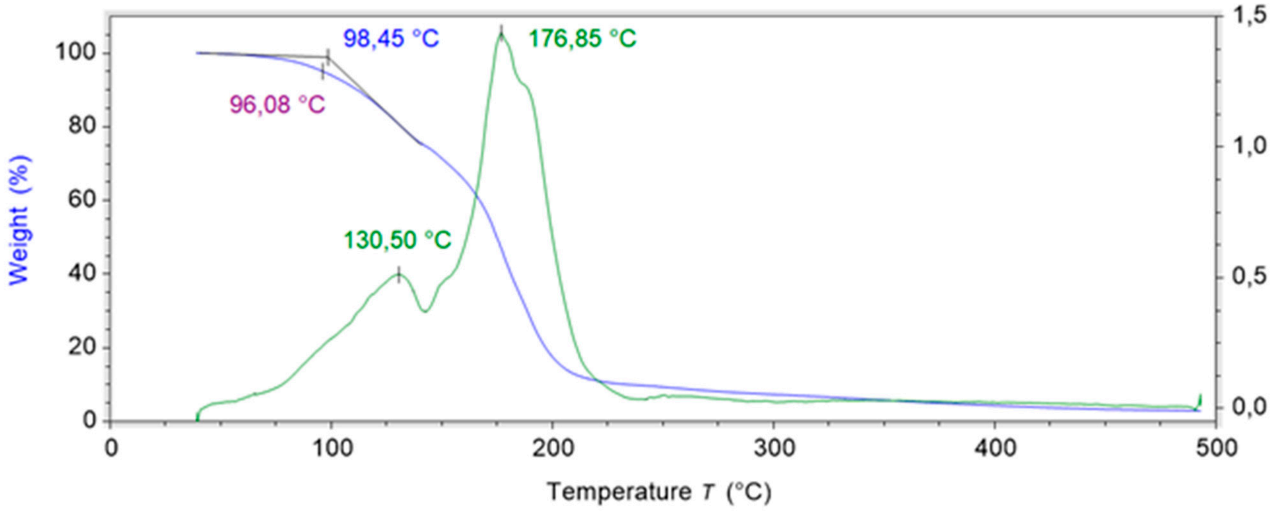

Figure S38. TGA and *d*weight% vs Temperature plots of L-Carn:EG 1:3 DES.

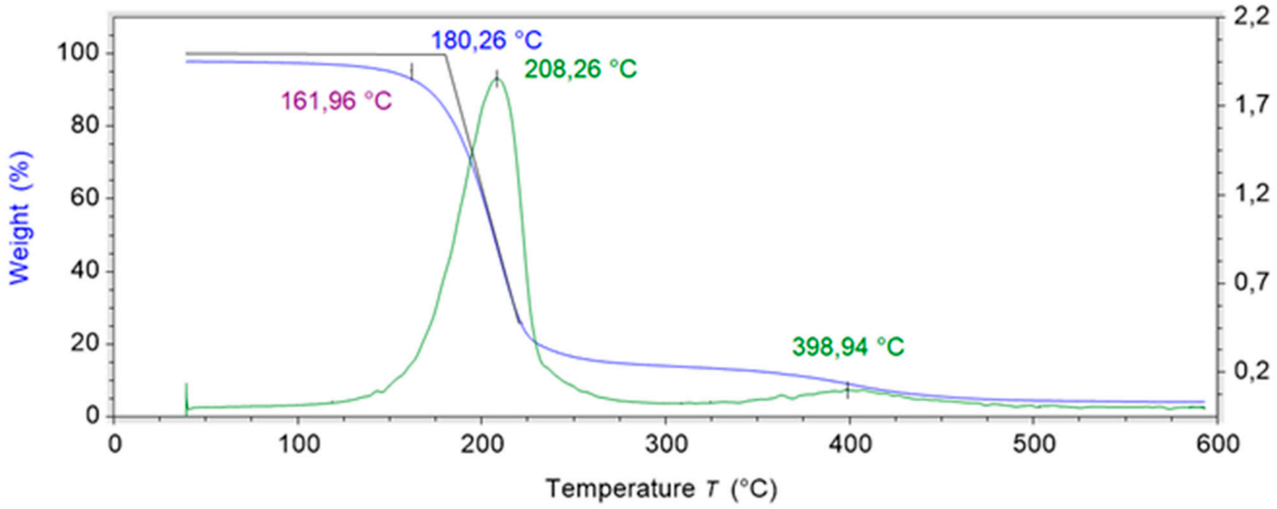

Figure S39. TGA and *d*weight% vs Temperature plots of L-Carn:Gly 1:2 DES.

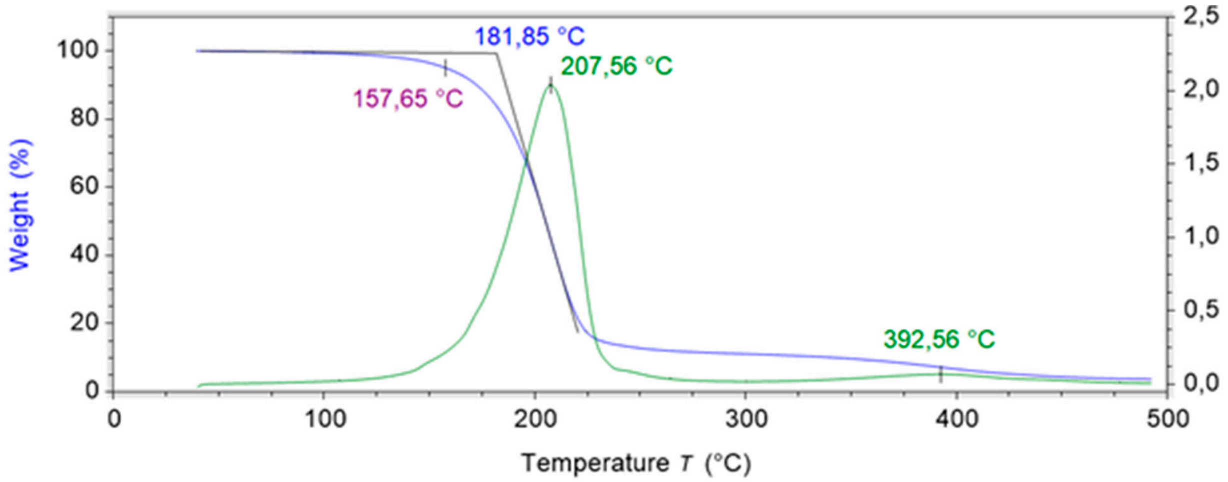

Figure S40. TGA and *d*weight% vs Temperature plots of L-Carn:Gly 1:3 DES.

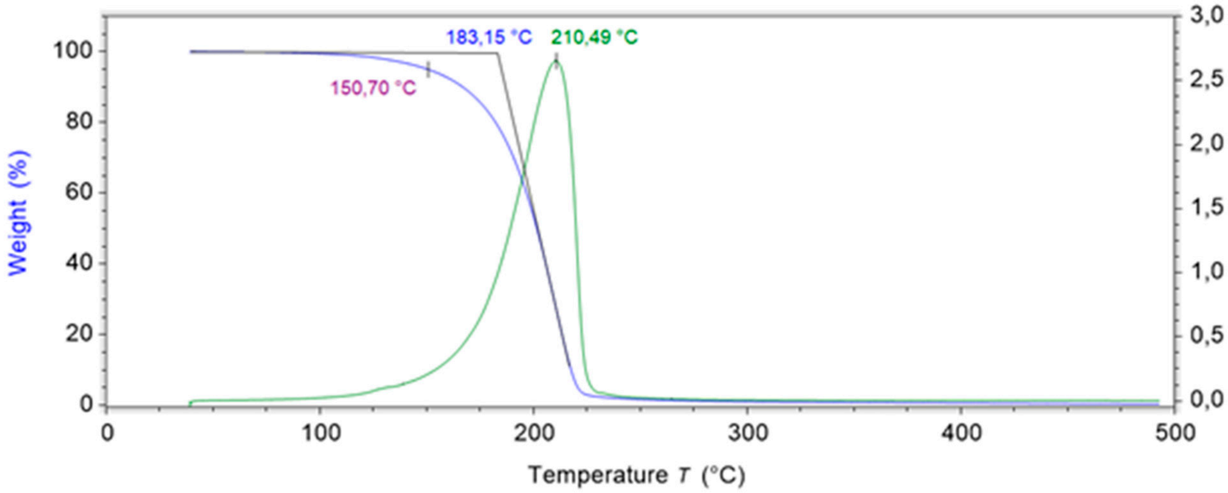

Figure S41. TGA and *d*weight% vs Temperature plots of L-Carn:LevA 1:2 DES.

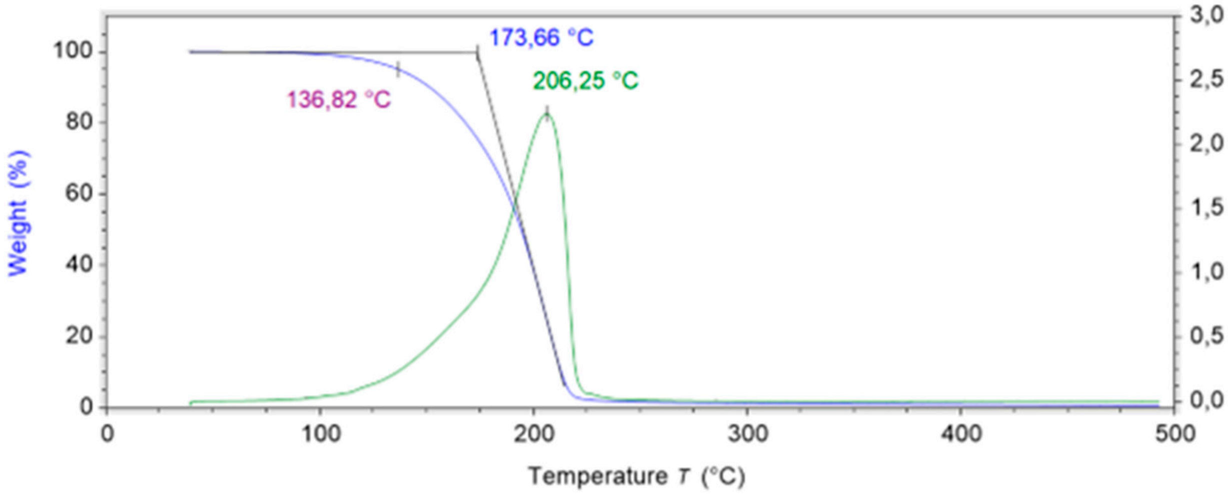

Figure S42. TGA and *d*weight% vs Temperature plots of L-Carn:LevA 1:3 DES.

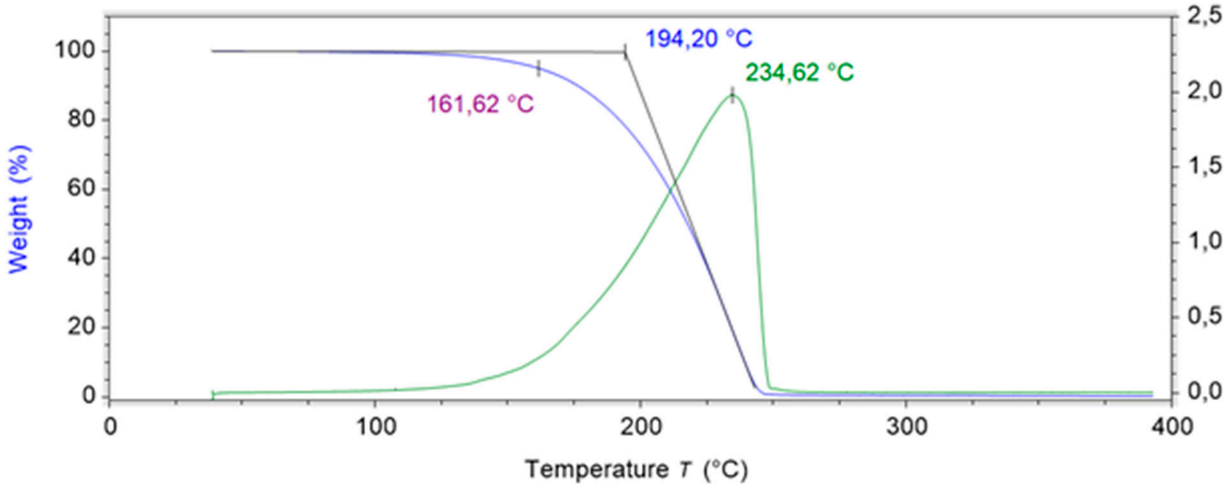

Figure S43. TGA and *d*weight% vs Temperature plots of L-Pro:Gly 1:3 DES.

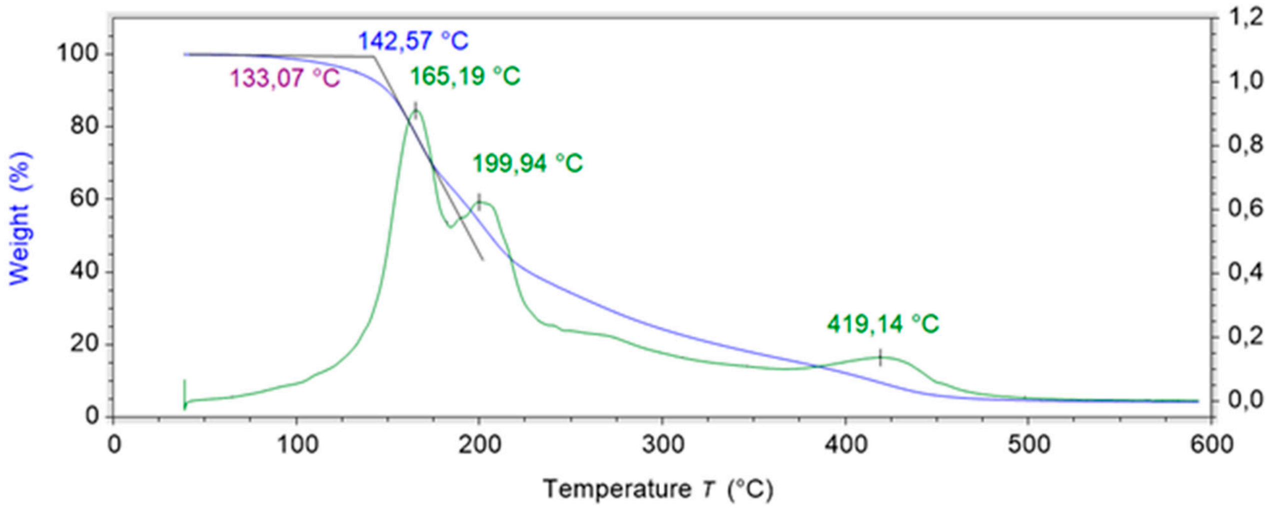

Figure S44. TGA and *d*weight% vs Temperature plots of L-Pro:LevA 1:2 DES.

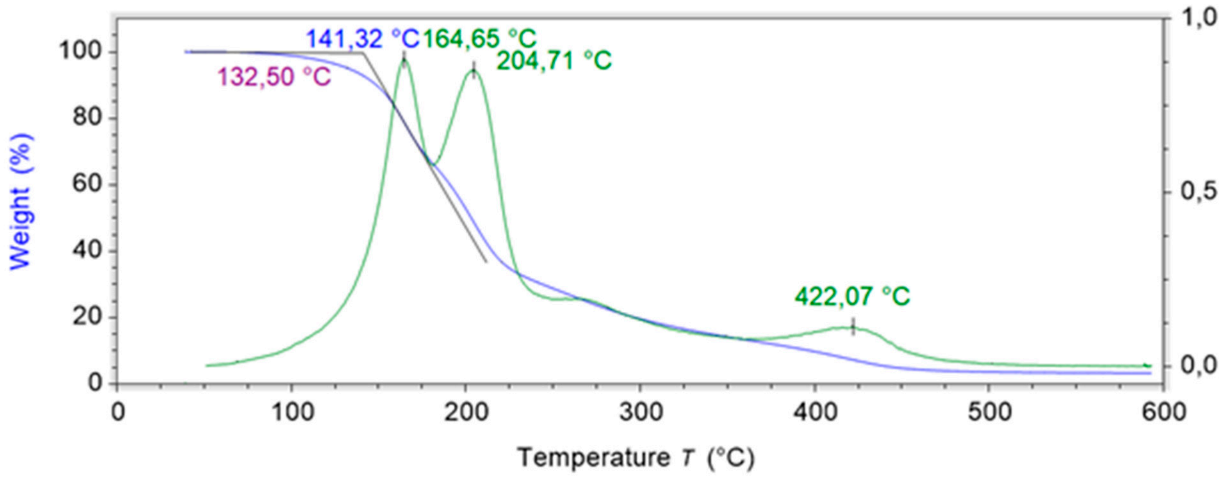

Figure S45. TGA and *d*weight% vs Temperature plots of L-Pro:LevA 1:3 DES.

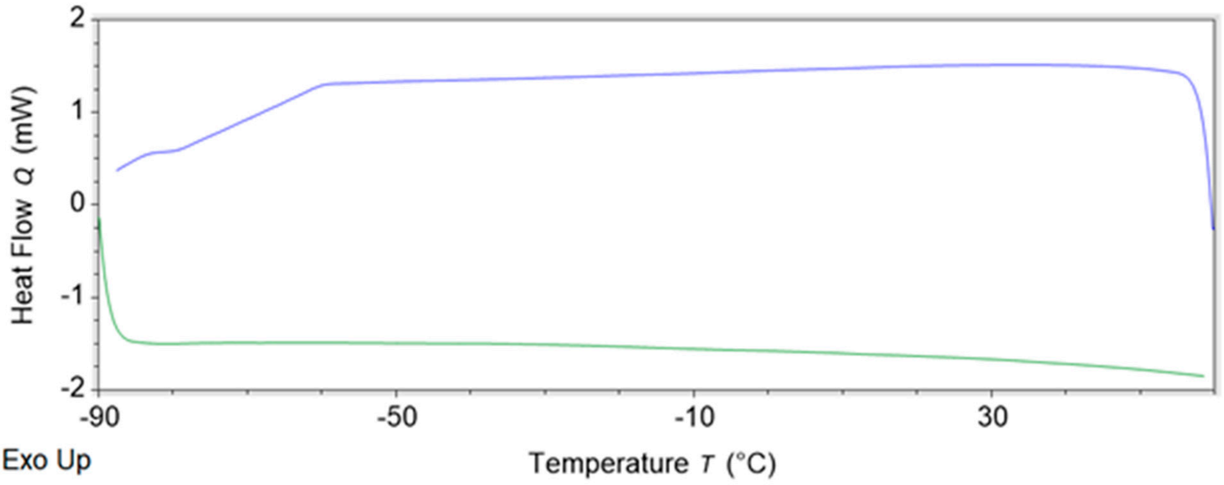

Figure S46. DSC of ChOAc:EG 1:2 DES. The heating run at 10 °C/min is reported in green while the cooling run in blue.

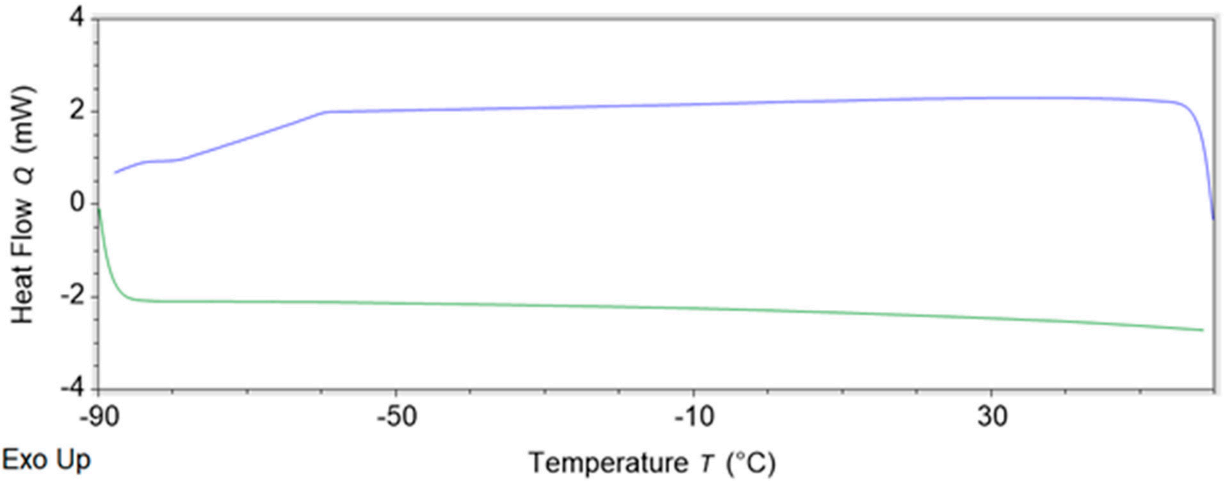

Figure S47. DSC of ChOAc:EG 1:3 DES. The heating run at 10 °C/min is reported in green while the cooling run in blue.

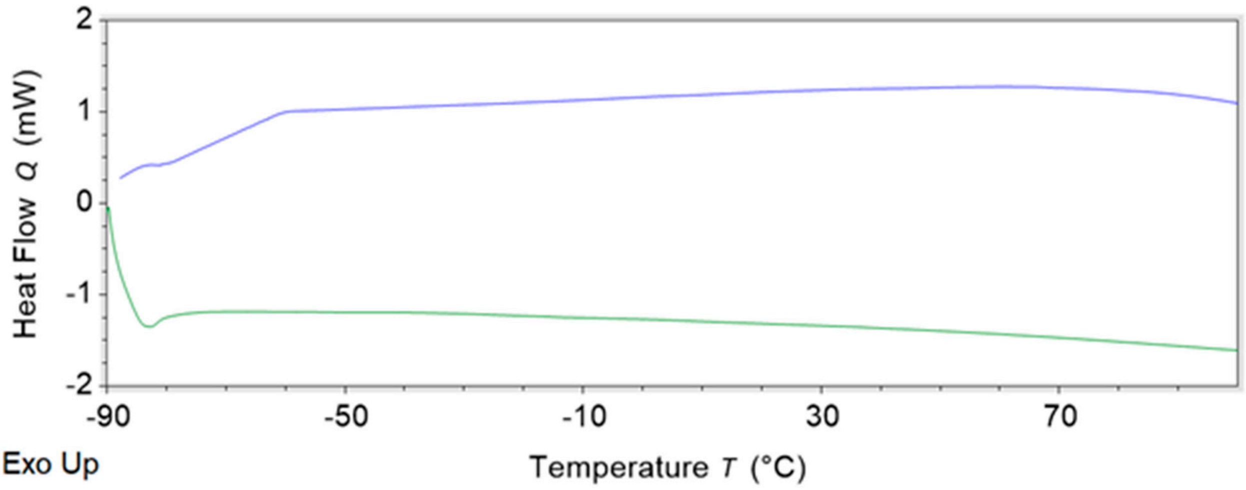

Figure S48. DSC of ChOAc:Gly 1:2 DES. The heating run at 10 °C/min is reported in green while the cooling run in blue.

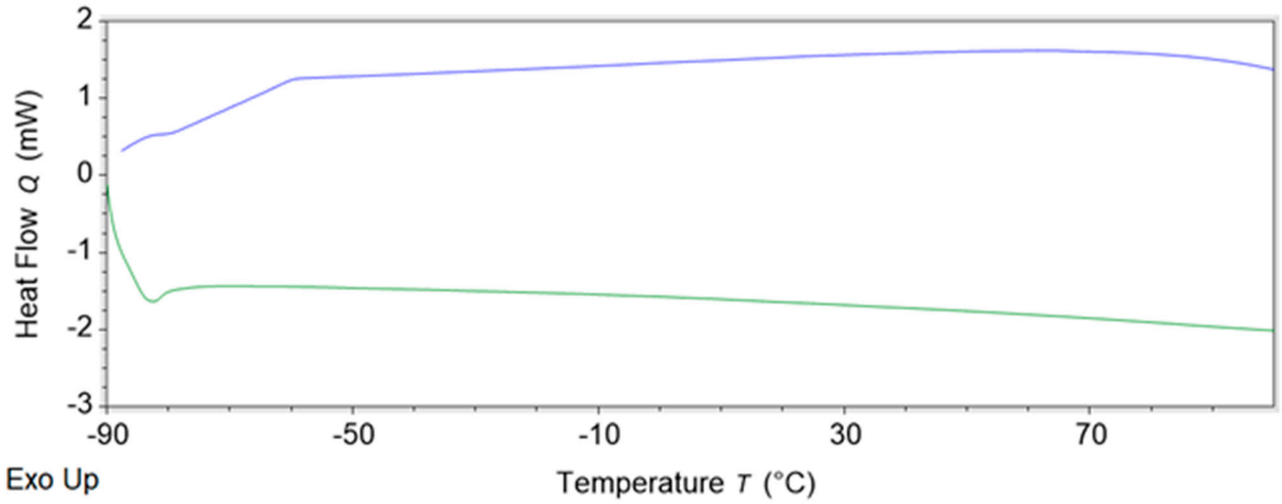

Figure S49. DSC of ChOAc:Gly 1:3 DES. The heating run at 10 °C/min is reported in green while the cooling run in blue.

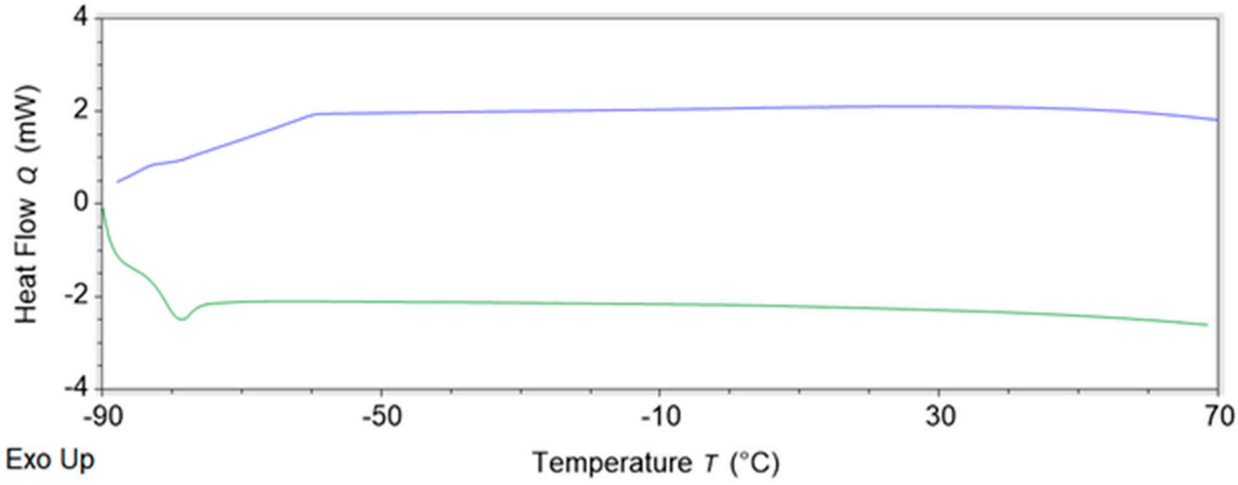

Figure S50. DSC of ChOAc:LevA 1:2 DES. The heating run at 10 °C/min is reported in green while the cooling run in blue.

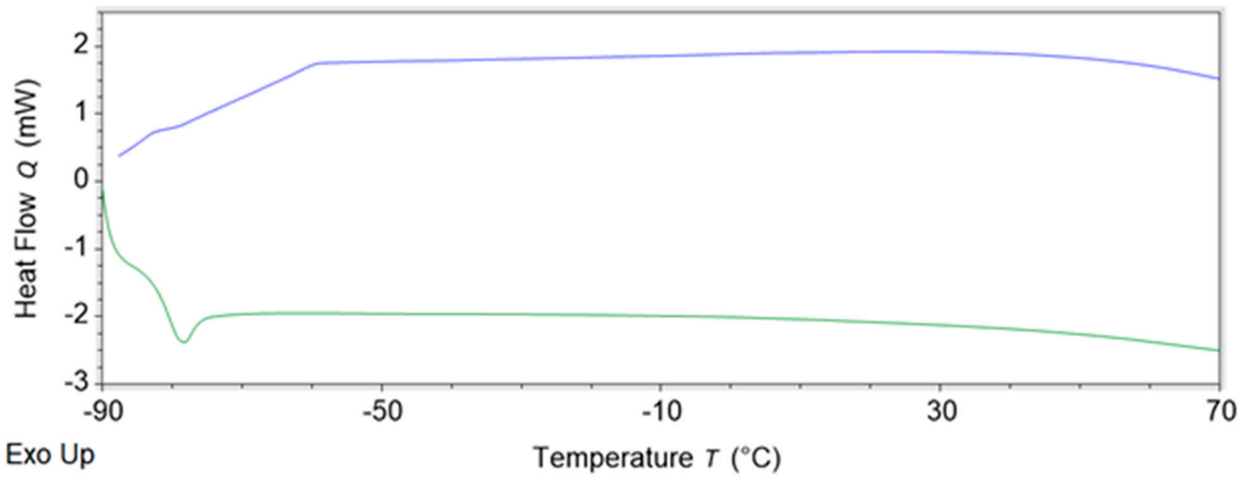

Figure S51. DSC of ChOAc:LevA 1:3 DES. The heating run at 10 °C/min is reported in green while the cooling run in blue.

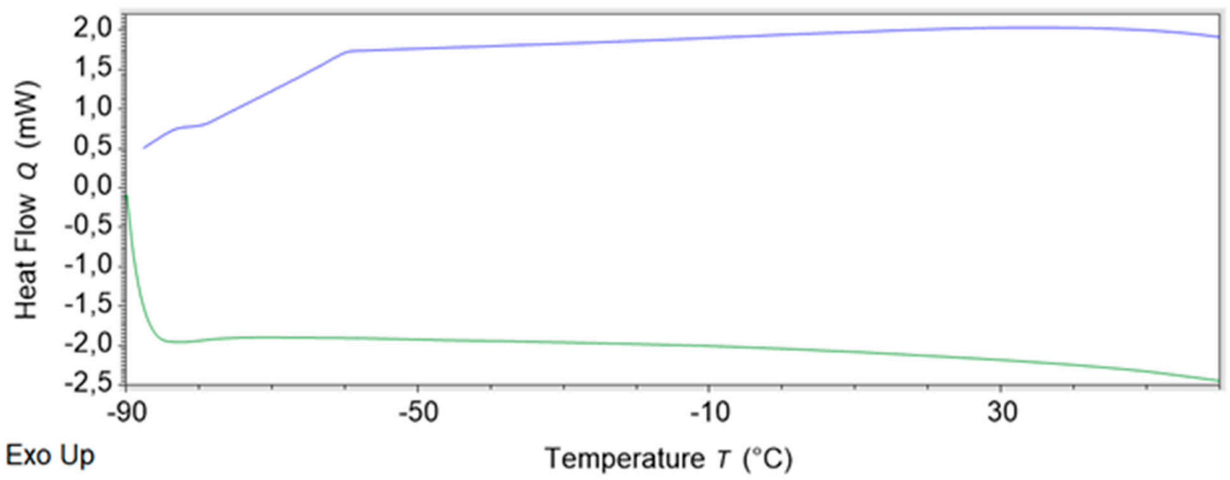

Figure S52. DSC of L-Carn:EG 1:2 DES. The heating run at 10 °C/min is reported in green while the cooling run in blue.

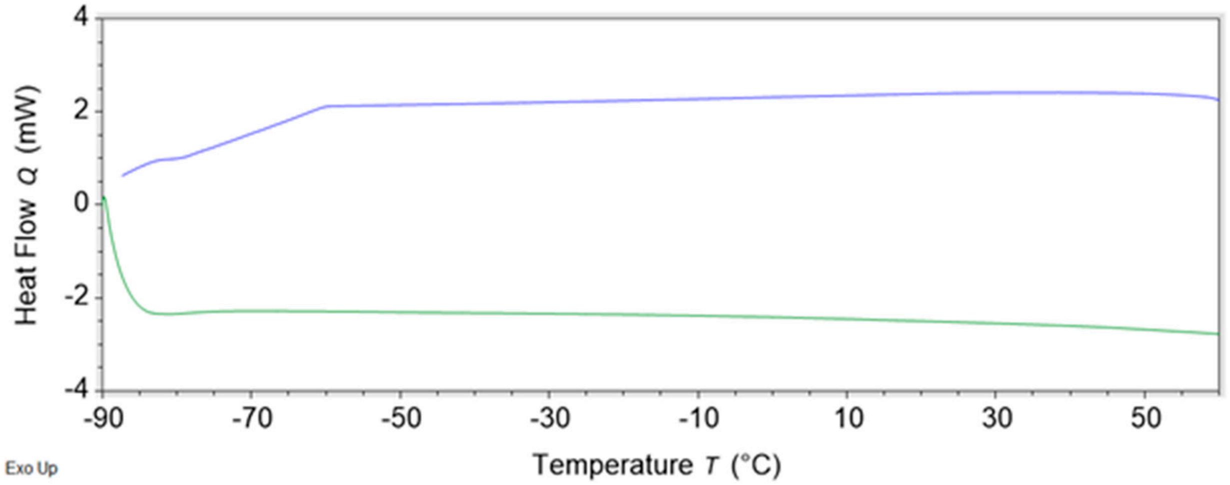

Figure S53. DSC of L-Carn:EG 1:3 DES. The heating run at 10 °C/min is reported in green while the cooling run in blue.

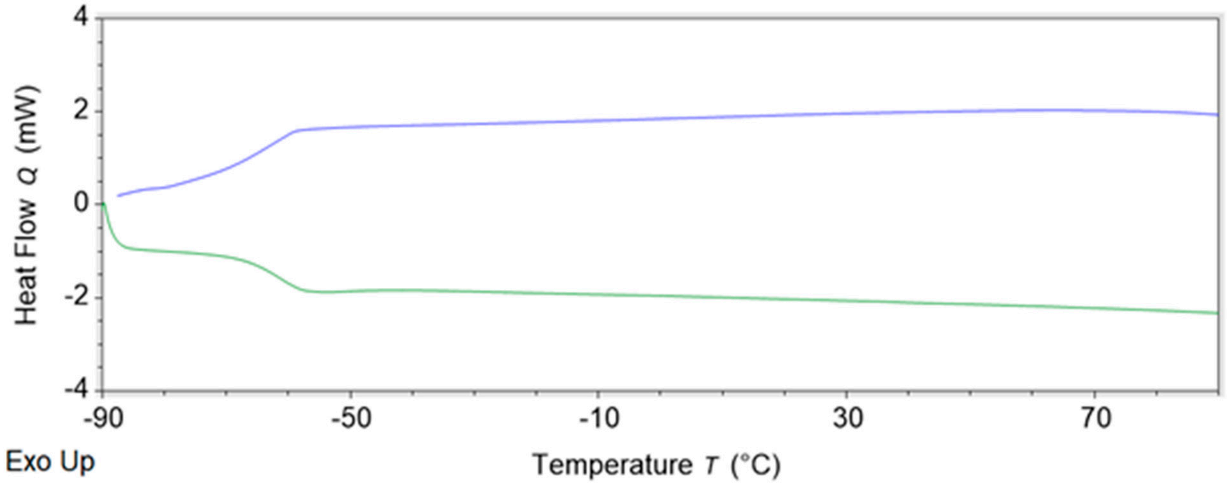

Figure S54. DSC of L-Carn:Gly 1:2 DES. The heating run at 10 °C/min is reported in green while the cooling run in blue.

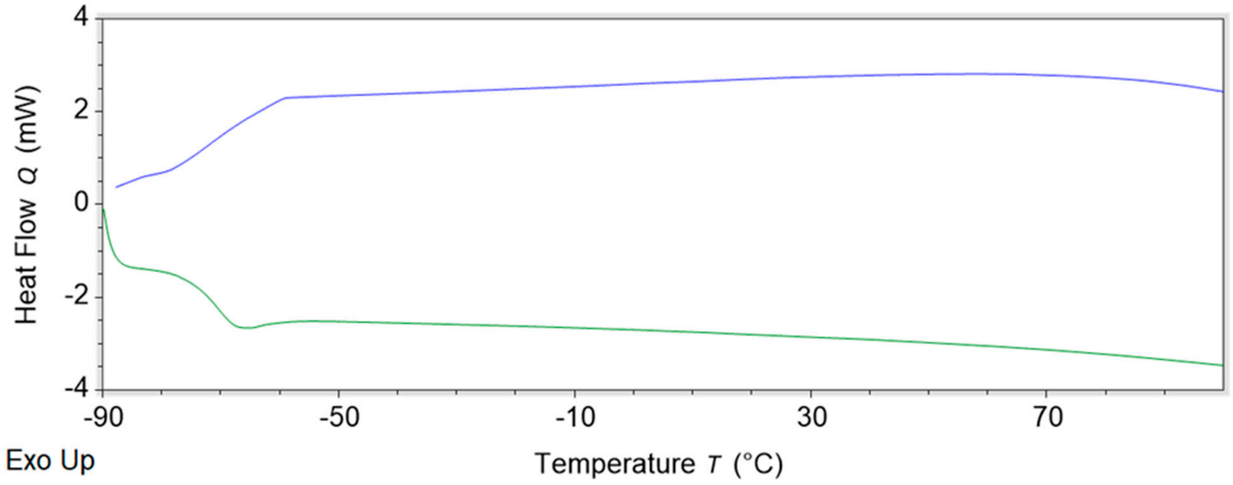

Figure S55. DSC of L-Carn:Gly 1:3 DES. The heating run at 10 °C/min is reported in green while the cooling run in blue.

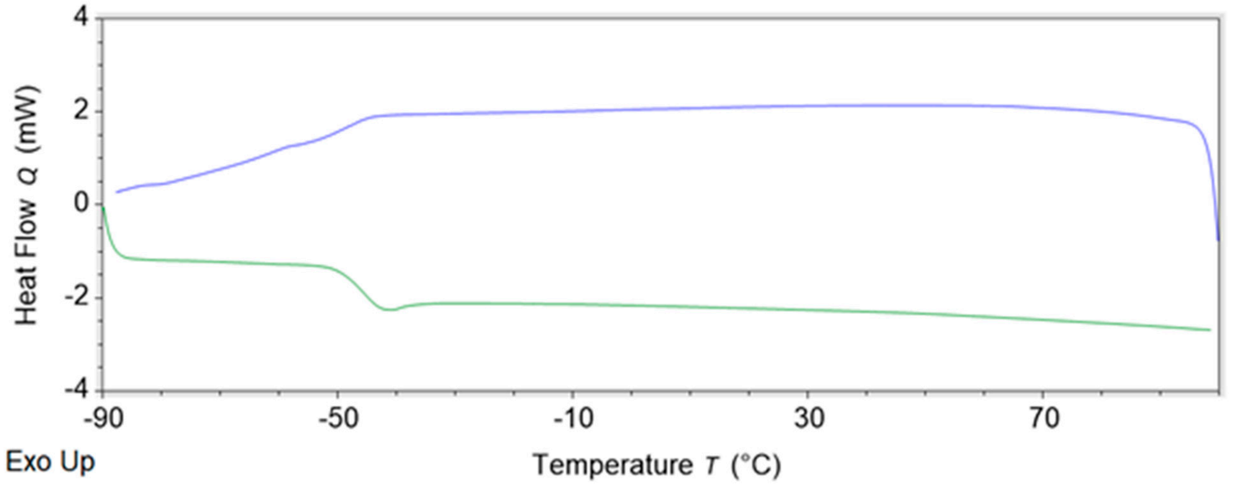

Figure S56. DSC of L-Carn:LevA 1:2 DES. The heating run at 10 °C/min is reported in green while the cooling run in blue.

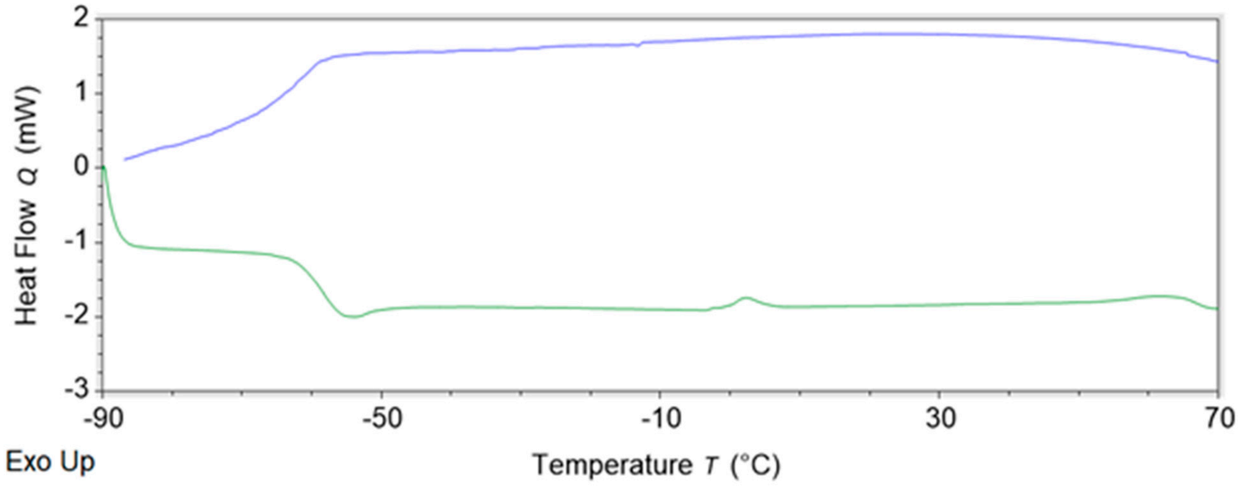

Figure S57. DSC of L-Carn:LevA 1:3 DES. The heating run at 10 °C/min is reported in green while the cooling run in blue.

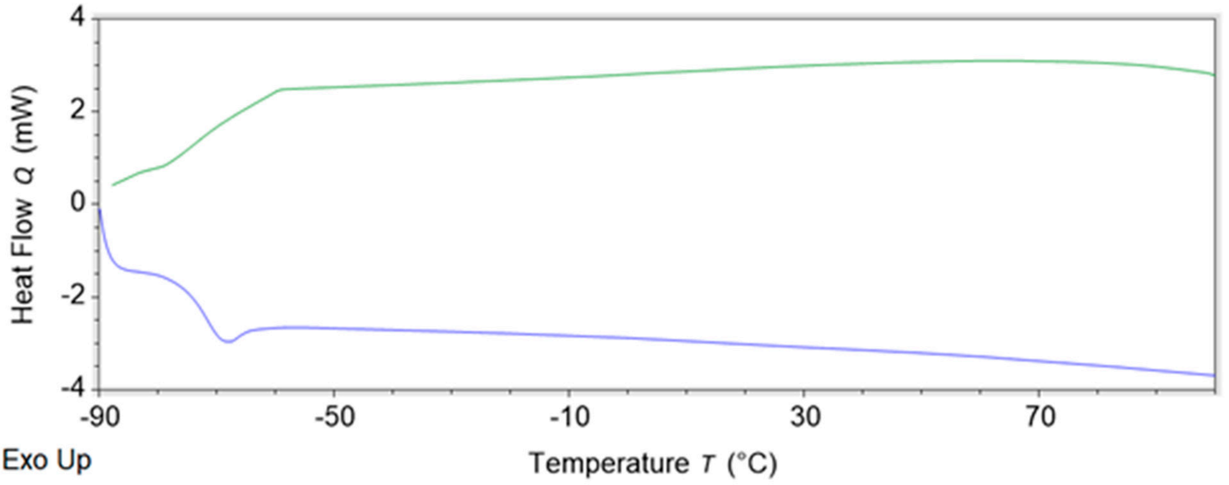

Figure S58. DSC of L-Pro:Gly 1:3 DES. The heating run at 10 °C/min is reported in green while the cooling run in blue.

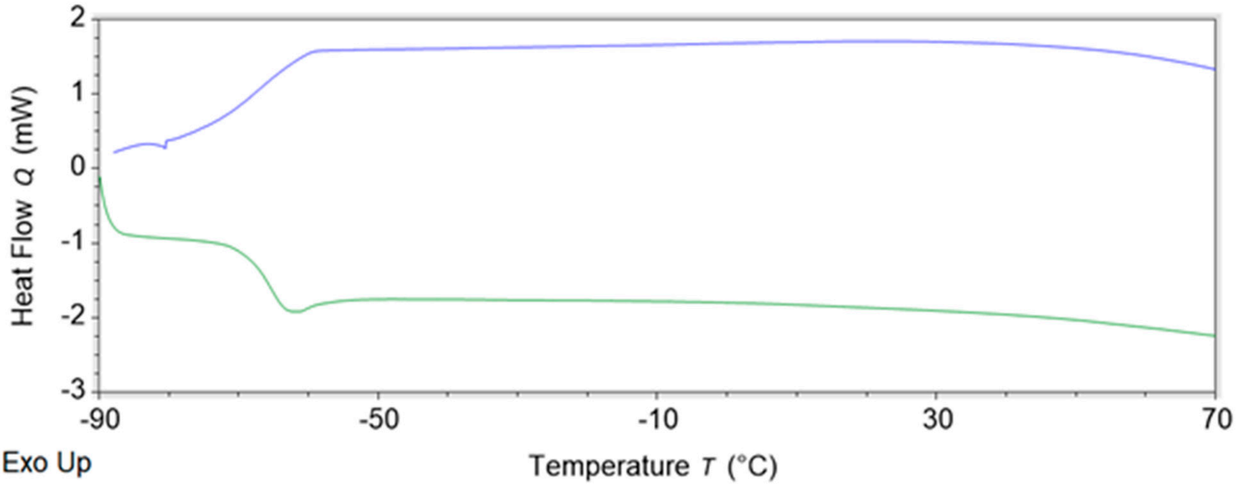

Figure S59. DSC of L-Pro:LevA 1:3 DES. The heating run at 10 °C/min is reported in green while the cooling run in blue.

Table S10. Measured and predicted refractive index values at the five wavelengths and four temperatures of measurement.

DES	T(°C)	450nm		532nm		632.8nm		964nm		1551nm	
		Meas.	Predict.	Meas.	Predict.	Meas.	Predict.	Meas.	Predict.	Meas.	Predict.
ChOAc:EG (1:2)	40	1.4610	1.4605	1.4550	1.4546	1.4504	1.4503	1.4442	1.4440	1.4391	1.4395
	60	1.4559	1.4558	1.4491	1.4500	1.4455	1.4457	1.4397	1.4394	1.4349	1.4349
	80	1.4517	1.4511	1.4437	1.4453	1.4411	1.4410	1.4350	1.4348	1.4303	1.4304
	100	1.4471	1.4463	1.4390	1.4406	1.4369	1.4364	1.4312	1.4302	1.4258	1.4258
ChOAc:EG (1:3)	40	1.4561	1.4557	1.4493	1.4499	1.4459	1.4456	1.4396	1.4394	1.4344	1.4349
	60	1.4510	1.4507	1.4442	1.4450	1.4409	1.4408	1.4349	1.4346	1.4302	1.4302
	80	1.4464	1.4457	1.4392	1.4401	1.4362	1.4359	1.4302	1.4298	1.4255	1.4253
	100	1.4412	1.4408	1.4339	1.4351	1.4310	1.4310	1.4252	1.4249	1.4205	1.4205
ChOAc:Gly (1:2)	40	1.4783	1.4783	1.4725	1.4722	1.4678	1.4677	1.4611	1.4611	1.4559	1.4560
	60	1.4734	1.4736	1.4677	1.4675	1.4628	1.4631	1.4566	1.4565	1.4514	1.4514
	80	1.4687	1.4689	1.4631	1.4629	1.4581	1.4584	1.4518	1.4519	1.4468	1.4468
	100	1.4641	1.4641	1.4586	1.4582	1.4536	1.4538	1.4473	1.4473	1.4424	1.4422
ChOAc:Gly (1:3)	40	1.4777	1.4778	1.4719	1.4717	1.4671	1.4672	1.4607	1.4606	1.4553	1.4555
	60	1.4731	1.4729	1.4670	1.4669	1.4622	1.4625	1.4562	1.4559	1.4507	1.4508
	80	1.4681	1.4681	1.4625	1.4622	1.4576	1.4578	1.4513	1.4512	1.4461	1.4461
	100	1.4634	1.4633	1.4568	1.4574	1.4529	1.4530	1.4467	1.4465	1.4416	1.4414
ChOAc:LevA (1:2)	40	1.4632	1.4632	1.4568	1.4568	1.4520	1.4522	1.4453	1.4455	1.4407	1.4411
	60	1.4574	1.4571	1.4511	1.4508	1.4467	1.4462	1.4398	1.4396	1.4351	1.4353
	80	1.4515	1.4510	1.4438	1.4448	1.4407	1.4402	1.4340	1.4337	1.4297	1.4294
	100	1.4449	1.4449	1.4380	1.4387	1.4339	1.4342	1.4279	1.4278	1.4236	1.4235
ChOAc:LevA (1:3)	40	1.4609	1.4612	1.4551	1.4547	1.4497	1.4500	1.4433	1.4433	1.4387	1.4391
	60	1.4548	1.4549	1.4492	1.4485	1.4439	1.4438	1.4373	1.4372	1.4327	1.4330
	80	1.4486	1.4485	1.4421	1.4422	1.4376	1.4376	1.4315	1.4311	1.4273	1.4268
	100	1.4420	1.4421	1.4361	1.4359	1.4307	1.4313	1.4245	1.4249	1.4208	1.4207
L-Carn:EG (1:2)	40	1.4773	1.4774	1.4709	1.4711	1.4665	1.4664	1.4596	1.4596	1.4546	1.4549
	60	1.4724	1.4724	1.4661	1.4661	1.4616	1.4614	1.4548	1.4547	1.4500	1.4500
	80	1.4674	1.4673	1.4612	1.4611	1.4569	1.4565	1.4499	1.4498	1.4453	1.4451
	100	1.4621	1.4622	1.4562	1.4560	1.4510	1.4515	1.4445	1.4448	1.4403	1.4402
L-Carn:EG (1:3)	40	1.4766	1.4763	1.4701	1.4702	1.4662	1.4657	1.4588	1.4591	1.4538	1.4540
	60	1.4715	1.4713	1.4648	1.4653	1.4610	1.4608	1.4541	1.4543	1.4491	1.4492
	80	1.4662	1.4663	1.4598	1.4603	1.4561	1.4559	1.4496	1.4494	1.4444	1.4443
	100	1.4613	1.4612	1.4550	1.4553	1.4508	1.4510	1.4449	1.4445	1.4395	1.4394
L-Carn:Gly (1:2)	40	1.4997	1.4999	1.4938	1.4936	1.4897	1.4890	1.4813	1.4820	1.4761	1.4763
	60	1.4949	1.4951	1.4884	1.4889	1.4850	1.4843	1.4778	1.4773	1.4713	1.4717
	80	1.4902	1.4902	1.4837	1.4841	1.4802	1.4795	1.4724	1.4726	1.4674	1.4670
	100	1.4856	1.4854	1.4794	1.4793	1.4742	1.4747	1.4673	1.4679	1.4627	1.4623
L-Carn:Gly (1:3)	40	1.4919	1.4919	1.4870	1.4858	1.4806	1.4813	1.4753	1.4748	1.4692	1.4699
	60	1.4874	1.4871	1.4807	1.4811	1.4760	1.4767	1.4707	1.4702	1.4650	1.4653
	80	1.4830	1.4824	1.4758	1.4764	1.4712	1.4720	1.4663	1.4655	1.4607	1.4607
	100	1.4778	1.4776	1.4710	1.4717	1.4669	1.4673	1.4617	1.4609	1.4564	1.4561
L-Carn:LevA (1:2)	40	1.4821	1.4823	1.4761	1.4756	1.4707	1.4708	1.4639	1.4638	1.4589	1.4590
	60	1.4766	1.4767	1.4700	1.4701	1.4651	1.4653	1.4585	1.4584	1.4536	1.4537
	80	1.4712	1.4712	1.4649	1.4647	1.4597	1.4599	1.4532	1.4530	1.4484	1.4483
	100	1.4657	1.4656	1.4594	1.4591	1.4539	1.4544	1.4476	1.4476	1.4430	1.4429
L-Carn:LevA (1:3)	40	1.4738	1.4740	1.4675	1.4674	1.4623	1.4626	1.4558	1.4557	1.4509	1.4511
	60	1.4681	1.4680	1.4623	1.4616	1.4566	1.4568	1.4501	1.4500	1.4452	1.4454
	80	1.4619	1.4621	1.4561	1.4557	1.4507	1.4510	1.4445	1.4443	1.4398	1.4396
	100	1.4560	1.4562	1.4501	1.4498	1.4446	1.4452	1.4385	1.4385	1.4340	1.4339
L-Pro:Gly (1:3)	40	1.4963	1.4962	1.4905	1.4899	1.4851	1.4852	1.4781	1.4782	1.4722	1.4726
	60	1.4910	1.4912	1.4854	1.4849	1.4799	1.4803	1.4733	1.4733	1.4675	1.4677
	80	1.4858	1.4861	1.4803	1.4799	1.4751	1.4753	1.4684	1.4683	1.4630	1.4627
	100	1.4808	1.4810	1.4747	1.4748	1.4700	1.4703	1.4635	1.4634	1.4581	1.4578
L-Pro:LevA (1:3)	40	1.4720	1.4720	1.4653	1.4653	1.4601	1.4605	1.4535	1.4535	1.4483	1.4486
	60	1.4655	1.4654	1.4594	1.4588	1.4540	1.4540	1.4472	1.4471	1.4421	1.4423
	80	1.4588	1.4588	1.4522	1.4523	1.4477	1.4475	1.4409	1.4407	1.4363	1.4359
	100	1.4518	1.4521	1.4458	1.4457	1.4405	1.4410	1.4341	1.4342	1.4294	1.4294

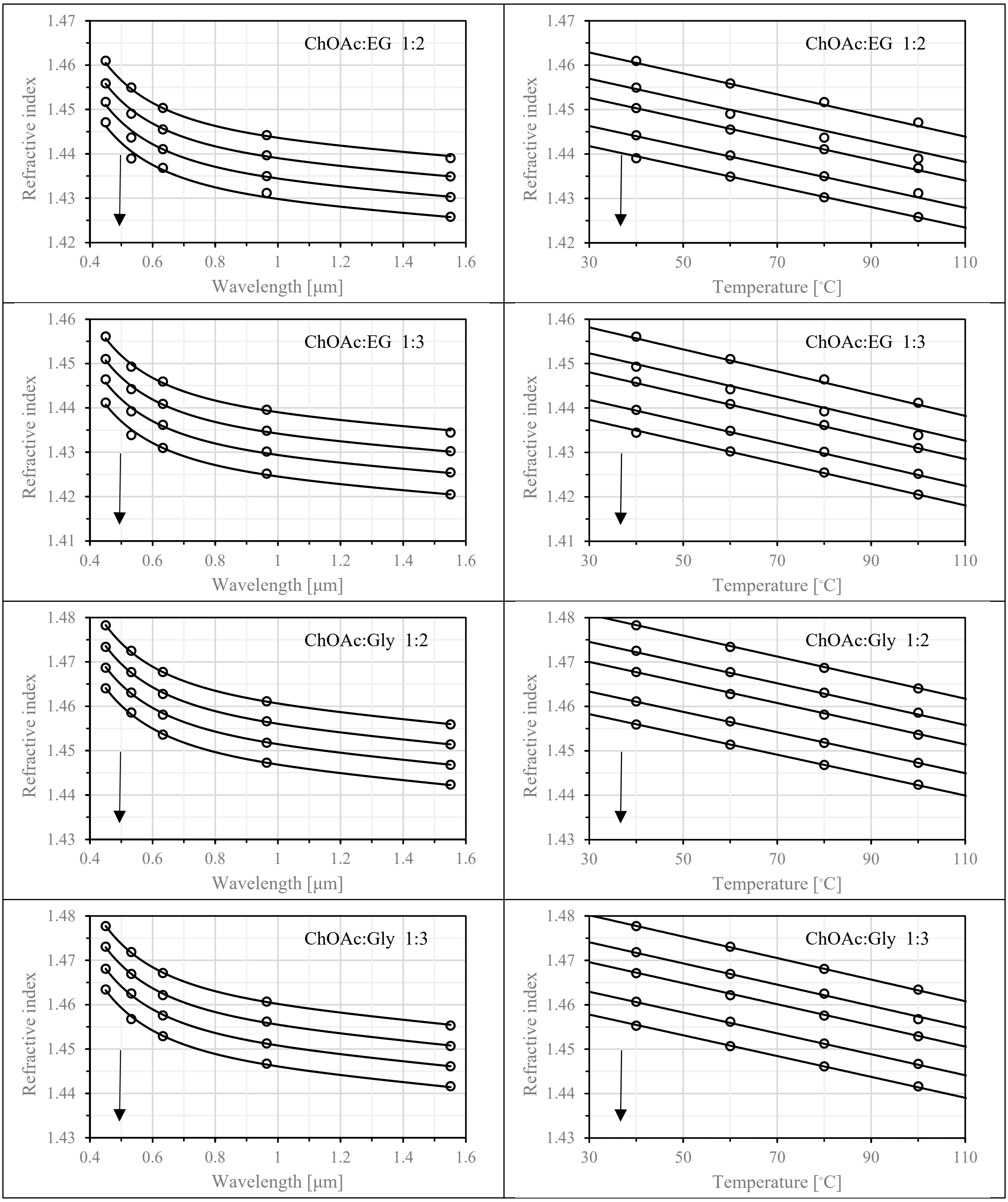
ChOAc:EG 1:2
ChOAc:EG 1:3
ChOAc:Gly 1:2
ChOAc:Gly 1:3
Refractive index
Wavelength [μm]
Temperature [°C]
0.4
0.6
0.8
1
1.2
1.4
1.6
30
50
70
90
110
1.41
1.42
1.43
1.44
1.45
1.46
1.47
1.48

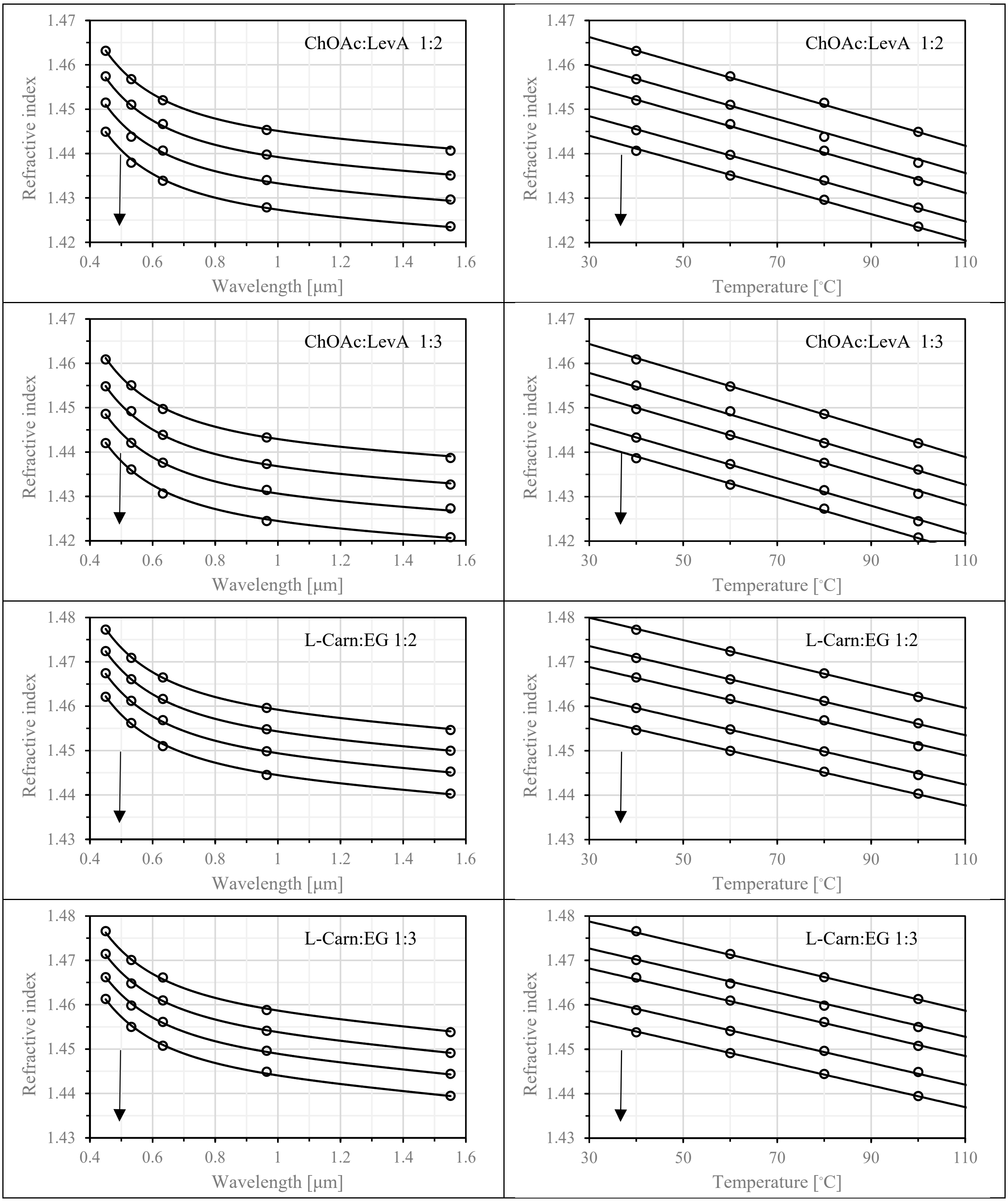
ChOAc:LevA 1:2
ChOAc:LevA 1:3
L-Carn:EG 1:2
L-Carn:EG 1:3
Refractive index
Wavelength [μm]
Temperature [°C]
0.4
0.6
0.8
1
1.2
1.4
1.6
30
50
70
90
110
1.42
1.43
1.44
1.45
1.46
1.47
1.48

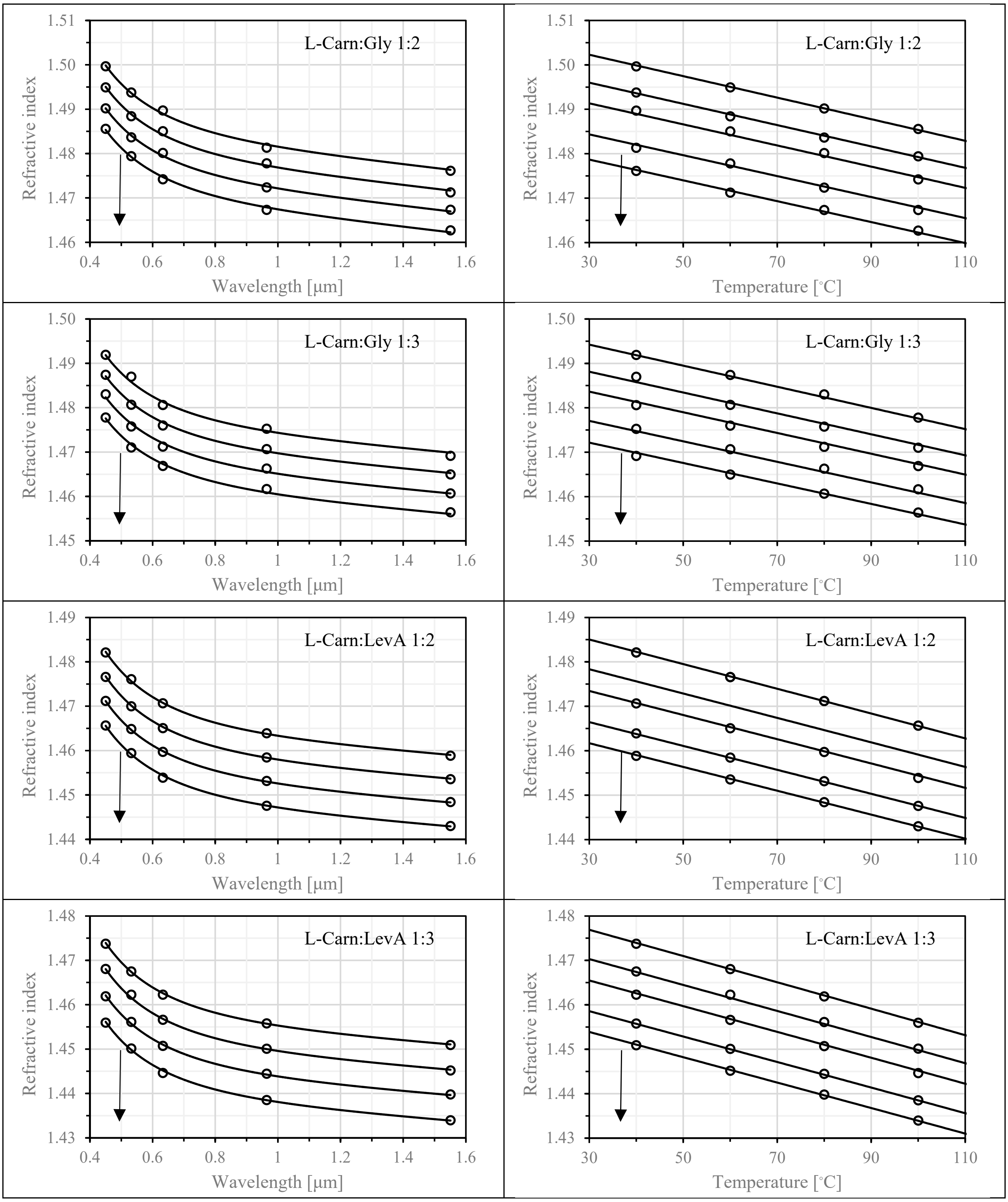
L-Carn:Gly 1:2
L-Carn:Gly 1:3
L-Carn:LevA 1:2
L-Carn:LevA 1:3
Refractive index
Wavelength [μm]
Temperature [°C]
0.4
0.6
0.8
1
1.2
1.4
1.6
30
50
70
90
110
1.43
1.44
1.45
1.46
1.47
1.48
1.49
1.50
1.51

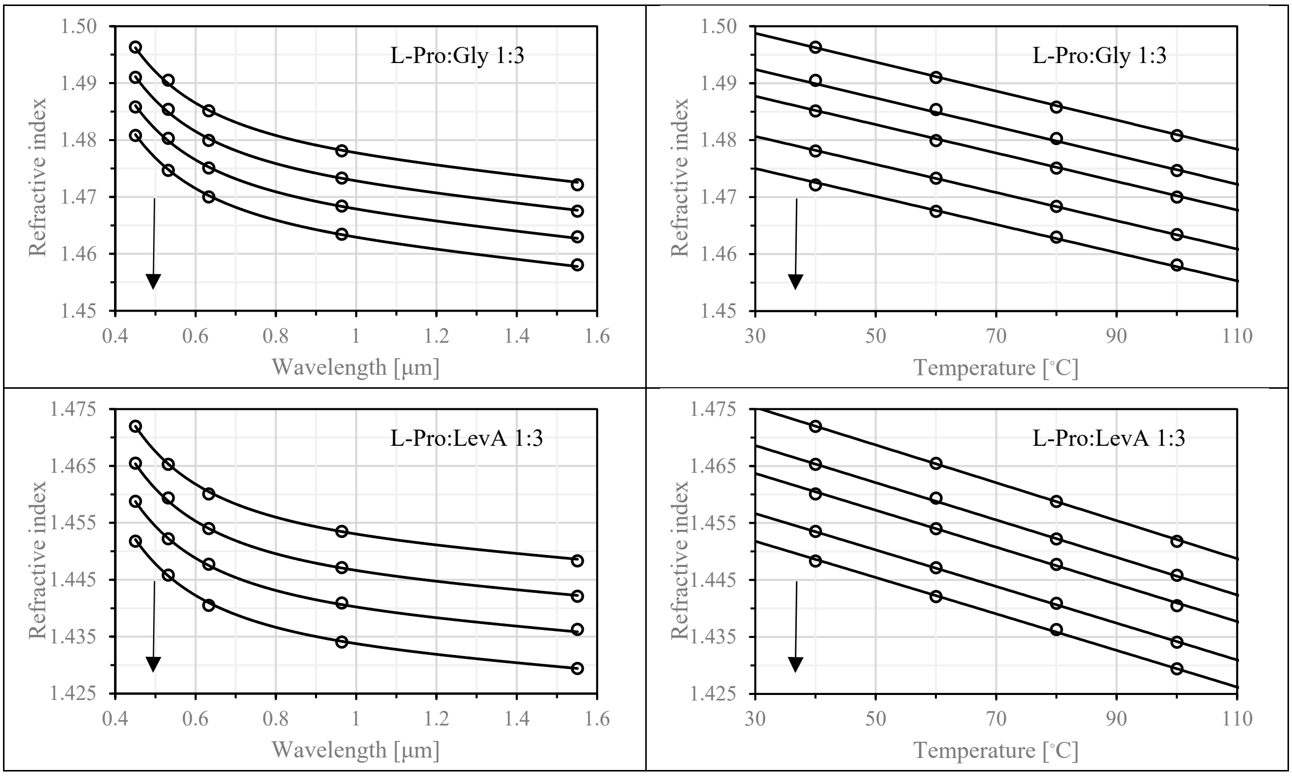

Figure S60. Refractive index as a function of wavelength (left plots) and temperature (right plots) for the various DESs under investigation, as is indicated. In the left plots, arrows indicate the direction of increasing temperature (40 °C, 60 °C, 80 °C and 100 °C). In the right plots, arrows indicate the direction of increasing wavelength (450 nm, 532 nm, 632.8 nm, 964 nm and 1551 nm). Open circles: Experimental data. Solid lines: Predicted values based on the proposed dispersion model.
